# Microbiome of Pacific Whiteleg shrimp reveals differential bacterial community composition between Wild, Aquacultured and AHPND/EMS outbreak conditions

**DOI:** 10.1038/s41598-017-11805-w

**Published:** 2017-09-18

**Authors:** Fernanda Cornejo-Granados, Alonso A. Lopez-Zavala, Luigui Gallardo-Becerra, Alfredo Mendoza-Vargas, Filiberto Sánchez, Rodrigo Vichido, Luis G. Brieba, Maria Teresa Viana, Rogerio R. Sotelo-Mundo, Adrián Ochoa-Leyva

**Affiliations:** 1 Departamento de Microbiología Molecular, Instituto de Biotecnología (IBT), Universidad Nacional Autónoma de México (UNAM), Av. Universidad #2001, Col. Chamilpa, Cuernavaca, Morelos, 62210 Mexico; 2 Departamento de Ciencias Químico Biológicas, Universidad de Sonora (UNISON). Blvd., Rosales y Luis Encinas, Hermosillo, Sonora, 83000 Mexico; 3 Instituto Nacional de Medicina Genómica, Secretaría de Salud (INMEGEN), Periférico Sur No. 4809, Mexico, D.F. 14610 Mexico; 4 Centro Nacional de Servicios de Constatación en Salud Animal (CENAPA), Carr. Fed. Cuernavaca-Cuautla No. 8534 Jiutepec, Morelos, 8534 Mexico; 5 Laboratorio Nacional de Genómica para la Biodiversidad (LANGEBIO), Centro de Investigación y Estudios Avanzados (CINVESTAV Unidad Irapuato) Km 9.6 Libramiento Norte Carretera Irapuato-León, Apartado Postal 629, Irapuato, Guanajuato, 36500 Mexico; 6 Instituto de Investigaciones Oceanológicas, Universidad Autónoma de Baja California (UABC), Ensenada, B.C. Mexico; 7 Laboratorio de Estructura Biomolecular, Centro de Investigación en Alimentación y Desarrollo, A.C. (CIAD), Carretera a Ejido La Victoria Km 0.6, Apartado Postal 1735, Hermosillo, Sonora 83304 Mexico

## Abstract

Crustaceans form the second largest subphylum on Earth, which includes *Litopeneaus vannamei* (Pacific whiteleg shrimp), one of the most cultured shrimp worldwide. Despite efforts to study the shrimp microbiota, little is known about it from shrimp obtained from the open sea and the role that aquaculture plays in microbiota remodeling. Here, the microbiota from the hepatopancreas and intestine of wild type (wt) and aquacultured whiteleg shrimp and pond sediment from hatcheries were characterized using sequencing of seven hypervariable regions of the 16S rRNA gene. Cultured shrimp with AHPND/EMS disease symptoms were also included. We found that (i) microbiota and their predicted metagenomic functions were different between wt and cultured shrimp; (ii) independent of the shrimp source, the microbiota of the hepatopancreas and intestine was different; (iii) the microbial diversity between the sediment and intestines of cultured shrimp was similar; and (iv) associated to an early development of AHPND/EMS disease, we found changes in the microbiome and the appearance of disease-specific bacteria. Notably, under cultured conditions, we identified bacterial taxa enriched in healthy shrimp, such as *Faecalibacterium prausnitzii* and *Pantoea agglomerans*, and communities enriched in diseased shrimp, such as *Aeromonas taiwanensis*, *Simiduia agarivorans* and *Photobacterium angustum*.

## Introduction

In terrestrial organisms, the initial bacterial colonization occurs from the maternal microbiota, while in aquatic systems, the colonization comes from the surrounding water and sediment^1^. The microbiota plays a significant role in the development and physiology of its host, preventing the growth of pathogenic bacteria, modulating the immune response, affecting nutrient absorption, regulating metabolic processes, and synthesizing vitamins^2^. The structure (abundance and composition) and function of the microbiota are influenced by feed intake, probiotics, prebiotics, hormone secretion, stress, antibiotics, developmental stage, environmental and physiological conditions^2^, the host metabolism and the immune response^3^. Crustaceans belong to the arthropod group, which represents the second largest subphylum on Earth; it includes crabs, lobsters, crayfish, krill, and shrimp. Artificial rearing of Crustacea has led to a growing aquaculture industry that has obviated the capture of this resource, although its culture could have an adverse impact on marshes and coastal mangrove areas^4^.

As in many areas of animal nutrition, a great emphasis is now placed on understanding the roles of microbiota in the health, growth, and survival of cultured organisms^5^. In early studies, conventional culture-dependent techniques were used to characterize bacterial communities in Crustacea, followed by traditional molecular approaches, such as denaturing gradient gel electrophoresis (DGGE) and clone libraries^6–10^. More recently, the availability and low cost of high-throughput sequencing of the small ribosomal subunit 16S (16S rRNA) gene have facilitated a more in-depth analysis of shrimp microbiota under cultured conditions. A great diversity of microbiota that could not be identified with traditional culture-dependent methods was discovered. Changes in the microbiota of shrimp species, such as *Penaeus monodon* (black tiger shrimp)^11^, *Fenneropenaeus chinensis* (Chinese shrimp)^10^, *Penaeus penicillatus* ^12^, *Penaeus merguiensis* (banana shrimp)^13^, and *Litopenaeus vannamei* ^14^ (Pacific whiteleg shrimp), have been described under a variety of growth and water quality conditions. However, the microbiota from natural environments has only been studied in the intestine of *P*. *monodon* ^11^. To our knowledge, the intestine and hepatopancreas microbiota and their potential functions in wild type (wt) *L*. *vannamei* captured in the Pacific Ocean are unknown. Thus, the characterization of the natural *L*. *vannamei* microbiota, to be used as a reference for comparison with healthy and diseased cultured shrimp in hatcheries, is necessary. Hence, modification of the microbiota could be utilized as a new approach against shrimp diseases^15^.

There is scarce information about the microbiota in shrimp organs other than the intestine, leaving aside the important connection that exists between the function of this organ and the hepatopancreas because the crustacean digestive tract is continuous^16^. The hepatopancreas is an essential organ for digestion and absorption of nutrients, and it plays a role in innate immunity in invertebrates^17^. To date, there has been only one study examining the microbiota of the hepatopancreas in *N*. *denticulata* shrimp^16^. Moreover, the hepatopancreas can be drastically affected by pathogens such as *V*. *parahemolyticus*, which causes the devastating early mortality syndrome (EMS)^18,19^, also called acute hepatopancreatic necrosis disease (AHPND), in shrimp^20^. The AHPND/EMS is hampering global shrimp aquaculture, and a great effort has been undertaken to understand the molecular basis of the disease, including genomic studies of *V*. *parahaemoluticus* ^21^. A seminal study demonstrated that *Pir A/B* toxins are associated with the presence of AHPND/EMS, targeting the hepatopancreas, and their presence explains many of the phenotypic aspects of the disease^18^. To our knowledge, there have been no studies of the effects of bacterial toxins on the microbiota, although some authors have shown that chemical toxins indeed have effects on animal bacterial communities^22^. Few studies have quantified how the microbiota is affected by the emergence of disease under aquaculture conditions^23,24^. Pathogens can interact with the host microbiota by competition for resources, release of antimicrobial compounds or antagonism. Hence, a pathogen can cause microbiota dysbiosis. Due to the constant interaction between the shrimp digestive system and its surrounding environment (water and sediment), the microbiota of the intestine and hepatopancreas should participate in disease resistance mechanisms. However, there is no published research describing changes in the microbiota in shrimp during an AHPND/EMS outbreak.

**Figure 1:**
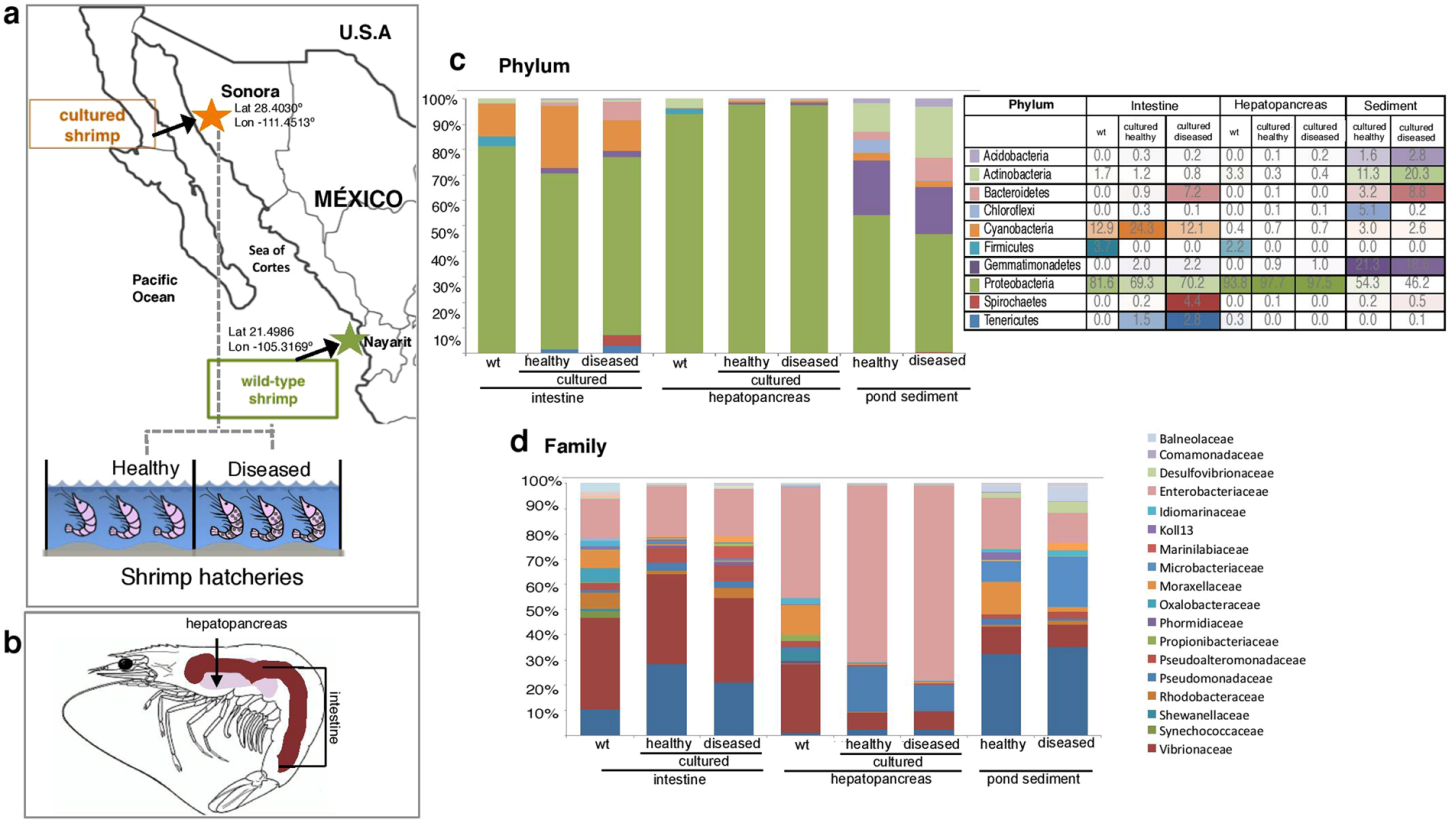
Shrimp sample collection and general microbiota characteristics. (**a**) *Litopenaeus vannamei* specimen collection points. Wt shrimp were collected from the Mexican Pacific area 5 km from the state coast (green star), while cultured shrimp were collected from hatchery ponds (orange star) located in the state of Sonora, Mexico. Healthy and AHPND/EMS-diseased shrimp were collected from ponds adjacent to each other. The map was created using the online edition of SmartDraw https://www.smartdraw.com/. (**b**) Anatomical location of the dissected hepatopancreas and intestine. Taxonomic diversity and abundance of all 24 of the sequenced samples at: (**c**) phylum; and (**d**) family levels.

*L*. *vannamei*, commonly known as Pacific whiteleg shrimp, is one of the most cultured shrimp species (>70%) worldwide, and it contributes importantly to the economic development of coastal wetland areas^25^. In this study, we present the structure and function of the intestine and hepatopancreas microbiota of Pacific whiteleg shrimp samples from their natural environment and from aquaculture hatcheries, including pond sediments (Fig. 1). Our study incorporates the sequencing of seven hypervariable regions of the 16S ribosomal gene. Additionally, we included the microbiota analysis of shrimp with AHPND/EMS symptoms. Our main goals were to determine: (i) the structure and function of the microbiota between shrimp from wt and cultured environments; (ii) the impact of AHPND/EMS disease on the microbiota structure and function from the hepatopancreas and intestine of cultured shrimp; and (iii) the analysis of microbiota composition between hatchery pond sediments containing healthy and diseased shrimp. To our knowledge, this study was the first high-throughput sequencing study of *L*. *vannamei* microbiota using individual intestines and hepatopancreases from natural and cultured environments, including microbiota associated with the early development of APHND/EMS disease.

## Results

### Read classification per hypervariable region of 16S rRNA

The ION 16S™ Metagenomics kit includes six proprietary primers designed to target the V2, V4 and V8 regions in one simultaneous PCR reaction and the V3, V6-7, and V9 regions in a second reaction. The percentage of reads was uniform for all of the regions among three biological replicates of each group (Supplementary Fig. S1). We observed that regions V2, V4, V8, and V9 were not biased in read abundance towards any particular sample group. However, the reads in the V3 region were significantly enriched for wt hepatopancreas (*p* < 0.05). In contrast, the reads in V6-V7 were significantly enriched for cultured intestines and hepatopancreases (Supplementary Fig. S2). Interestingly, this bias depended on the shrimp origin, suggesting that there is more different abundance of specific microbial taxa (with biased amplification on the V3, V6-7 regions) between wt and cultured samples. Interestingly, the assigned operational taxonomic units (OTUs) were different among the seven sequenced regions (Supplementary Fig. S3).

### General microbiome characteristics using seven hypervariable regions

After singleton removal, 1,006 OTUs were obtained, and they were assigned to 36 phyla, 81 classes, 152 orders, 234 families, 489 genera and 298 species with taxonomical names. Approximately 47% of the reads did not have a match to any known OTU reported in the GreenGenes database (Supplementary Fig. S4a). Interestingly, the pond sediment samples had the largest number of unknown reads (~60%), followed by wt shrimp samples (Supplementary Fig. S4b). The bulk of OTU abundance was variable: the phylum *Proteobacteria* ranged from 28.2 to 98.6%, *Cyanobacteria* from 0.0 to 62.5%, *Actinobacteria* from 0.1 to 50.5%, *Gemmatimonadetes* from 0.0 to 44.7%, *Bacteroidetes* from 0.0 to 16.7% and *Firmicutes* from 0.0 to 10.9% (Fig. 1c and Supplementary Table S1). *Vibrio*, *Pseudomonas*, *Photobacterium*, and *Acinetobacter* were the most abundant genera in the hepatopancreas and intestine, while *Acinetobacter*, *Vibrio*, *KSA1*, and *Pseudomonas* were the most abundant genera in sediment (Supplementary Table S2). *Enterobacteriaceae*, *Vibrionaceae*, *Pseudoalteromonadaceae*, *Moraxellaceae*, *Pseudomonadaceae*, and *Rhodobacteraceae* were the most abundant families across all of the samples (Fig. 1d). The bacterial communities among biological triplicates were similar in the OTUs with abundances greater than 0.1% (Spearman’s correlations in Supplementary Table S3).

**Figure 2:**
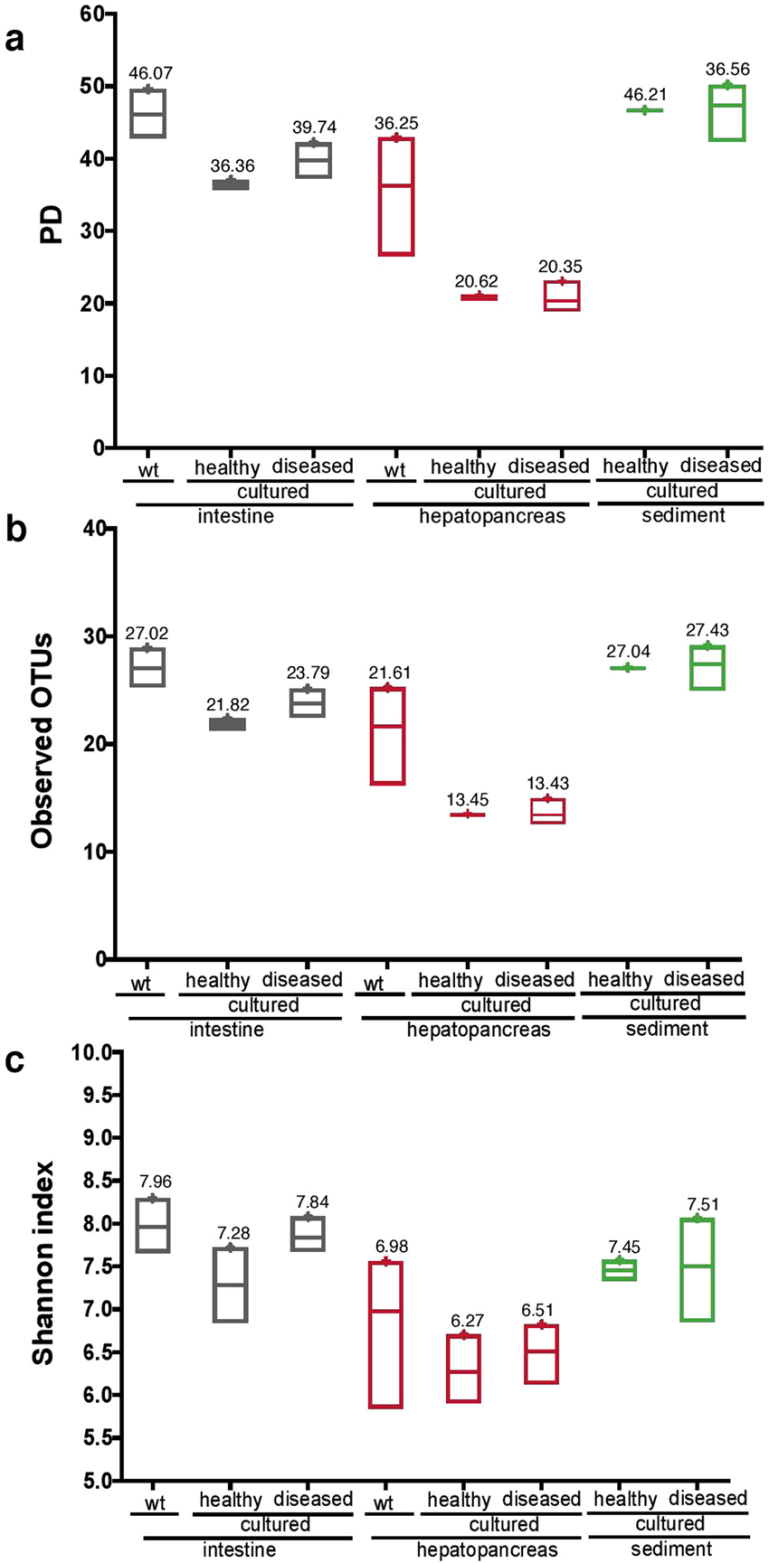
Alpha diversity indices. (**a**) Phylogenetic diversity (PD). (**b**) Number of observed OTUs. (**c**) Shannon index. All of the analyzed groups showed significant differences after a Mann-Whitney test with a confidence level of 99% (*p* ≤ 0.01) in each index. The average value for each group of samples (n = 3) is shown next to each boxplot, and the mean is indicated in red.

The alpha diversity metrics were calculated from the rarefaction curves at OTUs level for each group (Fig. 2). To increase the sequence depth, we discarded from this analysis two samples with low sequencing depth (68B-S and 139-I). Thus, the alpha diversity metrics were calculated at a sequence depth of 2,078 reads. Rarefaction curves showed that Shannon indices were relatively stable at the sampling depth of >2,000 reads (Supplementary Fig. S5a). All Chao1 values were greater than the observed OTUs, indicating that more OTUs could still be expected to be found in all of the samples (Supplementary Table S4). Accordingly, the Good’s coverage revealed that we obtained, on average, ~80% of the total OTUs (Supplementary Table S5), indicating that significant sequencing depth is necessary to discover the total OTUs in the analyzed microbiomes. The Good’s coverage was uniform between groups: 83% in average for intestines, 90% for hepatopancreases and 81% for sediments (Supplementary Table S5). The phylogenetic diversity (PD), Shannon index and observed OTUs showed that sediments and intestines had more bacterial diversity than hepatopancreases (Fig. 2). Rarefaction curves were obtained using only those sequences assigned to a genus as an estimate of diversity at that taxonomic level. The rarefaction curves did not reach saturation (Supplementary Fig. S5b), even when the sequences of the three samples from each ambient were pooled to obtain greater sequence sampling depth. This finding indicated that the observed genera for each ambient seems not saturated even after 16,000 reads (Supplementary Fig. S5c), however significantly more sequencing depth will be needed to discover additional genera. The bulk of diversity indices for all of the sequenced samples is described in Supplementary Table S4.

**Figure 3:**
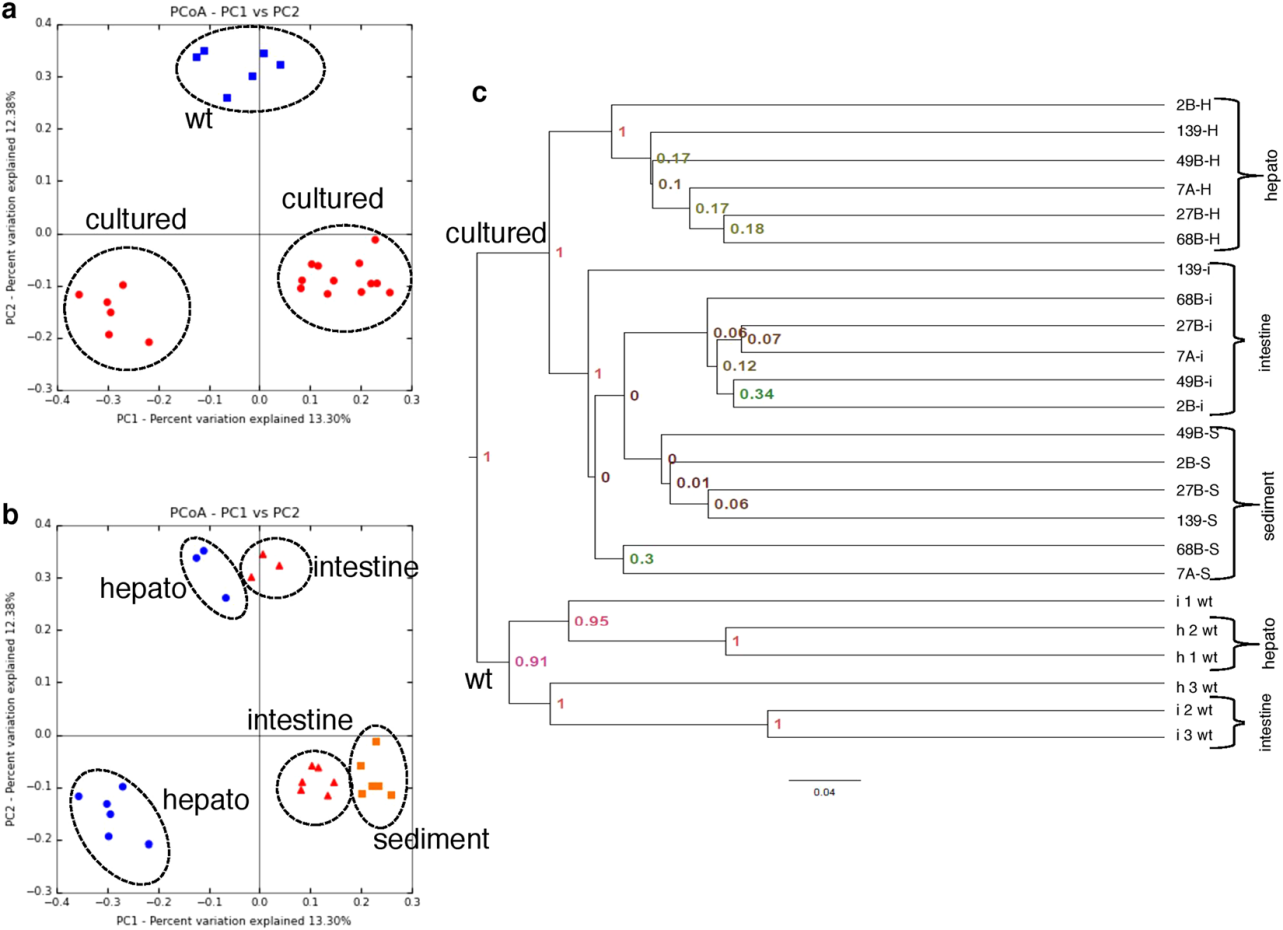
Beta diversity analysis. Unweighted principal coordinate analysis (PCoA) of UniFrac distances: (**a**) samples tagged as wt and cultured; and (**b**) samples tagged by shrimp organ or sediment. Only the first two components are shown. (**c**) UPGMA tree of unweighted UniFrac distances. Node value represents the jackknife support, using 1,000 replicates.

Principal component analysis (PCoA) using unweighted and weighted UniFrac distances showed that wt samples clustered separately from cultured samples (Fig. 3a and Supplementary Fig. S6a). Additionally, PCoA with samples tagged by organs and pond sediments formed different clusters (Fig. 3b). Bacterial communities in the hepatopancreas were distinct from those in the intestines whether from wt or cultured samples, and each organ clustered separately accordingly to its origin (Fig. 3b). Notably, intestine samples from aquacultured shrimp clustered together with pond sediment in the weighted PCoA (Supplementary Fig. S6b). Similar clusters were also observed in the UPGMA tree of unweighted UniFrac distances (Fig. 3c), in which all wt samples formed one cluster, and cultured samples formed another, both at 100% of Jackknife Support (JS) (Fig. 3c). Additionally, the hepatopancreas clustered separately from the intestine in the wt and cultured samples, while intestine and sediment clustered together at 100% JS in the cultured samples (Fig. 3c). This finding was in agreement with the close position between the intestine and sediment clusters observed in the unweighted PCoA (Fig. 3b) and with only one cluster observed containing all of the intestine and sediment samples in the weighted PCoA (Supplementary Fig. S6b). Therefore, the microbial diversity of pond sediments and shrimp intestines was similar under cultured shrimp conditions. The UPGMA weighted tree (Supplementary Fig. S7) showed similar results to those observed in the unweighted UPGMA tree (Fig. 3c).

### Intestine microbiome

**Figure 4:**
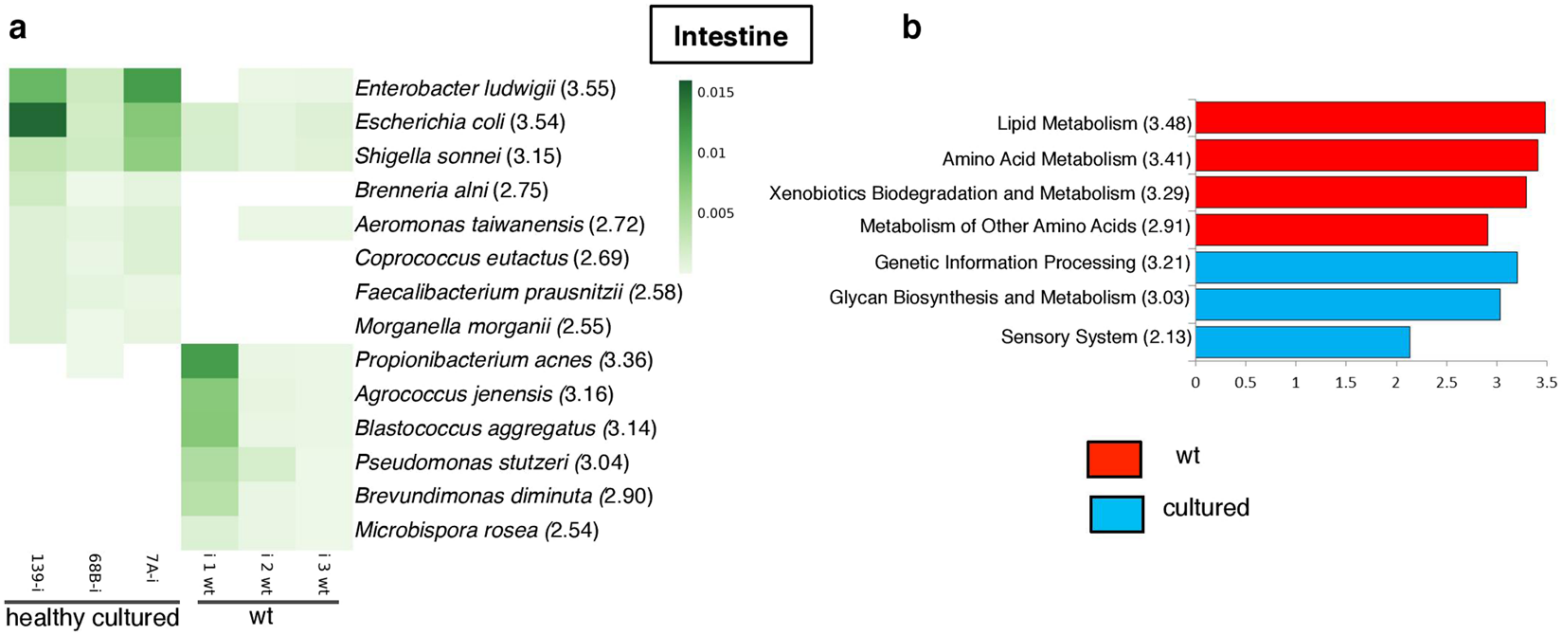
Relative abundance and LEfSe results of enriched microbiota and predicted microbiome functions between wt (n = 3) and cultured intestine (n = 3) samples. (**a**) Relative abundance of significantly different species. Linear discriminant analysis (LDA) score is shown in parenthesis. (**b**) LDA of the differentially abundant KEEG pathways. The differences were significant (*p* < 0.05) among classes (Kruskal-Wallis test) and subclasses (Wilcoxon’s test). The threshold of the logarithmic LDA score was 2.0.

All of the diversity indices (observed OTUs, PD and Shannon) showed a significantly (*p* < 0.05) larger biodiversity for the wt compared to healthy cultured intestines (Fig. 2 and Supplementary Table S4). We also found that both the wt and cultured intestines had significantly (*p* < 0.05) more bacterial diversity than wt and cultured hepatopancreases. *Proteobacteria* and *Cyanobacteria* were the most abundant phyla across most of the samples (Fig. 1c). *Vibrionaceae* and *Enterobacteriaceae* were the most abundant families, constituting ~50% of the total reads (Fig. 1d). At the genus level, the communities were rich in *Vibrio*, *Photobacterium*, and *Paracoccus* (Supplementary Table S2). The linear discriminant analysis (LDA) using LEfSe^26^, which uses the Kruskal-Wallis non-parametric and Wilcoxon-Mann-Whitney tests, was applied to identify biomarkers (taxa and metagenomic functions) accounting for the greatest differences between our different groups (n = 3). Because LEfSe requires all of the pairwise comparisons to reject the null hypothesis to detect a biomarker, no multiple testing corrections are needed^26^. After LEfSe analysis, the phyla *Actinobacteria* and *Nitrospirae* were significantly more abundant in wt samples (n = 3), while *Bacteroidetes*, *Gemmatimonadetes*, *Fusobacteria*, and *Spirochaetes* were more abundant in cultured samples (n = 3) (Supplementary Table S6). Classes, orders, families, genera, and species that were differentially abundant between wt and cultured samples are shown in Fig. 4a and Supplementary Table S6. The bacterial communities with abundance greater than 0.1% (OTUs > 0.1%) were very different between wt and healthy cultured shrimp (Spearman’s test, r = 0.026, p = 0.7144), in agreement with the findings observed in the PCoA and the UPGMA trees.

We used PICRUSt^27^ to predict the functionality of the shrimp metagenome using the 16S rRNA gene data. A total of 377 KEGG pathways were predicted using all of the sequenced samples (Supplementary Table S7). At level two of the functional subcategory, genes involved in four pathways (lipid and amino acid metabolism, xenobiotics biodegradation and metabolism of other amino acids) were significantly enriched in wt shrimp, while genetic information processing, sensory systems, glycan biosynthesis and metabolism-predicted pathways were enriched in healthy cultured samples (Fig. 4b). Interestingly, bacterial communities from cultured intestine samples had a significantly greater abundance of replication, recombination and repair proteins, and signal transduction pathways (Supplementary Fig. S8a). At level three of the functional subcategory, valine, leucine and isoleucine degradation, propanoate metabolism, fatty acid metabolism, benzoate degradation, and tryptophan metabolism were enriched in the microbiota derived from wt shrimp (Supplementary Fig. S8a). Interestingly, we found predicted genes involved in xenobiotics biodegradation, including styrene, caprolactam, aminobenzoate, naphthalene and bisphenol (Supplementary Fig. S8a). Additionally, to measure the reliability of the functional predictions for each sample, we calculated the nearest sequenced taxon index (NSTI) for PICRUSt predictions. This score estimates how closely related the microbes in a given sample are to representative microbes with sequenced genomes. The NSTI for wt and healthy intestines were 0.081 ± 0.035 and 0.112 ± 0.072, respectively (Supplementary Table S8).

### Hepatopancreas microbiome

**Figure 5:**
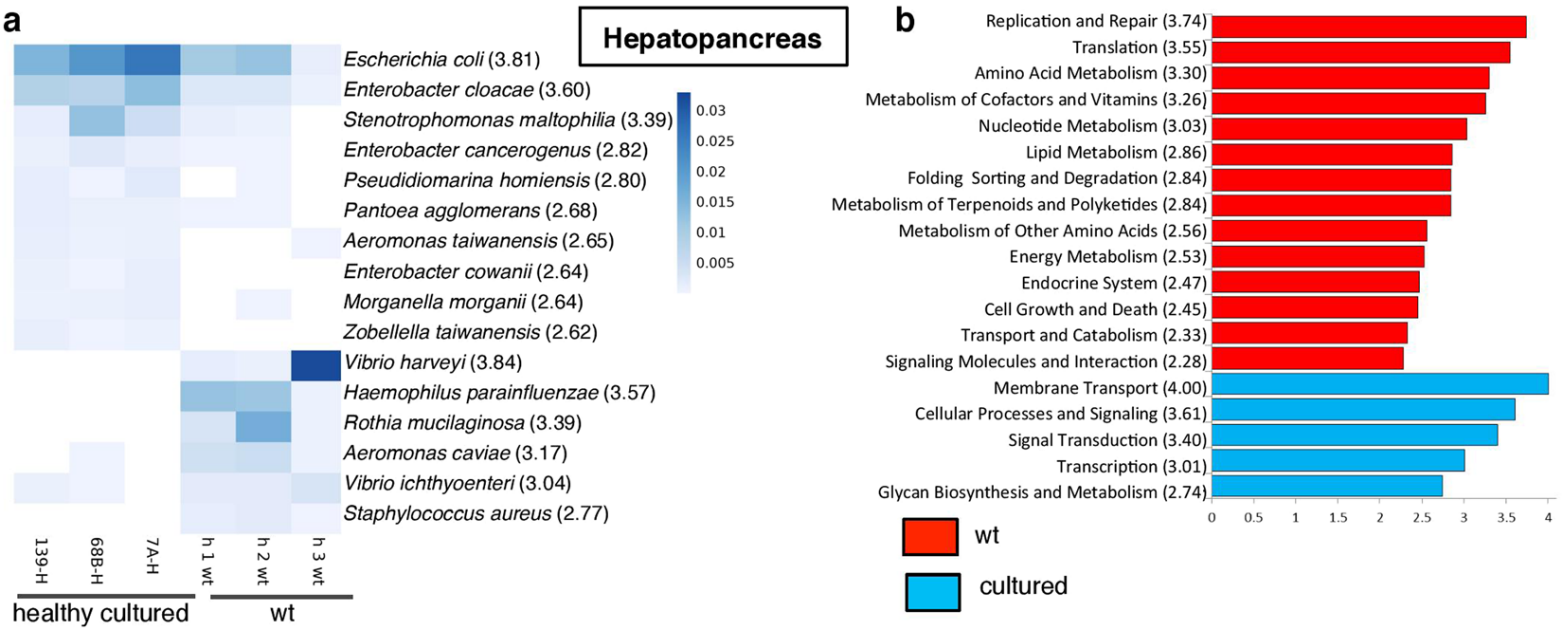
Relative abundance and LEfSe results of enriched microbiota and predicted microbiome functions between wt (n = 3) and cultured hepatopancreas (n = 3) samples. (**a**) Relative abundance of significantly differentially abundant species. Linear discriminant analysis (LDA) score is shown in parenthesis. (**b**) LDA of the differentially abundant KEEG pathways. The differences were significant (*p* < 0.05), among classes (Kruskal-Wallis test) and subclasses (Wilcoxon’s test). The threshold of the logarithmic LDA score was 2.0.

Diversity indices showed that wt and healthy hepatopancreas had significantly (*p* < 0.05) less bacterial diversity than wt and healthy intestines (Fig. 2 and Supplementary Table S4). Hepatopancreas from wt shrimp had significantly (*p* < 0.05) more bacterial diversity than that from healthy cultured shrimp (Fig. 2). *Proteobacteria* was the most abundant phylum (Fig. 1c and Supplementary Table S1), and the *Enterobacteriaceae* family represented 44 and 70% of the total wt and cultured derived sequences, respectively. The *Vibrionaceae*, *Moraxellaceae* and *Pseudomonadaceae* families accounted for 27, 12 and 1%, respectively, of the total reads in wt samples, while these families accounted for 7, 0.5 and 19% of the total reads in cultured samples. At the genus level, *Photobacterium* (16%), *Acinetobacter* (12%) and *Vibrio* (8%) were the most abundant in wt samples, while *Pseudomonas* (18%), *Vibrio* (3%) and *Escherichia* (3%) were the most abundant in cultured samples (Supplementary Table S2). The LDA showed that the phyla *Actinobacteria*, *Firmicutes*, and *TM7* were significantly more abundant in wt samples, while *Proteobacteria*, *Gemmatimonadetes*, and *Spirochaetes* were more abundant in cultured samples (Supplementary Table S9). Classes, orders, families, genera, and species that were differentially abundant between wt and cultured samples are described in Fig. 5a and Supplementary Table S9. The bacterial communities with abundance greater than 0.1% (OTUs > 0.1%) were very different between wt and healthy cultured shrimp (Spearman’s correlation test, r = 0.2036, p = 0.0022). This finding agreed with those observed in the PCoA and UPGMA tree analysis.

The LDA comparison among predicted first level subcategory pathways showed that genes involved in organismal systems and metabolism were significantly enriched in wt shrimp, while environmental and genetic information processing was significantly enriched in cultured shrimp. At level two, there were several enriched pathways, including seven metabolic pathways (metabolism of terpenoids and polyketides, nucleotide metabolism, amino acid and lipid metabolisms, metabolism of other amino acids, metabolism of cofactors and vitamins and energy metabolism) and endocrine system pathways (Fig. 5b) in wt samples. In contrast, five predicted pathways, including three involved in environmental information processing (signal transduction, membrane transport and signaling molecules and interaction), were enriched in cultured samples (Fig. 5b). The higher resolution pathway analysis (level three) of the predicted metagenomes is described in Supplementary Fig. S8b. The NSTI for wt and healthy hepatopancreas was 0.046 ± 0.005 and 0.052 ± 0.003, respectively (Supplementary Table S8).

### Shared microbiota between wt and healthy cultured shrimp

To determine the microbial community shared between wt and cultured shrimp samples, we analyzed the presence/absence of OTUs with a frequency >0.005. This frequency threshold was selected to avoid putative transitory microorganisms in the samples^28^. We considered an OTU to be shared when it appeared in at least one of the three samples from each group. In this regard, we found that 255 OTUs were shared between intestine samples (Supplementary Fig. S9a), while 158 OTUs were shared between hepatopancreas samples (Supplementary Fig. S9b). Additionally, there were unique OTUs that were not shared between wt and cultured samples (Supplementary Fig. S9). These OTUs could represent transient bacterial populations because they could appear in only one of the three samples. Hence, for further analyses, only OTUs that had reads in all of the biological replicates of an organ but did not have reads in any other organ were selected as unique OTUs. In this regard, unique OTUs with taxonomy classifications to the genus and species levels in the intestine and hepatopancreas are shown in Supplementary Table S10. Interestingly, several genera, such as *Faecalibacterium*, *Bacteroides* and *Bifidobacterium*, and species, such as *Coprococcus eutactus* and *Faecalibacterium prausnitzii*, were unique to the intestine from cultured samples (Supplementary Table S10), while the genera *Bacteroides* and *KSA1* and the species *Zobellella taiwanensis* and *Enterobacter cowanii* were unique to the hepatopancreas from cultured samples (Supplementary Table S10). Finally, we analyzed the shared OTUs among the intestines, hepatopancreases, and sediments from cultured samples and observed a shared core composed of 187 OTUs. The majority of OTUs were shared between the sediments and intestines (286 OTUs), followed by 221 OTUs shared between the intestines and hepatopancreases, and 200 OTUs shared between the sediments and hepatopancreases (Supplementary Fig. S9c). The high number of OTUs shared between sediments and intestines suggested strong transfer of bacteria between these two organs.

### Impact of the development of AHPND/EMS outbreak on shrimp microbiome structure and function

The intestinal microbiota plays a major role in the health of the host, contributing to the maintenance of balance against opportunistic pathogens. To investigate the impact of AHPND/EMS disease on the microbiota of the intestine and hepatopancreas, we analyzed samples from cultured shrimp that presented AHPND/EMS symptoms and included pond sediment from where diseased and healthy shrimp were sampled (Fig. 1a). According to the observed OTUs, PD, and Shannon indices, the intestines of diseased shrimp had significantly (*p* < 0.001) higher bacterial diversity than those from healthy shrimp (Fig. 2). In contrast, the diseased hepatopancreas had significantly (*p* < 0.001) lower bacterial diversity (measured by observed OTUs and PD) than the hepatopancreas from healthy shrimp (Fig. 2a and b). Only the Shannon index showed higher bacterial diversity in the diseased hepatopancreas *vs*. that of the healthy shrimp (Fig. 2c). The sediments from ponds with diseased shrimp had significantly (*p* < 0.05) lower bacterial diversity than the ponds containing healthy shrimp (Supplementary Fig. S2).

**Figure 6:**
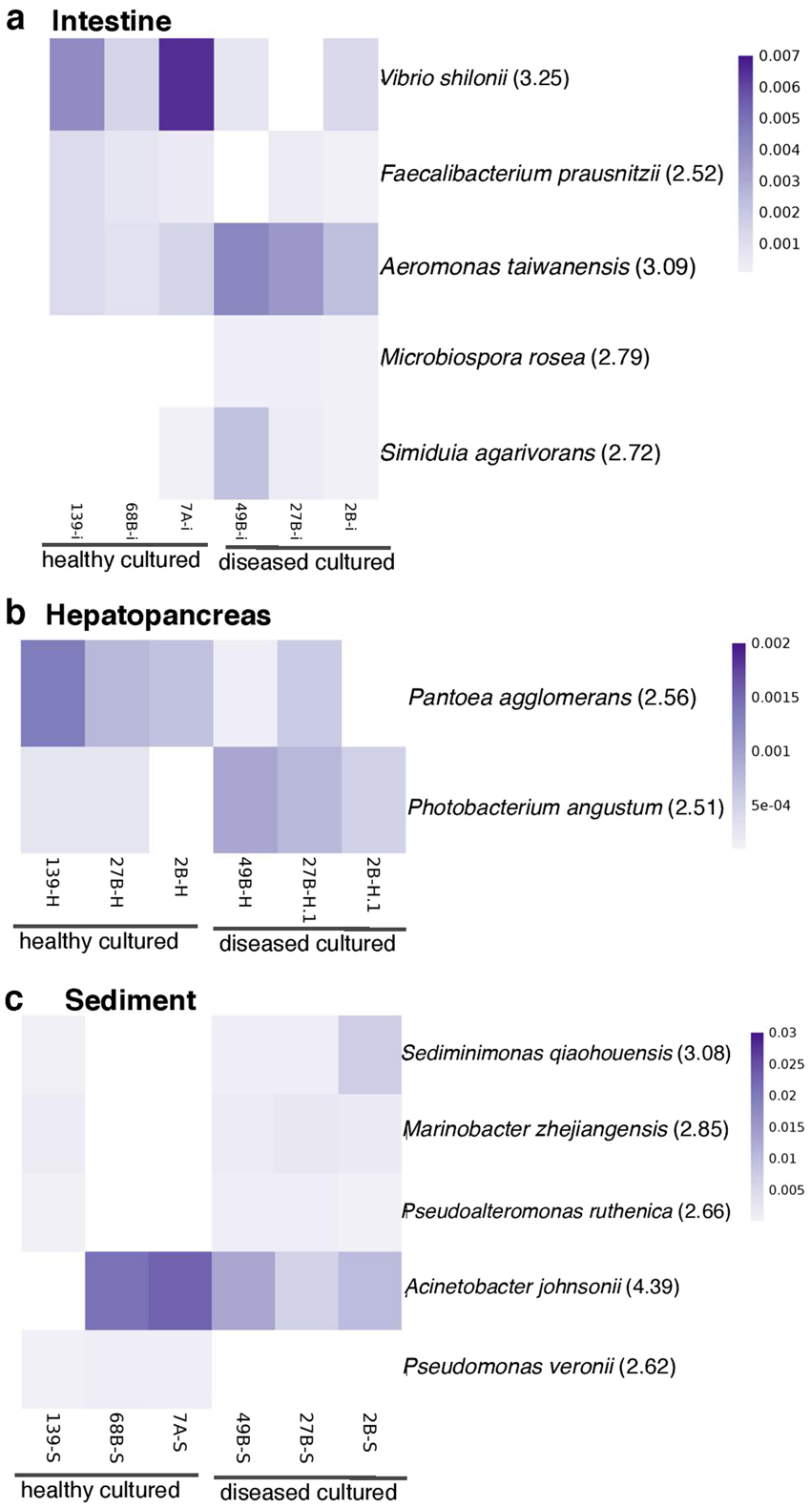
Relative abundance and LEfSe results of the differentially abundant species between healthy (n = 3) and diseased cultured (n = 3) shrimp. (**a**) Intestine, (**b**) hepatopancreas, and (**c**) sediment samples. LDA score is shown in parenthesis. The differences were significant (*p* < 0.05) among classes (Kruskal-Wallis test) and subclasses (Wilcoxon’s test). The threshold of the logarithmic LDA score was 2.0.

PCoA of unweighted and weighted UNIFRAC distances and UPGMA trees with samples tagged as healthy or diseased showed no clustering patterns based on AHPND/EMS symptoms (Supplementary Figs S10 and S11). We found several bacteria differentially enriched between diseased and healthy intestines (Supplementary Table S11) and hepatopancreases (Supplementary Table S12). *Spirochaetes* and *Verrucomicrobia* were enriched in the sediments of diseased samples (Supplementary Table S13). *Vibrio shilonii* and *Faecalibacterium prausnitzii* were enriched in healthy intestine samples, while *Aeromonas taiwanensis*, *Microbispora rosea* and *Simiduia agarivorans* were enriched in diseased intestine samples (Fig. 6a). *Pantoea agglomerans* was enriched in healthy hepatopancreas, while *Photobacterium angustum* was enriched in diseased hepatopancreas (Fig. 6b). The differentially enriched species observed in the sediments are shown in Fig. 6c. The majority of OTUs (198) were shared among the intestine, hepatopancreas, and sediment from the diseased shrimp (Supplementary Fig. S12), the sediment and intestine shared 315 OTUs, followed by the sediment and hepatopancreas, which shared 229 OTUs, and finally the intestine and hepatopancreas, which shared 220 OTUs. *Crenothrix* only appeared in the intestines of healthy organisms, whereas *Microbispora*, *Shinella*, and *Teredinibacter* only appeared in the intestines of diseased samples (Supplementary Table S14). *Escherichia* was only found in the hepatopancreas from diseased samples (Supplementary Table S14). *Pseudomonas* and *Pseudomonas veronii* were only found in the sediment of healthy samples, while *Inquilinus*, *Desulfobacter*, *Salinibacter*, *Flavobacterium*, *Halothiobacillus*, *Acidaminobacter*, *Alishewanella*, and *Salinivibrio* were only found in the sediment of diseased samples (Supplementary Table S14).

There were significant differences in predicted metagenomes between healthy and diseased cultured shrimp. In the first level subcategory, genes involved in genetic information processing were significantly enriched in the hepatopancreas of diseased shrimp. In the level two subcategory, the nucleotide metabolism was enriched, while in the third level subcategory, genes involved in the replication and repair, purine metabolism and valine, leucine and isoleucine biosynthesis, among others, were enriched in the hepatopancreas of diseased shrimp (Supplementary Fig. S13a). In the intestines of diseased shrimp, the amino acid, lipid and carbohydrate metabolism pathways were significantly enriched at the secondary level, while the citrate cycle (TCA cycle) and valine, leucine, and isoleucine degradation were enriched at level three (Supplementary Fig. S13b). In contrast, the intestines of healthy samples were enriched for signal transduction mechanisms and pancreatic secretion pathways in the level three subcategory (Supplementary Fig. S13b). Finally, the metagenomic comparison between the sediments of healthy and diseased shrimp only resulted in significant differences in six pathways in the level three subcategory (Supplementary Fig. S13c). The NSTI for healthy intestines, hepatopancreases and sediments was 0.112 ± 0.072, 0.052 ± 0.003 and 0.142 ± 0.027, respectively (Supplementary Table S8). The NSTI for diseased intestines, hepatopancreases and sediments was 0.1 ± 0.007, 0.050 ± 0.003 and 0.132 ± 0.029, respectively (Supplementary Table S8).

The results from the AP3 method for amplification of the ToxA gene to detect AHPND/EMS were negative for all intestine and hepatopancreas organs from wt samples (Supplementary Table S15). Thus, the wt shrimp did not have the AHPND/EMS-associated plasmid. All of the hepatopancreases from cultured samples were positive for the presence of the plasmid. In contrast, two intestines from cultured healthy samples and one intestine from cultured diseased samples yielded negative results, while one intestine from cultured healthy samples and two intestines from cultured diseased samples yielded positive results. This finding is expected in early stages of AHPND/EMS since tissues with higher bacterial density during the disease are the hepatopancreas and stomach^29^.

## Discussion

The Pacific whiteleg shrimp *Litopenaeus vannamei* is the most economically important shrimp species worldwide. In this work, we characterized the microbiota and predicted its metagenomic functions in hepatopancreas and intestine samples from natural environment and healthy and diseased aquacultured *L*. *vannamei* shrimp. Since soil is a fundamental component of the shrimp microbiome, we also included pond sediment from the shrimp hatcheries. The sequencing of seven hypervariable regions of the 16S rRNA gene, using the Ion 16S™ Metagenomics Kit, has been previously reported for other models^30,31^, suggesting that more than one hypervariable region is needed to provide reliable diversity inferences^30^. Previous studies in shrimp microbiota only targeted two hypervariable regions (typically V1-V3 or V3-V4)^6,8,11,14,16^. Here, we reported the first shrimp microbiota characterization incorporating the sequencing of seven hypervariable regions using the Ion 16S™ Metagenomics Kit, while avoiding the inherent diversity bias of selecting only one or two hypervariable regions of the gene^2,31^. However, it is important to note that we also found several OTUs that were classified using only one of the hypervariable regions (Supplementary Fig. S3), suggesting: (i) that different hypervariable regions of the same ribosomal gene might be assigned to different taxonomies; or (ii) that there are ribosomal genes that can only be classified using one hypervariable region. Similar behavior has been observed when several hypervariable regions were used to classify microbiota in other studies^31,32^. To understand the consequences of these biases in microbiota analysis, the Ion 16S™ Metagenomics Kit should be further tested using well-characterized control samples containing known bacterial strains in well-quantified proportions.

Considering that we compared non-overlapping amplicons from seven hypervariable regions (V2, V3, V4, V6-V7, V8 and V9) of the 16S rRNA gene and that this comparison did not allow for the identification of novel organisms in a *de novo* OTU picking process, we could only detect the microorganisms reported in the GreenGenes database. Therefore, there is still an opportunity to investigate further the role that new bacterial diversity plays in microbiota composition of this model. Differences in read abundance have also been observed for some hypervariable regions using this kit^30,31^, and they were also observed in our study (Supplementary Fig. S2). Interestingly, the differences were associated with the sample origin (i.e. wt or cultured) and the analyzed organ (i.e. hepatopancreas or intestine). These differences could suggest distinct abundances of specific microorganisms, but they could be normalized using the sequences from all seven of the hypervariable regions. Using different hypervariable regions or the sequencing errors, such as those arising from pyrosequencing and single molecule sequencers (Roche 454 and PacBio), can lead to artificial inflation of diversity estimates^33,34^. It is well known that Ion Torrent data exhibit a higher rate of sequencing errors, compared to data from the Illumina platform^7–9,35–37^. However, a recent study using Illumina MiSeq and the Ion Torrent for bacterial community profiling by 16S rRNA amplicon sequencing showed that the absolute difference in error rates between the two platforms was not significant^35^. Our rarefaction curves did not reach the saturation even after 16,000 reads (Supplementary Fig. S5b). Although, the Good’s coverage revealed that we obtained, on average, ~80% of the total OTUs at sequencing depth of 2,078 reads (Supplementary Table S5), meaning that significant more sequencing depth is necessary to discover the total OTUs in the analyzed microbiomes. These data suggested that further investigations using well-characterized control samples are needed to quantify the real impact that the combination of different hypervariable regions could have in the overestimation of the diversity.

In general, we found significantly less diversity in the hepatopancreas than in the intestine, independent of shrimp origin (wt or cultured), in contrast to that previously estimated in *N*. *denticulata* ^16^, in which the authors reported that the hepatopancreas has more bacterial diversity than the intestine. The controlled laboratory conditions used in the *N*. *denticulata* study could explain such differences since, in this work, the wt samples were collected in the open sea, and cultured specimens were obtained under commercial hatchery conditions. *Proteobacteria* represented ~76, ~95 and ~50% of all reads from the intestine, hepatopancreas and sediment samples, respectively. The predominance of *Proteobacteria* in shrimp has been previously observed, but only in shrimp cultured under laboratory conditions^6,8,11,14,16^. The similarity found between the intestine and sediment microbiota from cultured shrimp was consistent with well-described burrowing behavior, in which shrimp spend long periods of time buried in the sediment. In this regard, Xing *et al*.^38^ suggested several pathways explaining how the gastrointestinal metagenome of the farmed adult turbot originates from the sediment^38^. Therefore, knowledge-based management of sediment could lead to a modification of the shrimp microbiota and ecologically sound strategies to promote the health status of shrimp hatcheries.

The OTUs shared between wt and cultured shrimp could be bacteria selected by the host physiology and genetics, while the unique OTUs in the wt or the cultured samples might be influenced by the environment (natural *vs*. cultured) and by feeding conditions (natural detritus and microalgae *vs*. formulated diets). The PCoA and UPGMA analyses showed that the wt microbiota is different from the cultured microbiota and that microbiota from the same organ is more similar across shrimps from the same origin (wt or cultured) than microbiota from different organs of the same shrimp, consistent with the observations in higher animals, such as human, koala, and others^39,40^. These results also indicated that the environment has a greater influence on bacterial diversity than the shrimp organ. However, in the UPGMA tree from the wt samples, one hepatopancreas clustered with intestine samples, and the same phenomenon was observed for another intestine that clustered with hepatopancreas samples (Fig. 3c). The bacterial profiles of the OTUs greater than 0.1% (OTUs > 0.1%) showed low Spearman’s correlations for these two samples among their replicates (Supplementary Table S3). Additionally, the OTU correlations (OTUs > 0.1%) among wt triplicates were low and ranged from 0.38 to 0.87, while the correlations among cultured triplicates were high and stable, ranging from 0.77 to 0.94 (Supplementary Table S3). That wt samples had lower correlations between replicates could suggest that feed heterogeneity under natural conditions might have an impact on microbiota composition, compared to the stable correlations observed in cultured shrimp living under controlled feed conditions. There is a general notion that animals in their natural environments have a richer microbiota^41^. In this regard, the intestine of wt tiger shrimp *P*. *monodon* has more bacterial diversity than that of shrimp from hatcheries^11^. In agreement with this general hypothesis, our domesticated shrimp reared under controlled conditions with formulated diets and water management systems have lower bacterial diversity than wt organisms. These controlled conditions are also reflected in the higher and similar OTU correlations among our triplicates from cultured contrary to the ones observed in wt samples (Supplementary Table S3), also suggesting the homogeneity of the shrimp microbiota under cultured conditions.

Recently, Xiong *et al*.^24^ reported that shrimp development from nauplii to adult organisms can explain only 9.7% of the variation of gut bacterial communities in *L*. *vannamei*. Moreover, they identified 28 age discriminatory taxa for shrimp. We found 7 of these 28 taxa in our microbiota data (Materials and methods). Because the shrimp sampled for this work had different sizes (~44 g in wt and ~16 g in cultured) and therefore probably different adult ages, we conducted a β-diversity analysis among all samples, eliminating these 7 age-discriminatory taxa (Supplementary Table S16). We observed that community clustering was very similar to that observed including these taxa associated with shrimp age (Supplementary Fig. S14), suggesting that a difference in shrimp age did not cluster the microbiota in our samples.

Coupled with the microbiota characterization of all of the shrimp samples, we used the 16S sequencing data to predict the metagenome functions. The accuracy of these predictions using PICRUSt depends on how closely related the microorganisms in the studied sample are to microorganisms with already sequenced genomes. This accuracy is measured by NSTI value, with lower NSTI values indicating a closer relationship with sequenced genomes. As expected, the NSTI values were greater for the phylogenetically most diverse sediments (0.137 ± 0.026), midrange for the intestines (0.098 ± 0.043), and smaller for the hepatopancreas (0.049 ± 0.004). For comparison, in the study by Langille *et al*.^27^, they found that mammalian intestines had an NSTI = 0.14, and soil samples had a NSTI = 0.17, while well-covered human microbiome samples had an NSTI = 0.03. Thus, our shrimp and sediment samples fit with the NSTI described in other microbiomes^27,42,43^.

The hepatopancreas is the major digestive gland in invertebrates, and it integrates many digestive, metabolic and immune functions^17^. The pathogen clearance and antigen processing system^44^ contain molecules related to the innate immunity, such as lectins, hemocyanin, ferritin, antiviral and antibacterial peptides and proteins, proteolytic enzymes and nitric oxide^17^. The lower diversity observed in the hepatopancreas suggests that the innate immunity restricts the natural microbiota to bacteria that do not trigger the host protective mechanisms against pathogens. Interestingly, this microbiota appears to be conserved across species since such immune mechanisms have been reported to occur in the mouse intestine^45^. Additionally, it is known that short-chain fatty acids (SCFAs) and tryptophan metabolites play major roles in adaptive immune cell homeostasis in several organisms^46^. Consistently, we found predicted genes that were enriched for butyrate and propionate metabolism and for tryptophan biosynthesis in the microbiota of the wt hepatopancreas, which could modulate bacterial diversity and play a protective role. We also found several pathways, such as the metabolism of amino acids, cofactors, vitamins, nucleotides, lipids, terpenoids, energy, and the endocrine system, in addition to signaling molecules, that were enriched in the wt hepatopancreas.

Pathways for the degradation of branched amino acids (valine, leucine, and isoleucine) were significantly enriched in the hepatopancreas and intestine from wt shrimp. These amino acids are important constituents of hemocyanin, the respiratory protein in crustaceans and mollusks^47^, which also has a function in the immune response^48^. All of the aforementioned microbiota-derived functions were not found in healthy cultured shrimp. The current shrimp lineages used in commercial hatcheries have been selected by traditional genetic selection methods over decades for better food conversion rates, growth and disease resistance, which might have also changed the natural microbiota^49^. These changes have been much less studied and understood in shrimp because critical physiological differences exist between vertebrate fish and invertebrate shrimp. Additionally, loss of bacterial diversity might favor the occurrence of opportunistic diseases^15^. Similarly, it was observed in non-human primates kept in captivity that an association existed between loss of natural intestine microbiota with convergence towards the human microbiota^41^.

The composition of the diet modulates the microbiota^49^. Complex carbohydrates are critical because of their role as a prebiotic substrate for the growth of bacteria and for their role in signaling. Invertebrates have general mechanisms for detecting specific structures, such as beta-glucans, and for triggering innate immune responses. Glycans are typically present in commercial shrimp feed used in aquaculture hatcheries, and they are an important factor that shapes the microbiota composition of many species, including humans^50^. Interestingly, the predicted genes for glycan biosynthesis and metabolism were enriched in the intestine and hepatopancreas from healthy cultured shrimp (Supplementary Fig. S8), suggesting that microbiota of the intestine and hepatopancreas could respond to glycan dietary changes, allowing for the manipulation of bacterial communities to promote host health by adding natural glycan prebiotics to traditional shrimp diets. Additionally, the increased xenobiotics metabolism observed in the microbiota of the wt intestine points toward the management of environmental marine contamination.

Regarding disease, there is not a clear line that establishes the early stages of AHPND/EMS. The diagnosis is made based on PCR assay detecting the plasmid encoding Pir A/B toxins. Production of the toxins is quorum sensing dependent^29^, and until today, there are no methods to directly detect the presence of Pir A/B proteins. In all of the wt samples, the AP3-PCR results were negative, indicating that the plasmid was absent in these samples. The AP3 results for cultured shrimps were controversial because the AP3-PCR was positive in 4 of 6 healthy samples and in 5 of 6 diseased samples (Supplementary Table S15). However, one nonpathogenic *V*. *parahaemolyticus* strain has also previously yielded positive results for AP3 diagnosis^29^. Nonetheless, none of the healthy cultured shrimp showed symptoms of AHPND/EMS (Supplementary Fig. S15). Moreover, organisms from the diseased ponds presented the symptoms and mortality of the disease, including lethargy, empty intestine and pale and white aqueous hepatopancreas (Supplementary Fig. S15). These results suggested that the presence of the plasmid-encoding Pir A/B toxins requires additional triggering factors to lead to AHPND/EMS disease. As mentioned, quorum sensing is known to be a trigger factor for AHPND/EMS since it has been reported that the threshold infective density is 10^4^ CFU ml^−1^, and less than that bacterial density, no mortality occurred^29^. We also observed a non-significant difference in the bacterial community clustering between healthy and diseased samples (Fig. 3). Interestingly, this finding was in agreement with a recent study in which there were no significant changes in bacterial community clustering or diversity in the early stages of a disease in *L*. *vannamei* ^24^.

In general and across species, loss of diversity is associated with diseases, as well demonstrated in humans^2^. We found that AHPND/EMS was associated with a significant loss of hepatopancreas microbiota diversity, compared to healthy shrimp (*p* < 0.01) measured by PD and the number of OTUs (Fig. 2), although AHPND/EMS was associated with a bacterial toxin and not with a bacterial infection *per se* ^18^. An opposite effect was observed in the diseased intestine, where the microbiota diversity significantly increased (*p* < 0.01) as a consequence of AHPND/EMS disease (Fig. 2). Notably, a limitation of our study was the small sample size (n = 3) and low sequence depth, making it necessary to study more samples in future studies to confirm these changes in bacterial diversity. Nevertheless, our analysis leads to the notion that some bacteria are lost during shrimp domestication (wt *vs*. cultured), which could result in the need to produce probiotic strains to be administered in cultured organisms to promote shrimp health. In this regard, we found some bacteria enriched in either wt or healthy samples, suggesting their use as probiotics for *L*. *vannamei*. First, some members of the *Janthinobacterium*, *Dietzia*, *Streptomyces* and *Bacillus* genera, which produced antibiotics and antifungal molecules^51,52^ were only found in the intestines of wt shrimp. Additionally, only in these samples was *Pseudomonas stutzeri* present, which has been reported as a probiotic that protects against pathogenic *Vibrio* in Artemia^53^ and improves the growth, survival, and immunity of *P*. *monodon* ^54^. Furthermore, several species of the genus *Micrococcus* are known to have probiotic potential by inhibiting intestinal colonization by pathogens, as found in juvenile *L*. *vannamei* ^55^ and *Macrobrachium rosenbergii* larvae^56^. Interestingly, the genus *Microccocus* was only present in the hepatopancreas of wt shrimp.

In contrast, bacteria from the *Faecalibacterium* and *Bifidobacterium* genera, which are typically used as probiotics in humans^57,58^, were enriched in the intestines of healthy cultured shrimp. Specifically, *Faecalibacterium prausnitzii* was only present and significantly enriched in the intestines of healthy cultured shrimp, compared to the same organs in diseased shrimp. This butyrate-producing bacteria is the most abundant in the intestinal microbiota of healthy adults, in which changes in its abundance have been linked to several human disorders^59,60^. Accordingly, our results suggested that this bacterium might have beneficial effects on shrimp health and could be used as a probiotic for *L*. *vannamei* affected by AHPND/EMS. Furthermore, *Pantoea agglomerans* was enriched in the hepatopancreas of healthy versus diseased cultured shrimp. The oral administration of lipopolysaccharide (LPS) derived from this bacteria has been reported to enhance disease resistance against penaeid acute viremia in the hemolymph of the kuruma shrimp, *Penaeus japonicus* ^61^. Additionally, LPS from other bacteria has been previously used as an ‘immunostimulant’ in *L*. *vannamei* but mainly against viral infections^62^. Our results suggest the idea that *P*. *agglomerans* might be used as a potential probiotic against AHPND/EMS disease in *L*. *vannamei*. The genus *Aeromonas* is an important disease-causing pathogen in fish, and it is responsible for infectious complications in both immunocompetent and immunocompromised humans^63^. Here, we report enrichment of *Aeromonas taiwanensis* in the intestine of diseased shrimp. This bacterium has been reported in aquatic environments worldwide and causing human diseases^64^, but it has not been reported in shrimp. *Simiduia agarivorans* is a marine bacterium that can degrade a variety of complex polysaccharides, such as chitin^65^, a biopolymer that is abundant in aquatic habitats (e.g., from shrimp exoskeletons). Moreover, it has been demonstrated that *V*. *parahemolyticus* can adsorb and multiply on chitin and its soluble derivatives^66^. Hence, the enrichment of *S*. *agarivorans* in the intestines of diseased shrimp could be related to the presence of *V*. *parahemolyticus* in diseased shrimp.

Other interesting metabolic pathways were the biosynthesis and degradation of branched amino acids known to increase during infection processes in other organisms, including humans^67^. Interestingly, these pathways were enriched in the diseased hepatopancreas and intestine microbiomes, which might have been associated with the APHND/EMS disease process. Species of *Photobacterium* are ubiquitous in seawater, marine organisms, marine sediments and saline lakes^68^. *P*. *damselae* is considered a bacterial pathogen of humans and many aquatic organisms, including fish, mollusks, and crustaceans^68^. Recently, several strains of *P*. *damselae* causing mortality of *Exopalaemon carnicauda*, *P*. *monodon* and *L*. *vannamei* shrimp have been reported^69,70^. Interestingly, the symptoms of shrimp affected by these bacteria were very similar to symptoms observed in the shrimp hepatopancreas with AHPND/EMS caused by *V*. *parahaemolyticus* ^69^. In our study, *P*. *angustum* was enriched in the hepatopancreas of diseased shrimp (Fig. 6b), and although there have not been any reports that this bacterium is a pathogen for shrimp, our results emphasize that further studies will clarify its role in the development of the AHPND/EMS disease.

Finally, we found some bacteria enriched in the pond sediment. First, *Acinetobacter johnsonii*, which reduces phosphorus in the water by 10–30% and releases inorganic phosphate under anaerobic conditions, was enriched in the sediment from healthy shrimp. Also enriched in these samples was *Pseudomonas veronii*, which has been used for bioremediation from contaminated soils with aromatic organic compounds^71^. These two bacteria could potentially be used to treat AHPND/EMS disease. In contrast, *Pseudoalteromonas ruthenica*, an exopolymer-producing biofilm bacterium, was enriched in diseased sediment. This bacterium has been isolated in the seawater at the entrance of a tropical power station^72^. However, more investigations will be needed to analyze the roles of these enriched species in healthy and diseased shrimp and their sediment to fully understand their roles in AHPND/EMS disease.

## Materials and Methods

### Ethics statement

An ethics statement was not required for this work, and no specific permissions were required for the described study. The specimen collection location is not privately owned or protected in any way, and the field studies did not involve endangered or protected species. Animals were sacrificed under university protocols to avoid animal suffering.

### Sample collection

All of the specimens were identified as *Litopenaeus vannamei* by morphological keys^73^. Wt shrimp (wt, n = 3, average weight = 43.8 ± 2.1 g) (Supplementary Table S17) were obtained from the Mexican Pacific area 5 km from Nayarit’s state coast at 21.4986° latitude, −105.3169° longitude (Fig. 1a), using a traditional shrimp-trawling system at 15 m of depth (salinity = 32 ppm and temperature = 24 °C). Aquacultured shrimp were collected from a shrimp hatchery located 1,000 km from the site of the wt shrimp collection (Sonora state, 28.4030° latitude and −111.4513° longitude, Fig. 1a). The diseased cultured shrimp (n = 3, average weight = 15.3 ± 0.4 g) (Supplementary Table S17) were obtained from a pond in which the shrimp presented mortality and had the usual clinical and pathological symptoms of the disease, such as lethargy, empty intestine and pale and white aqueous hepatopancreas (Supplementary Fig. S15). The three selected shrimp showed clear symptoms of the disease (Supplementary Fig. S15). The healthy cultured shrimp (n = 3, average weight = 17.1 ± 1.2 g) (Supplementary Table S17) were obtained from a pond without the AHPND/EMS disease symptomatology. After a one-way ANOVA test, there were no trends among the shrimp of different weight within the same group (Supplementary Table S17). Cultured shrimp were fed three times per day using commercial feed (~35% protein) for three months (water salinity ~40 ppm and temperature ~29 °C). Shrimp hepatopancreases and intestines were aseptically dissected *in situ*. The tissues were kept in RNA-later solution for 24 h (~4 °C) and were stored at −80 °C until used. Additionally, three sediment samples (50 g) were collected from each pond using a sediment core (3.8 cm diameter by 10 cm depth). The samples were homogenized and immediately stored in liquid nitrogen. All of the samples were individually screened by the diagnostic PCR method for AHPND/EMS^74^. The samples from cultured shrimp that were positive for the AP3 test were labeled EMS+, and samples that were negative were identified as EMS- (Supplementary Table S15).

### Sample DNA extraction, library preparation, and sequencing

Total DNA was extracted from all of the samples using the ZR Soil Microbe DNA MicroPrep (Zymo Research, Irvine, CA, USA). DNA concentration was determined using Qubit fluorometer (Life Technologies, Carlsbad, CA, USA), and DNA integrity was confirmed by agarose gel electrophoresis. The amplicons were generated as described in the Ion 16S™ Metagenomics Kit (Thermo Fisher, Waltham, MA, USA) user’s guide with some modifications. Briefly, the amplicons were prepared using 200 ng of total DNA from the intestine and hepatopancreas samples and 300 ng of total DNA from sediment samples. Two reactions were performed for each sample, one for the V2-4-8 (V2 mix) primer set and another for the V3-6-7-9 (V3 mix) primer set. All of the reactions were adjusted to a final volume of 20 µl and amplified for 35 cycles following the manufacturer’s thermal cycler conditions. After amplification, each reaction was run on an electrophoresis agarose gel and, in every case, observing 200–300 bp bands. All of the amplicons were purified directly from the agarose gel using the QIAquick Gel Extraction Kit (Qiagen, Venlo, The Netherlands) and were quantified using the Qubit fluorometer. Next, based on the quantifications, equimolar quantities of V2 and V3 were mixed in each sample, and the mixture was then used to prepare the Ion Torrent sequencing libraries, as described in the User Guide of the Ion 16S™ Metagenomics Kit. The quantity and size distribution of each library were assessed using the Qubit fluorometer and the Agilent 2100 Bioanalyzer (Agilent Technologies, Santa Clara, CA, USA). Finally, all of the libraries were sequenced using Ion PGM™ Sequencing 400 technology in two Ion 316™ v2 chips (Thermo Fisher, Waltham, MA, USA).

### Bioinformatics and statistical analysis

Sequencing reads were generated using the 400-base read length chemistry of the Ion PGM System, and after quality filtering (≥Q25) and ambiguous base removal, 1,060,822 reads were obtained. After read trimming (≥120 bp), 305,209 reads were retained and analyzed using QIIME^75^ (version 1.8). These sequences were clustered using UCLUST into operational taxonomic units (OTUs), based on 97% sequence similarity through pick_closed_reference_otus.py command against the Green Genes reference sequence collection (version 13_5). The option of reverse strand matching was enabled, and any reads that did not hit a sequence in the reference sequence collection were excluded from downstream analyses. To assign taxonomy to the OTUs, we provided taxonomic assignments for the Green Genes reference database. We selected the closed-reference OTU picking command because we were comparing non-overlapping amplicons. This OTU picking method was a reference-based approach. Thus, chimera removal was not necessary. A total of 153,361 high-quality filtered reads with a 215 bp mean read length were retrieved for the 24 samples with sequence numbers ranging from 1,032 to 15,814 (Supplementary Table S4). The OTUs represented by a single read (singleton) were discarded from further analyses, which helped to keep the estimates of α-diversity realistic and to avoid information loss. Taxonomy summaries with relative abundance data were subsequently generated using the summarize_taxa_through_plots.py command. The most abundant sequence within an OTU was selected as the OTU’s representative, and its sequence was obtained using the pick_rep_set.py command. The representative sequences were then aligned against Green Genes using the align_seqs.py command and PyNAST with a minimum sequence identity of 75%. The alignment was filtered using filer_alignment.py, and a phylogenic tree was constructed using the make_phylogeny.py command with the FastTree method for tree building.

Alpha and beta diversity metrics from the final OTU table without singletons were obtained using the QIIME^75^ alpha_rarefaction.py and beta_diversity_through_plots.py commands, respectively. The rarefaction curves until the deepest library (15,000 reads) are shown in Fig. S5b. To increase the sequence depth, we discarded two samples with low sequencing depth (68B-S and 139-I). Thus, the alpha diversity metrics were calculated at sequence depth of 2,078 reads per sample with 10,000 iterations and then were averaged. The selected maximum sampling depth corresponded to the minimum number of reads obtained from any of the remaining sequenced samples. Beta diversity was estimated by computing from the phylogenetic tree the weighted and unweighted UniFrac distances among samples at a sequence depth of 1,032 reads per sample, and the UniFrac distance matrices were visualized using PCoA analysis. The sequence depth threshold of 1,032 reads was chosen to include all samples for the beta diversity analysis. The robustness of the UPGMA tree was estimated with jackknife, based on 1,000 replicates and using the beta_diversity.py, multiple_rarefactions_even_depth.py, upgma_cluster.py and tree_compare.py commands. To determine the taxonomic classifications that were significantly more abundant in the cultured and wt samples, we applied a Wilcoxon’s non-parametric rank-sum test, followed by LDA using the LEfSe^26^ program. The alpha diversity comparisons were evaluated using the Mann-Whitney test (nonparametric t-test) using a 95% level of confidence (*p* < 0.05). The final OTU table was also used as an input for functional metagenomic prediction using PICRUSt^27^. The KEGG pathway content obtained by PICRUSt was normalized and then used to obtain the metagenomic functional predictions at different hierarchical KEGG levels (1, 2 and 3). Additionally, we used the –a option in the predict_metagenomes.py command of PICRUSt to calculate the NSTI value for the functional predictions in each sample.

The OTU’s and predicted pathways were subjected to a differential abundance analysis with LEfSe to obtain the OTUs and pathways that were significantly different between samples. A significance level (alpha) of 0.05 was used, and the LDA threshold >2 was used to determine significantly abundant OTUs and pathways. We then applied a false discovery rate (FDR) test to prioritize the most significant enrichment results of LEfSe, however, no significant differences between all the groups were detected, likely due to the small number of replicates for each group (n = 3)^76,77^.

The 28 age discriminatory taxa were reported by Xiong *et al*.^24^ in a *de novo* analysis. Thus, to compare it with our study, which used a closed analysis, the taxa were blasted against the GreenGenes database (version 13_5), to identify the corresponding ribosomal genes in our database. Subsequently, we only found 21 OTUs at ≥97% sequence identity, from which only 7 OTUs had read abundance in our data (Supplementary Table S16). Hence, the β-diversity analysis between all samples was conducted, eliminating these age-discriminating OTUs from our original biom table.

### Separating reads into different hypervariable regions

The 16S metagenomics kit from Ion Torrent contains six proprietary primer pairs designed to amplify seven different hypervariable regions (V2, V4 and V8 in one PCR reaction and V3, V6-7, and V9 in a second PCR reaction). Since the primer sequences were not published by the company, we required a reference of ribosomal genes for alignment. To this end, we divided the full-length aligned 16S rRNA sequences of GreenGenes reference (version 13_5), using the breakpoints previously reported for the V2, V3, V4, V6- V7, V8 and V9 regions^78^. These sub-regions were divided into 6 files that were used as a 16S rRNA reference for each hypervariable region. The previous 153,361 reads with assigned taxonomies were clustered using UCLUST into OTUs, based on 97% sequence similarity, using the pick_closed_reference_otus.py command against each of the six archives of hypervariable regions. The option of reverse strand matching was enabled, and the pipeline was run 6 separate times to obtain OTU tables for each hypervariable region. The taxonomy was assigned using the assign_taxonomy.py command in QIIME, and the relative abundances were obtained for each hypervariable region and for each sequenced sample.

### Data Availability

Amplicon sequencing datasets are available in the NCBI SRA database under the following accession numbers: SRR5585664, SRR5585665, SRR5585666, SRR5585667, SRR5585668, SRR5585669, SRR5585670, SRR5585671, SRR5585672, SRR5585673, SRR5585674, SRR5585675, SRR5585676, SRR5585677, SRR5585678, SRR5585679, SRR5585680, SRR5585681, SRR5585682, SRR5585683, SRR5585684, SRR5585685, SRR5585686, and SRR5585687; and under the NCBI Bioproject PRJNA387510.

## Electronic supplementary material

supplementary information
