# Supplementary material for: Microbiome of Pacific Whiteleg shrimp reveals differential bacterial community composition between Wild, Aquacultured and AHPND/EMS outbreak conditions
Source: Sci Rep. 2017 Sep 18;7:11783. doi: 10.1038/s41598-017-11805-w (PMC5603525; doi:10.1038/s41598-017-11805-w)
Supplement: Supplementary file 1 — supplementary information [file 41598_2017_11805_MOESM1_ESM.pdf]

# Microbiome of Pacific Whiteleg shrimp reveals differential bacterial community composition between wild, aquacultured and AHPND/EMS outbreak conditions

Fernanda Cornejo-Granados, Alonso A. Lopez-Zavala, Luigui Gallardo-Becerra, Alfredo Mendoza-Vargas, Filiberto Sánchez, , Rodrigo Vichido, Luis G. Brieba, Maria Teresa Viana, Rogerio R. Sotelo-Mundo and Adrián Ochoa-Leyva

**Supplementary Fig. S1** Reads percentage distribution in each 16S rRNA sequenced hypervirable region for all samples

**Supplementary Fig. S2** Reads percentage coverage across all sequenced 16S rRNA hypervirable regions for all groups.

**Supplementary Fig. S3** Venn diagrams between assigned OTUs for every hypervariable region for a) intestine, b) hepatopancreas, c) pond sediment.

**Supplementary Fig. S4** Percentage of unknown OTU reported in GreenGenes database. a) Percentage of unknown reads for all the sequenced samples. b) Average percentage of unknown reads and standard deviation barrs for all the sequenced groups.

**Supplementary Fig. S5** Alpha diversity rarefaction curves. a) Shannon index until the library with the most reads. b) Obseved OTUs for all the 24 samples. c) Obseved OTUs for all the sequenced samples grouped by treatment.

**Supplementary Fig. S6** PCoA using weighted UniFrac distances. a) tagged as wild type and cultured and b) samples tagged by shrimp organ or sediment.

**Supplementary Fig. S7** UPGMA tree of weighted UniFrac distances. Node value represents the jackknife support using 1,000 replicates.

**Supplementary Fig. S8** LDA of the KEEG pathways at the level three between wild type and cultured samples. a) intestine and b) hepatopancreas samples.

**Supplementary Fig. S9.** Venn diagrams showing the shared OTUs. a) wt vs cultured healthy shrimp intestine. b) wt vs cultured healthy shrimp hepatopancreas. c) healthy intestine vs hepatopancreas vs pond sediment.

**Supplementary Fig. S10** PCoA of UniFrac distances. a) unweighted and b) weighted. The samples are tagged as healthy and diseased.

**Supplementary Fig. S11** UPGMA tree of a) unweighted and b) weighted UniFrac distances. The blue box highlights the diseased samples. Node value represents the jackknife support using 1,000 replicates.

**Supplementary Fig. S12** Venn diagrams showing shared OTUs between healthy and diseased samples. a) healthy vs diseased intestine. b) diseased intestine vs diseased hepatopancreas vs diseased pond sediment. c) helthy vs diseased hepatopancreas. d) healthy vs diseased pond sediment.

**Supplementary Fig. S13** LDA of the KEEG pathways at the level three between healthy and diseased cultured samples. a) hepatopancreas, b) intestine and c) pond sediment samples.

**Supplementary Fig. S14** PCoA using unweighted UniFrac distances without 7 age discriminatory OTUs. a) tagged as wild type and cultured and b) tagged by shrimp organ or sediment

**Supplementary Fig. S15** Photographs of a) healthy cultured shrimp. b) diseased cultured shrimp. The diseased shrimps presented the phenotypic characteristics of the APHND/EMS disease such as: lethargy, empty intestine and pale and white aqueous hepatopancreas. c) wild type shrimp.

**Supplementary Table S1.** Relative abundance of OTUs with an abundance >0.1% at phylum level.

**Supplementary Table S2**. Average relative abundance of top 5 OTUs with an abundance >0.1% at family and genus level.

**Supplementary Table S3.** Spearman correlation test between group triplicates.

**Supplementary Table S4.** Number of reads, observed OTUs and alpha diversity indices for all the sequenced samples.

**Supplementary Table S5.** Good's Coverage of sequenced samples (10,000 iterations at 2,078 sequence depth).

**Supplementary Table S6.** Linear Discriminant Analysis of enriched taxonomies in wt and cultured intestine samples using LEfSe.

**Supplementary Table S7.** Relative abundance of KEGG pathways obtained by PICRUSt analysis.

**Supplementary Table S8.** NSTI mean score for all the sequenced groups.

**Supplementary Table S9.** Linear Discriminant Analysis of enriched taxonomies in wt and cultured hepatopancreas samples using LEfSe.

**Supplementary Table S10**. Relative frequency of unique genera and species in sequenced wt and cultured samples.

**Supplementary Table S11**. Linear Discriminant Analysis of enriched taxonomies in cultured healthy and diseased intestine using LefSe.

**Supplementary Table S12**. Linear Discriminant Analysis of enriched taxonomies in cultured healthy and diseased hepatopancreas using LefSe.

**Supplementary Table S13**. Linear Discriminant Analysis of enriched taxonomies in cultured healthy and diseased sediment using LefSe.

**Supplementary Table S14**. Relative frequency of unique genera and species in sequenced healthy and diseased samples.

**Supplementary Table S15**. AP3 PCR diagnostic results.

**Supplementary Table S16**. Relative abundance of 7 age discriminating OTUs present in the sequenced samples.

**Supplementary Table S17**. Weight of the wt and cultured shrimps used for the extraction of sequenced intestines and hepatopancreas.

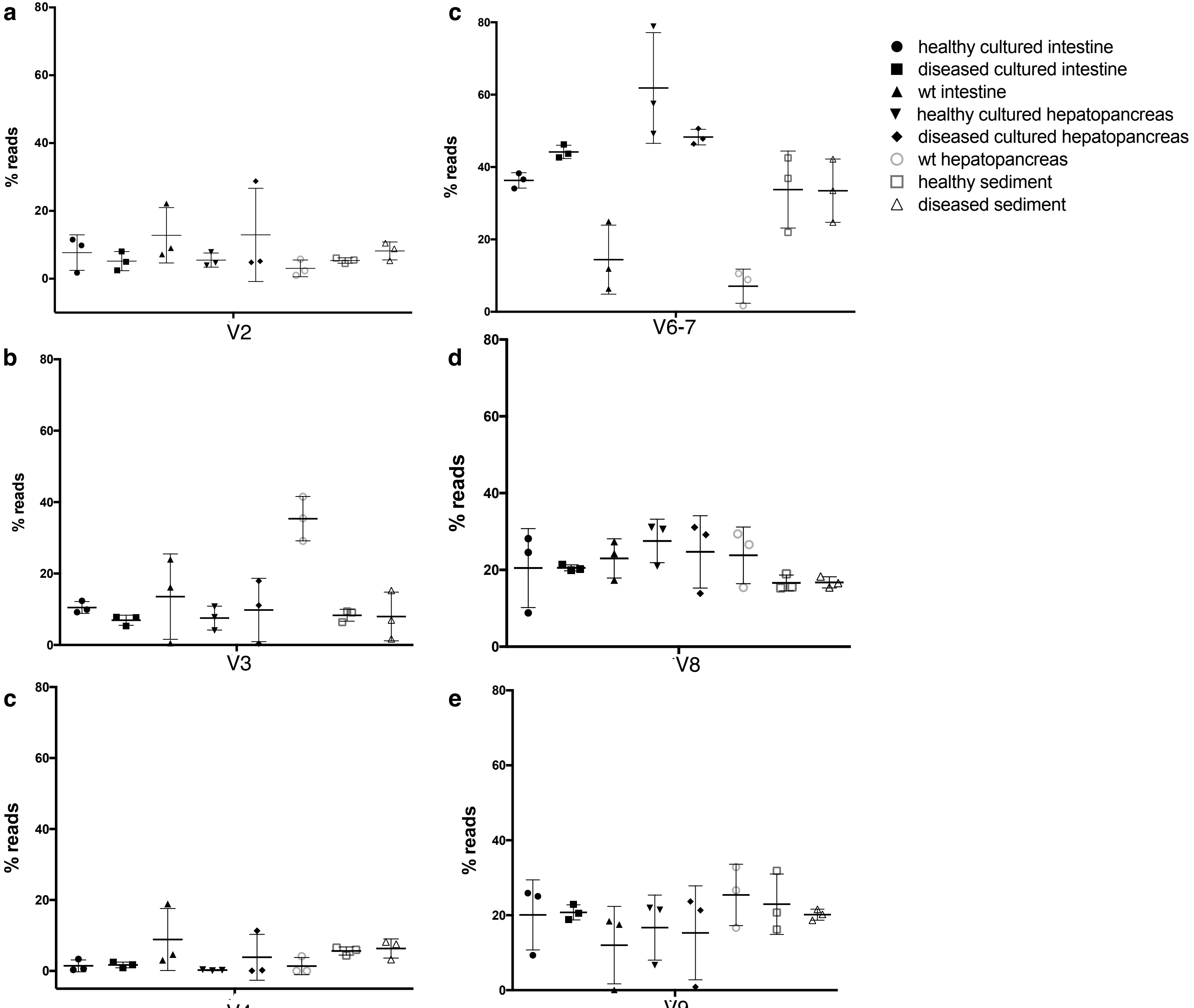


Supplementary Fig. S1

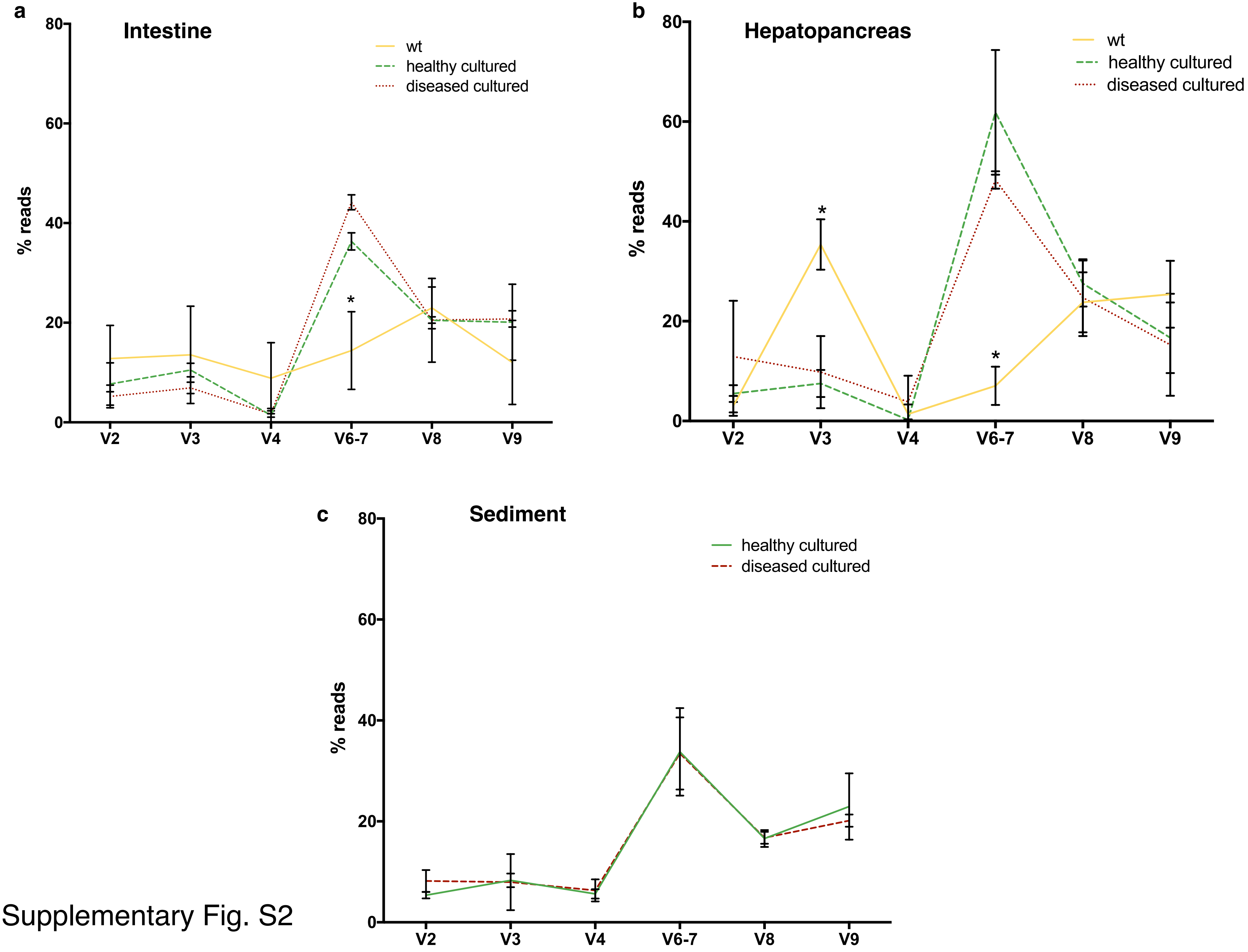


Supplementary Fig. S2

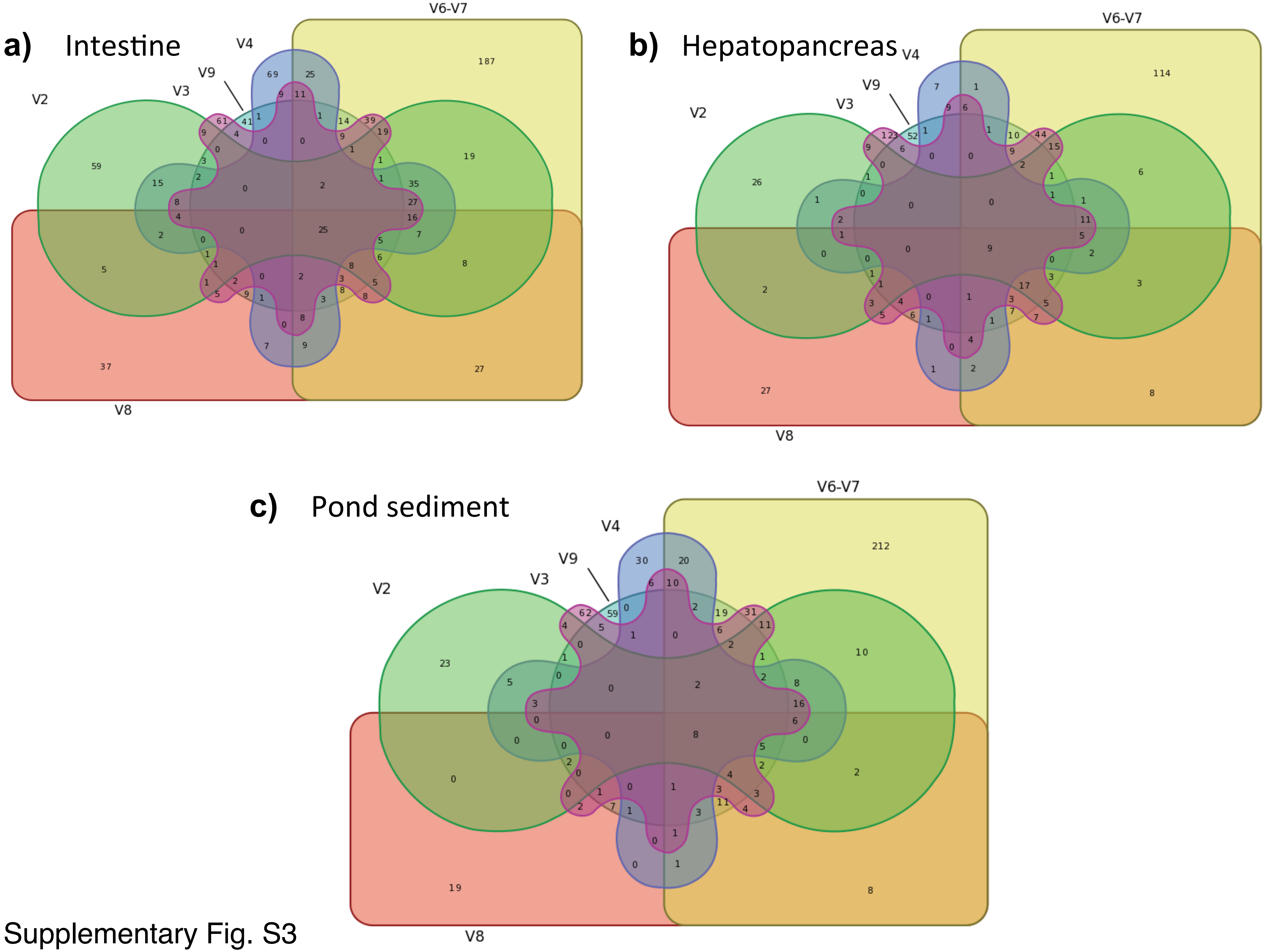


Supplementary Fig. S3

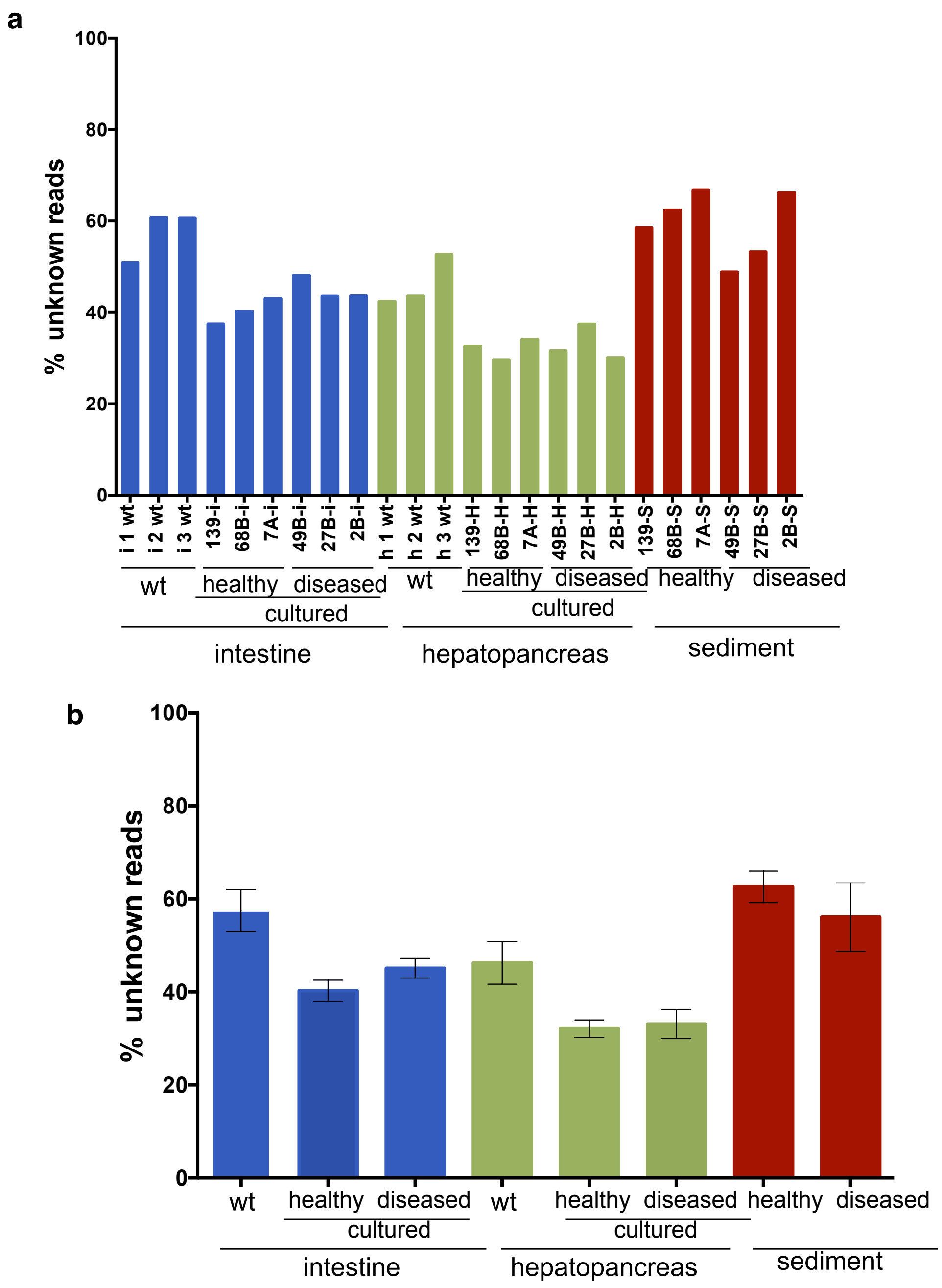


Supplementary Fig. S4

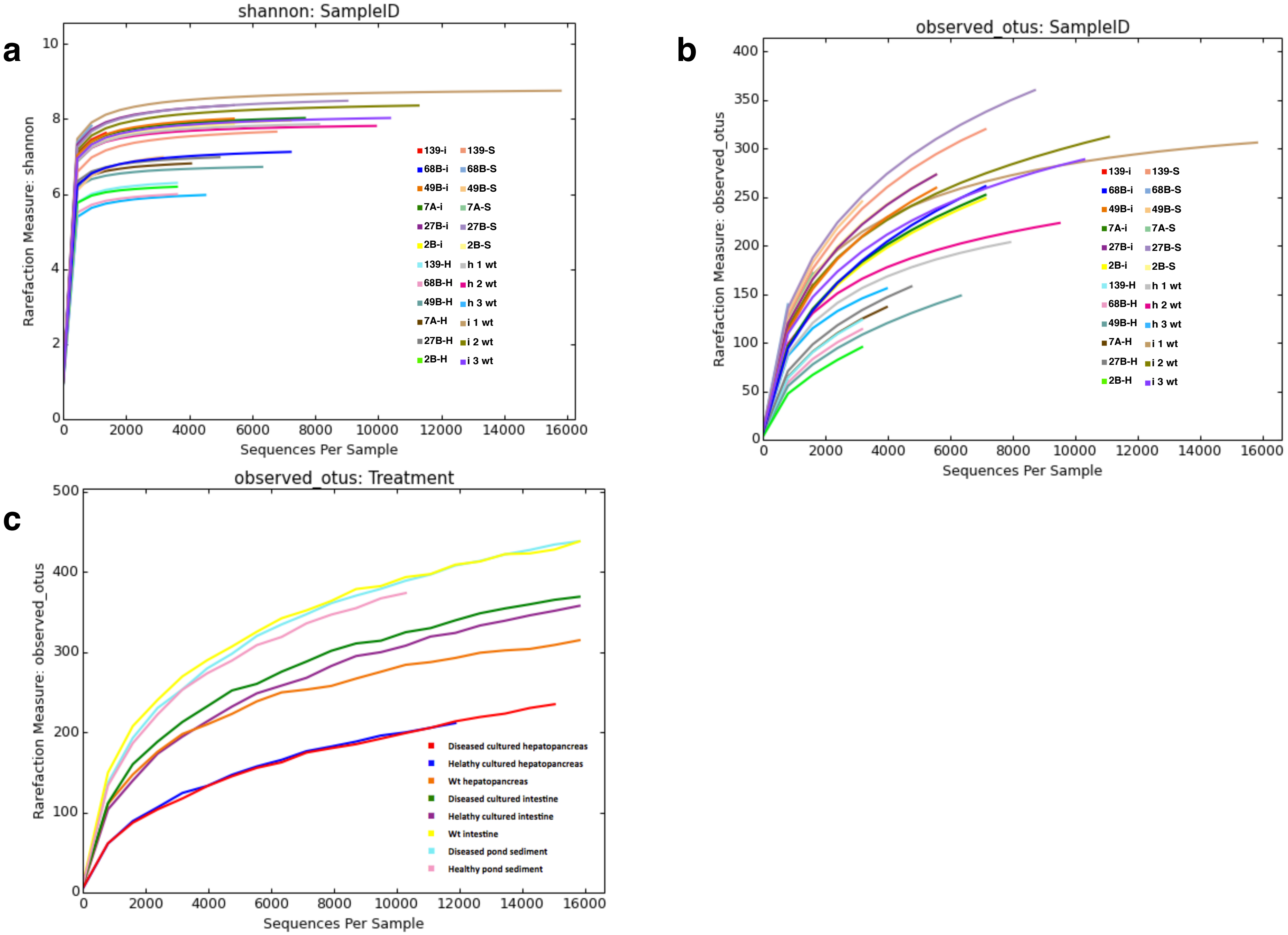


Supplementary Fig. S5

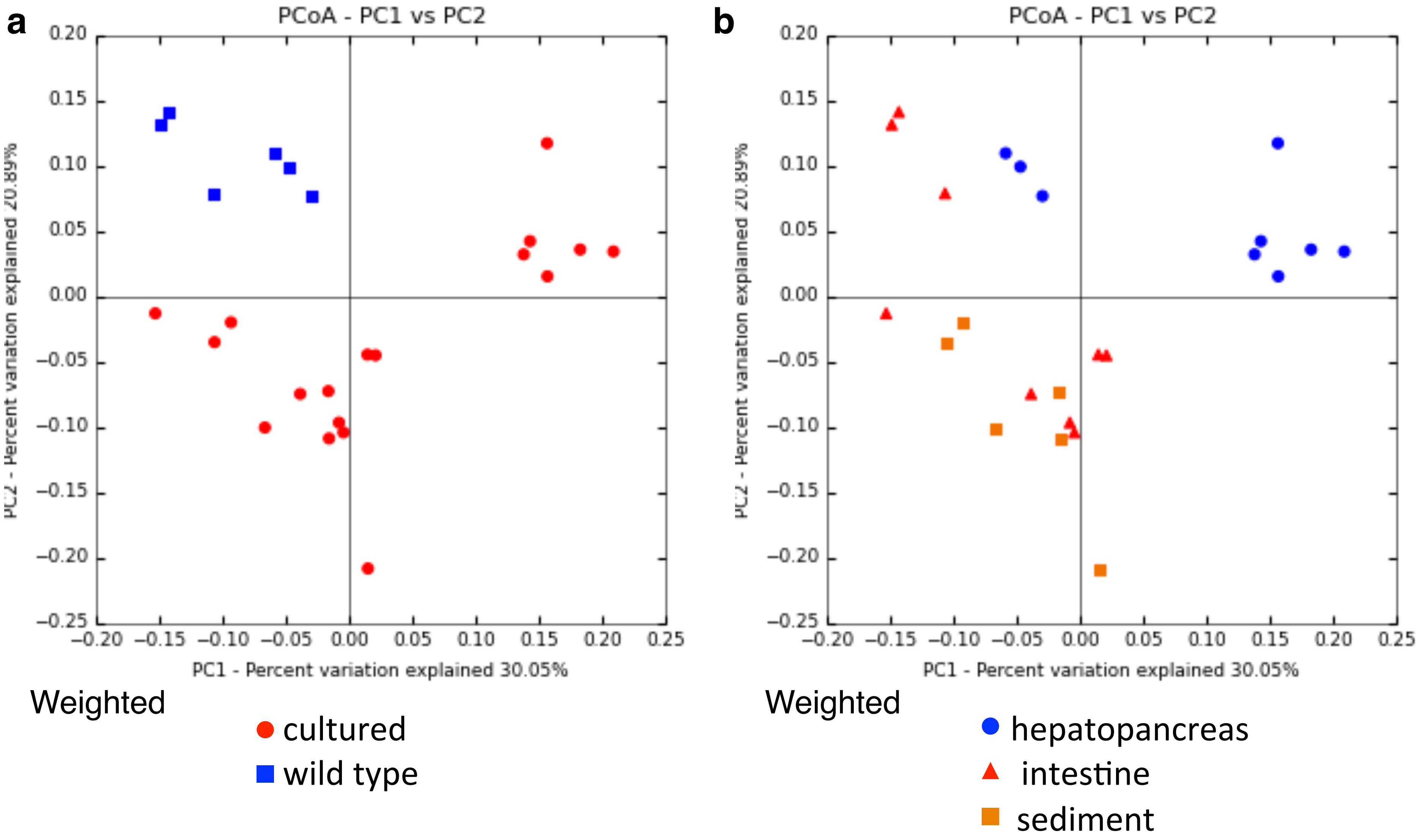


Supplementary Fig. S6

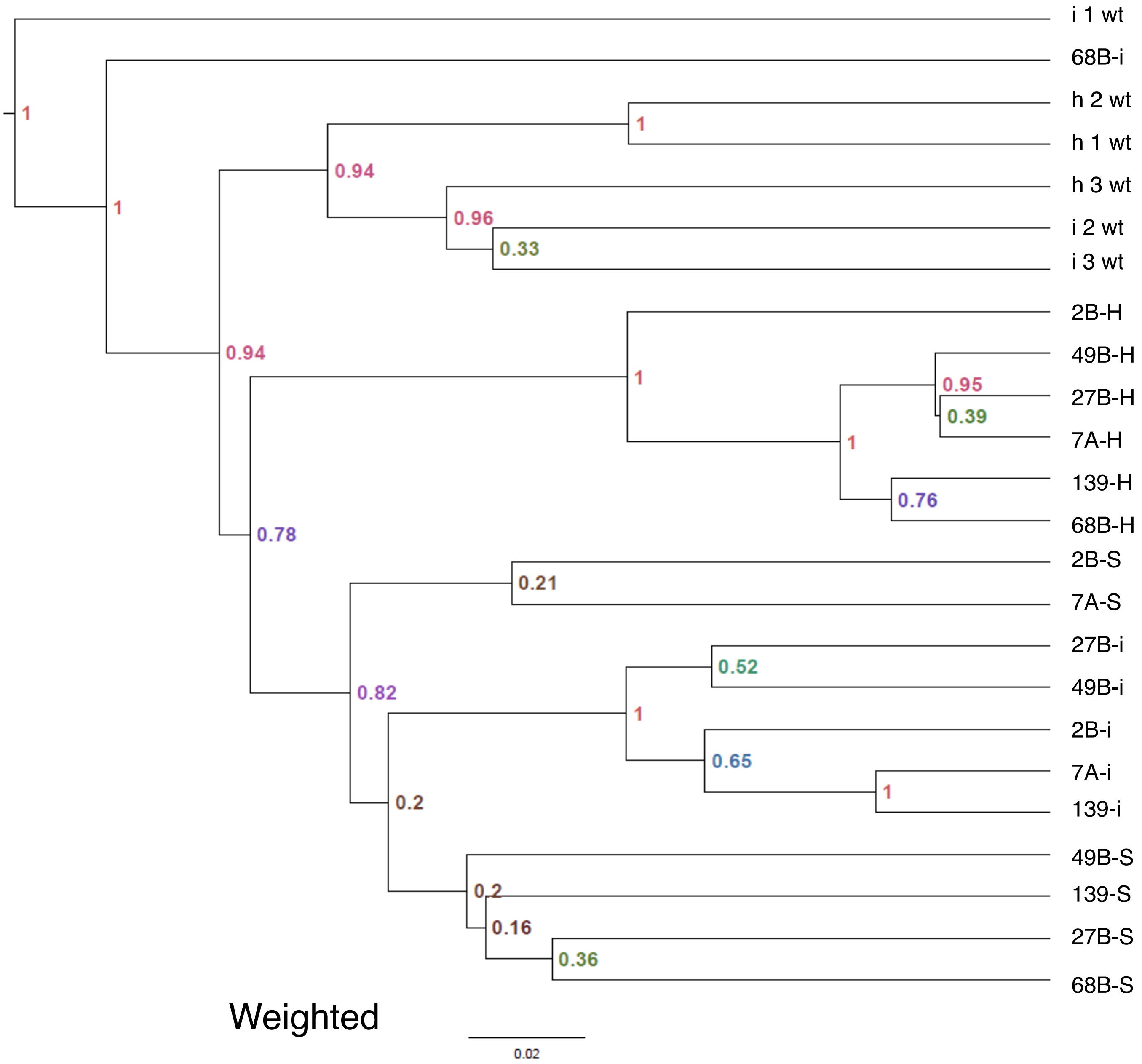


Supplementary Fig. S7

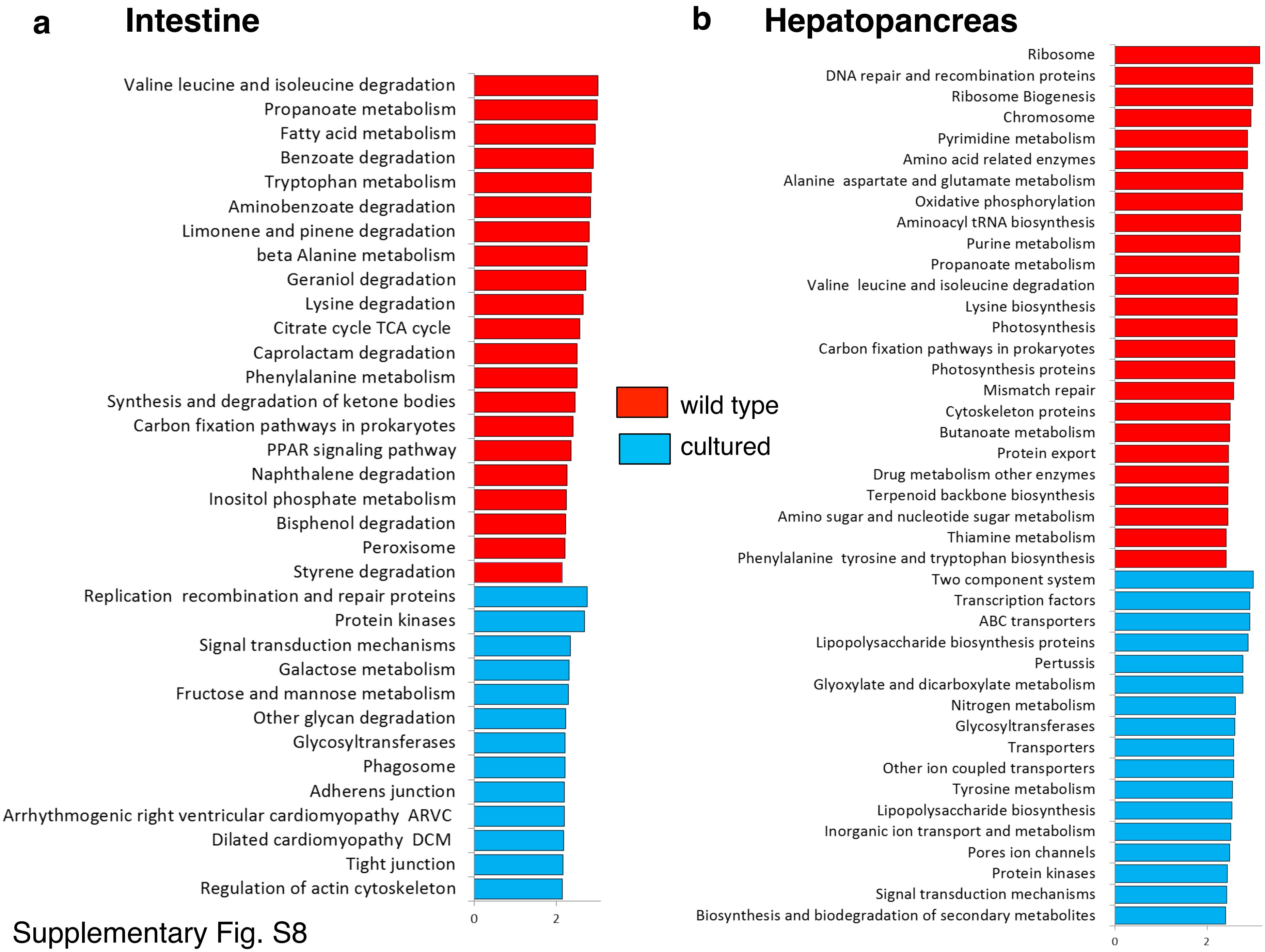


Supplementary Fig. S8

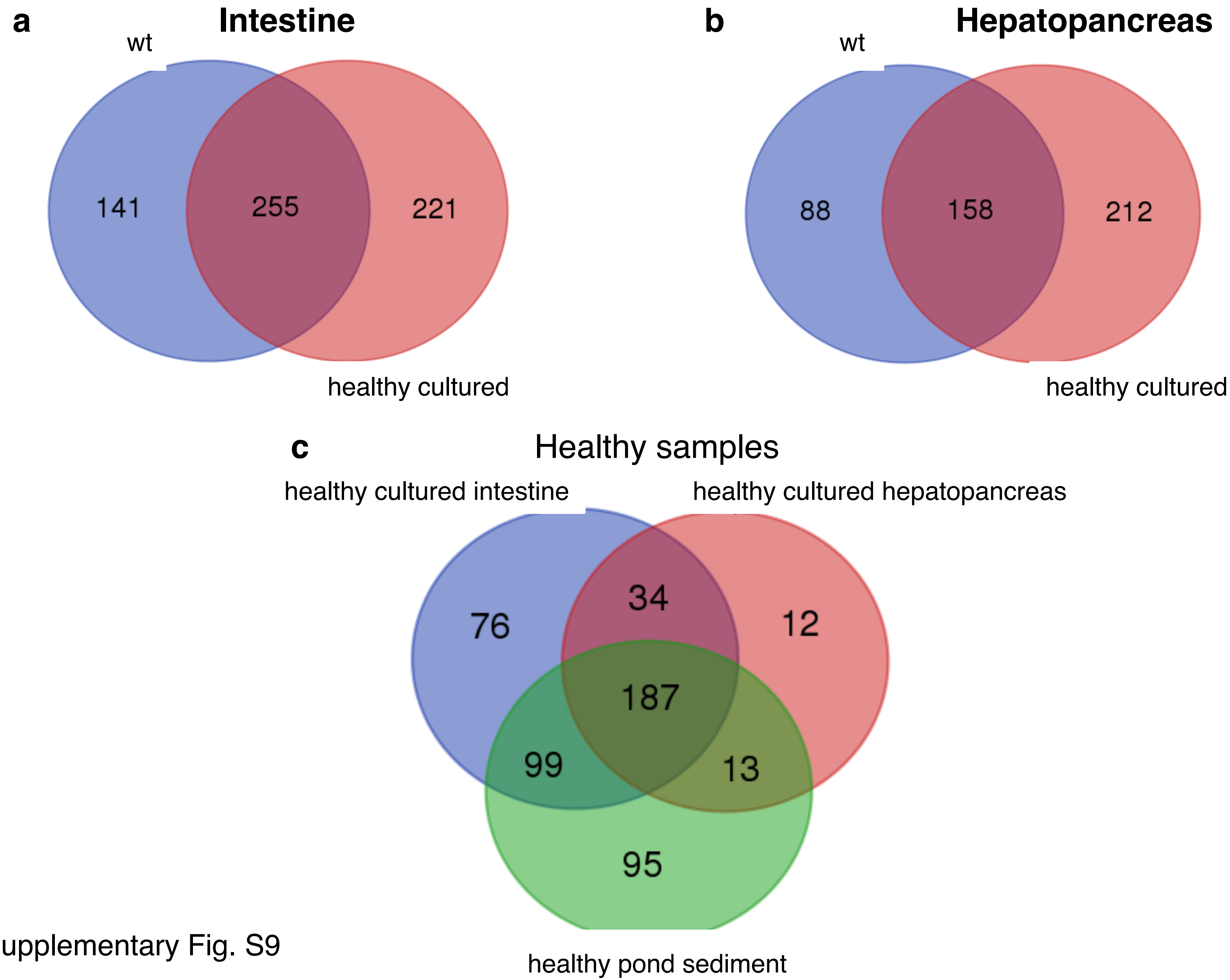


Supplementary Fig. S9

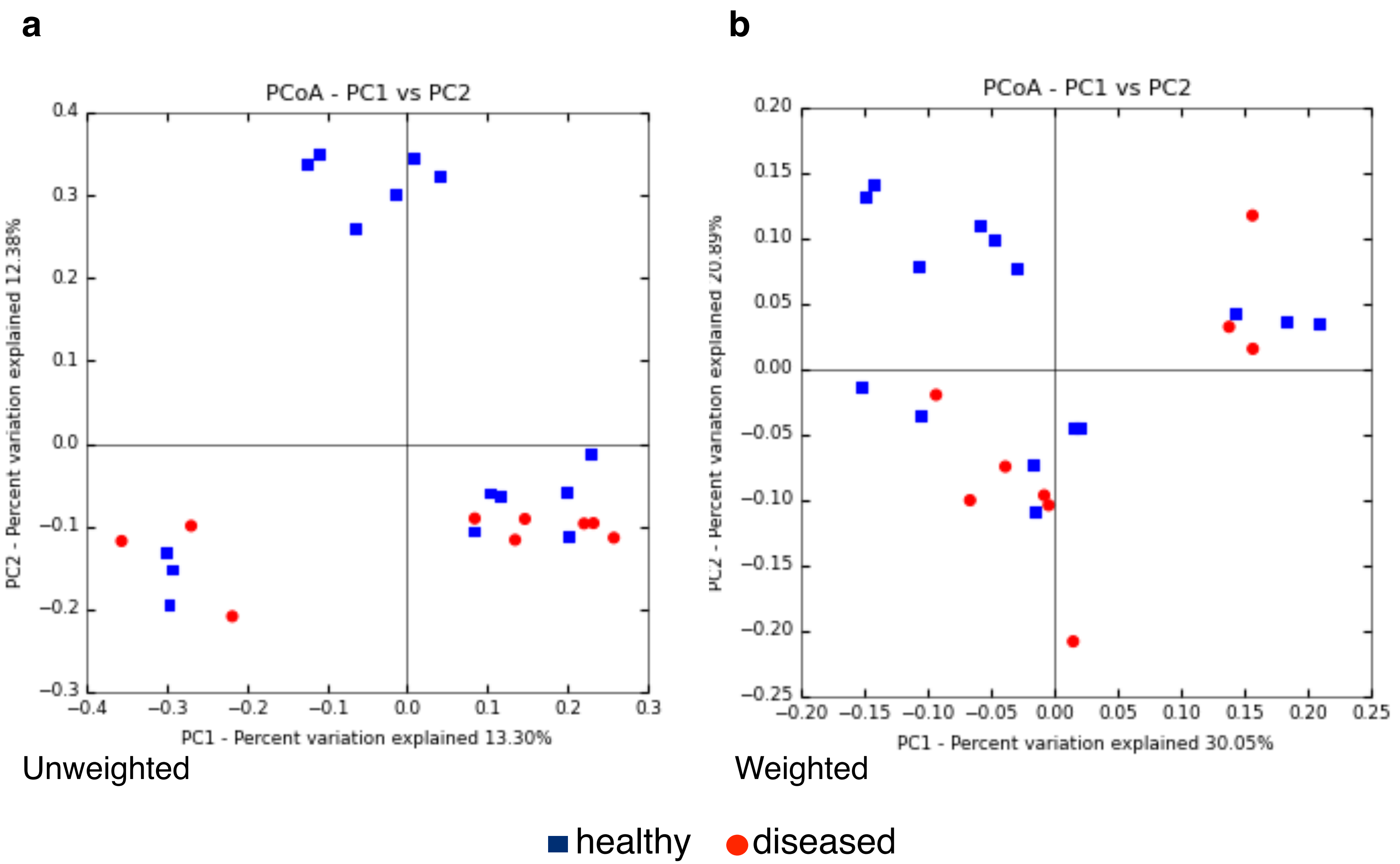


Supplementary Fig. S10

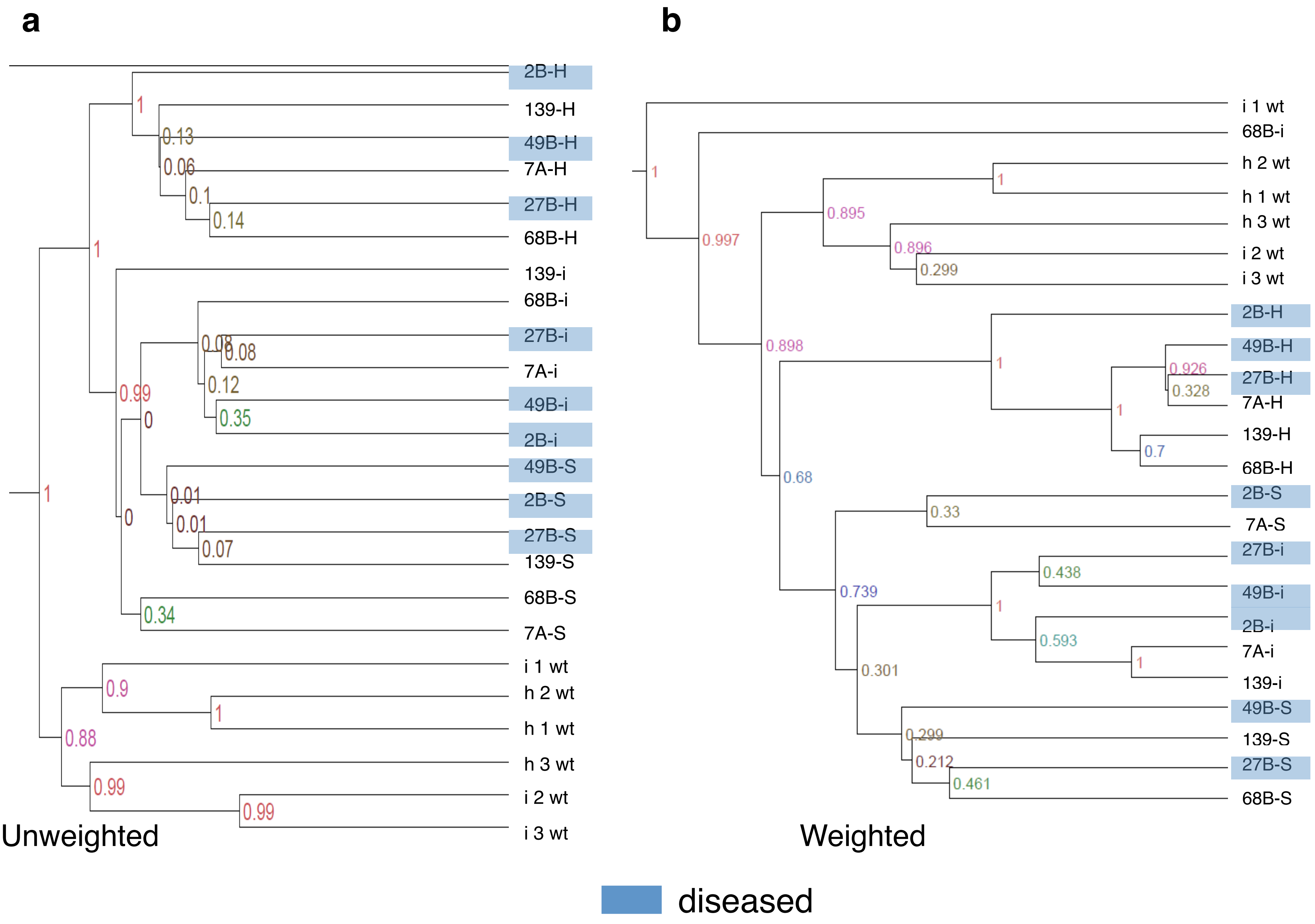


Supplementary Fig. S11

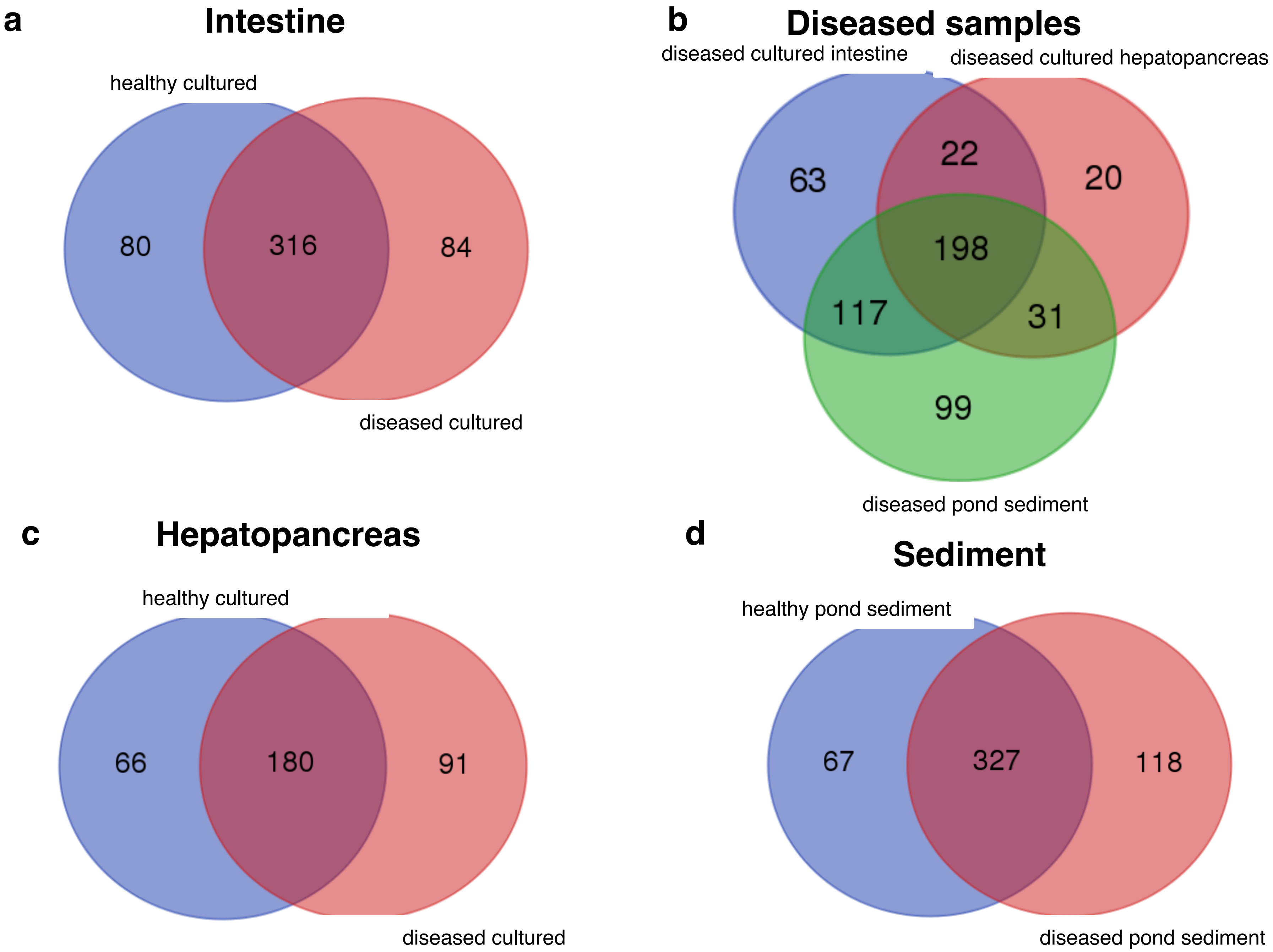


Supplementary Fig. S12

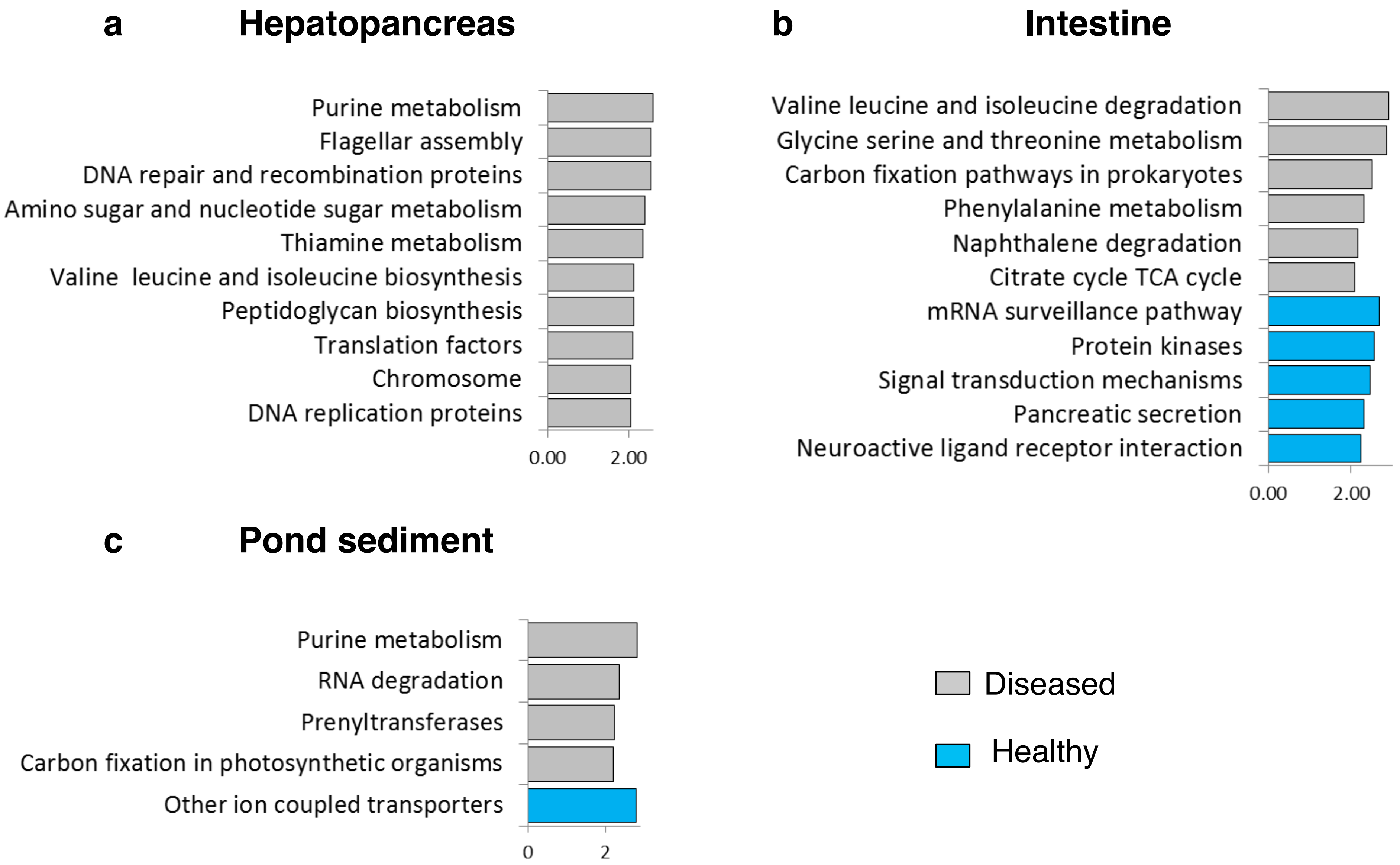


Supplementary Fig. S13

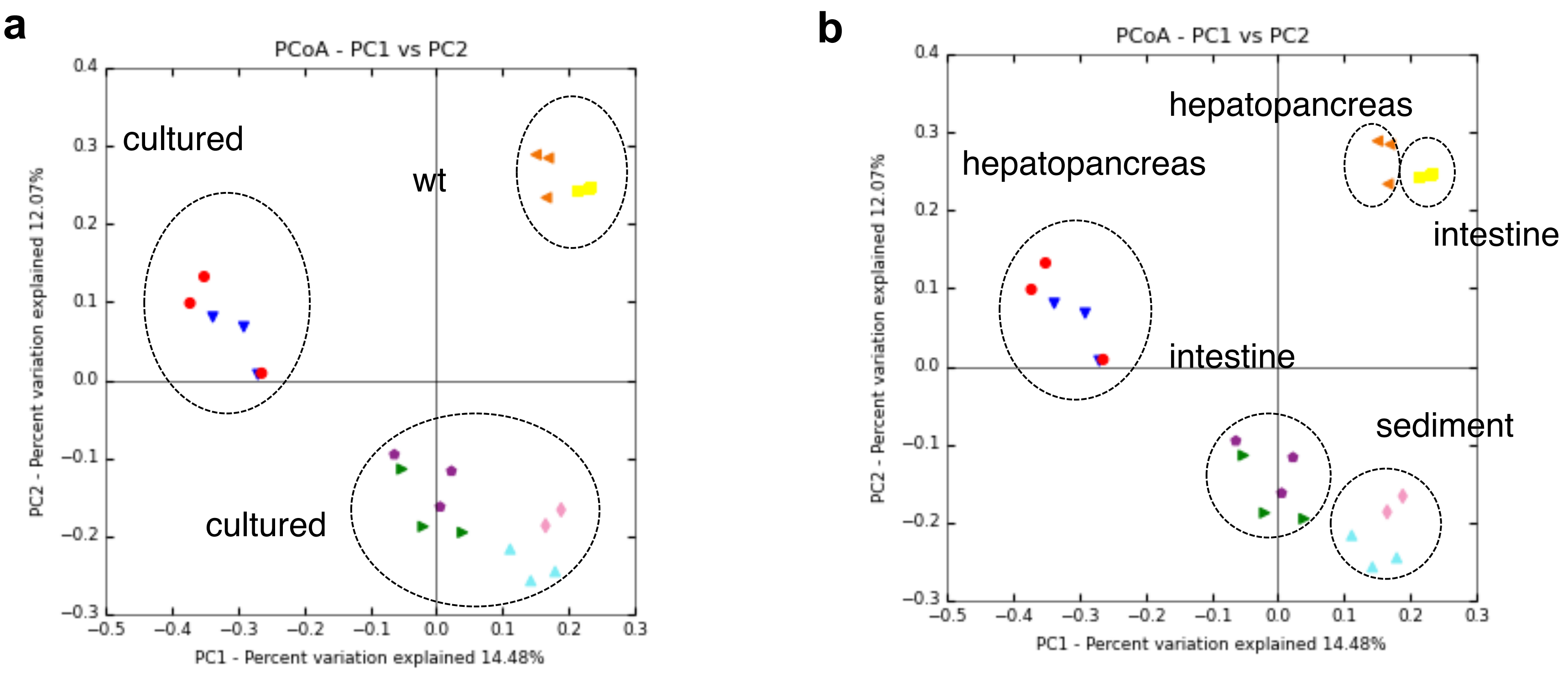


Unweighted

Supplementary Fig. S14

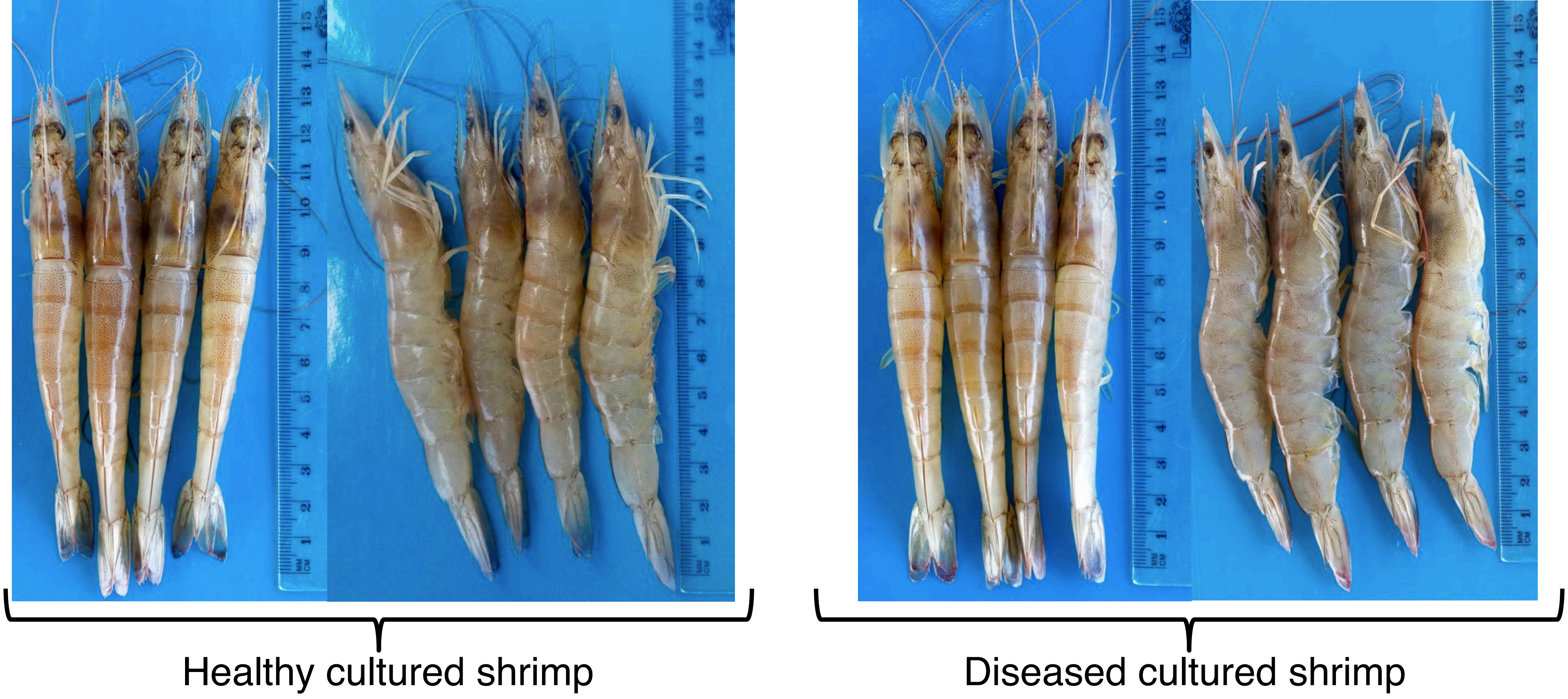


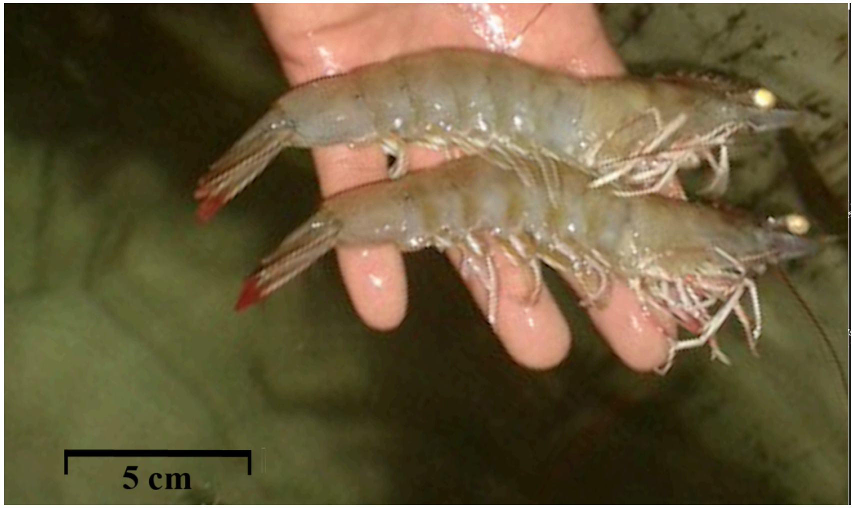


Supplementary Fig. S15

**Supplementary Table S1.** Relative abundance of OTUs with an abundance >0.1% at phylum level.

| | intestine | | | | | | | | | hepatopancreas | | | | | | | | | pond sediment | | | | | |
|---|---|---|---|---|---|---|---|---|---|---|---|---|---|---|---|---|---|---|---|---|---|---|---|---|
| | wt | | | cultured healthy | | | cultured diseased | | | wt | | | cultured healthy | | | cultured diseased | | | healthy | | | diseased | | |
| Phylum | i 1 wt | i 2 wt | i 3 wt | 139-i | 68B-i | 7A-i | 49B-i | 27B-i | 2B-i | h 1 wt | h 2 wt | h 3 wt | 139-H | 68B-H | 7A-H | 49B-H | 27B-H | 2B-H | 139-S | 68B-S | 7A-S | 49B-S | 27B-S | 2B-S |
| Acidobacteria | 0.00 | 0.00 | 0.00 | 0.28 | 0.26 | 0.26 | 0.12 | 0.34 | 0.24 | 0.00 | 0.00 | 0.00 | 0.07 | 0.07 | 0.17 | 0.14 | 0.18 | 0.27 | 0.32 | 0.71 | 3.86 | 0.71 | 3.81 | 3.80 |
| Actinobacteria | 1.35 | 0.85 | 3.04 | 1.59 | 1.15 | 0.98 | 0.79 | 1.06 | 0.61 | 7.45 | 0.11 | 2.40 | 0.57 | 0.20 | 0.23 | 0.37 | 0.53 | 0.31 | 16.73 | 13.35 | 3.68 | 50.53 | 6.44 | 3.95 |
| Bacteroidetes | 0.00 | 0.02 | 0.02 | 0.56 | 1.49 | 0.79 | 4.90 | 3.63 | 13.01 | 0.00 | 0.00 | 0.00 | 0.03 | 0.13 | 0.03 | 0.06 | 0.08 | 0.00 | 0.62 | 8.01 | 0.99 | 1.12 | 16.68 | 8.66 |
| Chloroflexi | 0.00 | 0.00 | 0.00 | 0.28 | 0.28 | 0.31 | 0.09 | 0.19 | 0.07 | 0.00 | 0.00 | 0.00 | 0.03 | 0.10 | 0.09 | 0.04 | 0.08 | 0.10 | 14.51 | 0.71 | 0.18 | 0.15 | 0.27 | 0.04 |
| Cyanobacteria | 0.00 | 9.09 | 29.67 | 4.68 | 62.47 | 5.85 | 9.14 | 17.35 | 9.68 | 0.11 | 0.17 | 0.89 | 0.91 | 0.75 | 0.52 | 0.49 | 0.96 | 0.71 | 1.24 | 3.91 | 3.77 | 2.99 | 3.61 | 1.10 |
| Firmicutes | 0.02 | 0.18 | 10.86 | 0.00 | 0.02 | 0.00 | 0.00 | 0.00 | 0.00 | 0.18 | 3.66 | 2.87 | 0.00 | 0.00 | 0.00 | 0.00 | 0.00 | 0.00 | 0.00 | 0.00 | 0.00 | 0.00 | 0.00 | 0.00 |
| Gemmatimonadetes | 0.00 | 0.00 | 0.00 | 1.78 | 2.45 | 1.67 | 0.82 | 3.91 | 1.81 | 0.00 | 0.00 | 0.00 | 0.64 | 0.85 | 1.27 | 0.49 | 1.47 | 1.15 | 6.65 | 12.46 | 44.70 | 9.39 | 12.07 | 34.34 |
| Proteobacteria | 98.63 | 89.86 | 56.42 | 90.54 | 28.19 | 89.18 | 74.64 | 65.83 | 70.12 | 92.27 | 96.06 | 93.04 | 97.67 | 97.75 | 97.60 | 98.43 | 96.63 | 97.39 | 59.88 | 60.50 | 42.55 | 34.60 | 56.60 | 47.35 |
| Spirochaetes | 0.00 | 0.00 | 0.00 | 0.28 | 0.09 | 0.16 | 5.60 | 6.20 | 1.44 | 0.00 | 0.00 | 0.00 | 0.07 | 0.10 | 0.06 | 0.00 | 0.08 | 0.00 | 0.05 | 0.36 | 0.27 | 0.36 | 0.46 | 0.77 |
| Tenericutes | 0.00 | 0.00 | 0.00 | 0.00 | 3.61 | 0.81 | 3.90 | 1.47 | 3.03 | 0.00 | 0.00 | 0.80 | 0.00 | 0.07 | 0.03 | 0.00 | 0.00 | 0.07 | 0.00 | 0.00 | 0.00 | 0.15 | 0.07 | 0.00 |

Data derived from OUT table with OTUs >0.1% of the total read count to simplify the visualization of the results.

**Supplementary Table S2.** Average relative abundance of top 5 OTUs with an abundance >0.1% at family and genus level.

**Intestine wt**

| Family | % | Genus | % |
|---|---|---|---|
| Vibrionaceae | 36.3 | Vibrio | 20.0 |
| Enterobacteriaceae | 15.3 | Photobacterium | 9.0 |
| Moraxellaceae | 7.3 | Paracoccus | 5.1 |
| Rhodobacteraceae | 6.5 | Fusibacter | 3.2 |
| Oxalobacteraceae | 5.5 | Acinetobacter | 3.2 |

**Hepatopancreas wt**

| Family | % | Genus | % |
|---|---|---|---|
| Enterobacteriaceae | 44.0 | Photobacterium | 16.0 |
| Vibrionaceae | 27.2 | Acinetobacter | 12.0 |
| Moraxellaceae | 12.0 | Vibrio | 7.8 |
| Shewanellaceae | 4.0 | Shewanella | 4.0 |
| Idiomarinaceae | 2.4 | Shigella | 3.9 |

**Helathy pond sediment**

| Family | % | Genus | % |
|---|---|---|---|
| Enterobacteriaceae | 19.8 | Acinetobacter | 12.7 |
| Moraxellaceae | 12.9 | Vibrio | 7.1 |
| Vibrionaceae | 10.9 | KSA1 | 2.7 |
| Microbacteriaceae | 8.0 | Enterobacter | 2.7 |
| koll13 | 3.0 | Pseudomonas | 2.6 |

**Healthy cultured intestine**

| Family | % | Genus | % |
|---|---|---|---|
| Vibrionaceae | 35.6 | Vibrio | 15.1 |
| Enterobacteriaceae | 20.1 | Pseudomonas | 3.3 |
| Pseudoalteromonadaceae | 5.9 | Photobacterium | 2.3 |
| Pseudomonadaceae | 3.3 | Enterobacter | 1.6 |
| Rhodobacteraceae | 1.3 | Escherichia | 1.3 |

**Healthy cultured hepatopancreas**

| Family | % | Genus | % |
|---|---|---|---|
| Enterobacteriaceae | 70.2 | Pseudomonas | 18.1 |
| Pseudomonadaceae | 18.1 | Vibrio | 3.0 |
| Vibrionaceae | 6.9 | Escherichia | 2.6 |
| Pseudoalteromonadaceae | 0.6 | Enterobacter | 2.6 |
| Moraxellaceae | 0.5 | Shigella | 2.4 |

**Diseased pond sediment**

| Family | % | Genus | % |
|---|---|---|---|
| Microbacteriaceae | 19.8 | KSA1 | 5.9 |
| Enterobacteriaceae | 12.0 | Vibrio | 5.4 |
| Vibrionaceae | 8.7 | Pseudidiomarina | 2.0 |
| [Balneolaceae] | 5.9 | Enterobacter | 2.0 |
| Desulfovibrionaceae | 4.3 | Acinetobacter | 2.0 |

**Diseased cultured intestine**

| Family | % | Genus | % |
|---|---|---|---|
| Vibrionaceae | 33.6 | Vibrio | 15.9 |
| Enterobacteriaceae | 18.6 | Photobacterium | 3.0 |
| Pseudoalteromonadaceae | 6.1 | Pseudomonas | 2.6 |
| Marinilabiaceae | 4.9 | Phaeobacter | 1.8 |
| Rhodobacteraceae | 4.2 | Enterobacter | 1.5 |

**Diseased cultured hepatopancreas**

| Family | % | Genus | % |
|---|---|---|---|
| Enterobacteriaceae | 77.6 | Pseudomonas | 10.4 |
| Pseudomonadaceae | 10.4 | Escherichia | 3.0 |
| Vibrionaceae | 7.3 | Shigella | 2.9 |
| Pseudoalteromonadaceae | 0.6 | Enterobacter | 2.9 |
| Comamonadaceae | 0.4 | Vibrio | 2.5 |

Data derived from OUT table with OTUs >0.1% of the total read count to simplify the visualization of the results.

**Supplementary Table S3**. Spearman correlation test between group triplicates.

| | | r Spearman | p-value |
|---|---|---|---|
| Intestine wt | i 1 wt vs i 2 wt | 0.555 | <0.0001 |
| | i 1 wt vs i 3 wt | 0.384 | <0.0001 |
| | i 2 wt vs i 3 wt | 0.871 | <0.0001 |
| Cultured healthy intestine | 139-i vs 68B-i | 0.766 | <0.0001 |
| | 139-i vs 7A-i | 0.864 | <0.0001 |
| | 68B-i vs 7A-i | 0.826 | <0.0001 |
| Cultured diseased intestine | 49B-i vs 27B-i | 0.869 | <0.0001 |
| | 49B-i vs 2B-i | 0.939 | <0.0001 |
| | 27B-i vs 2B-i | 0.866 | <0.0001 |
| Hepatopancreas wt | h 1 wt vs h 2 wt | 0.829 | <0.0001 |
| | h 1 wt vs h 3 wt | 0.587 | <0.0001 |
| | h 2 wt vs h 3 wt | 0.696 | <0.0001 |
| Cultured healthy hepatopancreas | 139-H vs 68B-H | 0.838 | <0.0001 |
| | 139-H vs 7A-H | 0.881 | <0.0001 |
| | 68B-H vs 7A-H | 0.875 | <0.0001 |
| Cultured diseased hepatopancreas | 49B-H vs 27B-H | 0.909 | <0.0001 |
| | 49B-H vs 2B-H | 0.834 | <0.0001 |
| | 27B-H vs 2B-H | 0.797 | <0.0001 |
| Helathy pond sediment | 139-S vs 68B-S | 0.837 | <0.0001 |
| | 139-S vs 7A-S | 0.807 | <0.0001 |
| | 68B-S vs 7A-S | 0.815 | <0.0001 |
| Diseased pond sediment | 49B-S vs 27B-S | 0.873 | <0.0001 |
| | 49B-S vs 2B-S | 0.882 | <0.0001 |
| | 27B-S vs 2B-S | 0.882 | <0.0001 |

**Supplementary Table S4**. Number of reads, observed OTUs and alpha diversity indices for all the sequenced samples.

| | wt intestine | | | healthy cultured intestine | | | diseased cultured intestine | | | wt hepatopancreas | | | healthy cultured hepatopancreas | | | diseased cultured hepatopancreas | | | helathy pond sediment | | | diseased pond sediment | | |
|---|---|---|---|---|---|---|---|---|---|---|---|---|---|---|---|---|---|---|---|---|---|---|---|---|
| | i 1 wt | i 2 wt | i 3 wt | 139-i | 68B-i | 7A-i | 49B-i | 27B-i | 2B-i | h 1 wt | h 2 wt | h 3 wt | 139-H | 68B-H | 7A-H | 49B-H | 27B-H | 2B-H | 139-S | 68B-S | 7A-S | 49B-S | 27B-S | 2B-S |
| Number of reads | 15814 | 11326 | 10723 | 1724 | 7543 | 7725 | 5830 | 5721 | 7554 | 8356 | 10030 | 4703 | 3700 | 3868 | 4452 | 6489 | 5147 | 3957 | 7211 | 1032 | 2078 | 3292 | 9434 | 5652 |
| Number of observed OTUs | 28.96 | 26.84 | 25.25 | - | 21.26 | 22.38 | 23.74 | 25.15 | 22.47 | 25.25 | 23.40 | 16.20 | 13.27 | 13.54 | 13.53 | 12.75 | 14.97 | 12.57 | 26.97 | - | 27.12 | 28.16 | 29.14 | 24.98 |
| Chao1 | 8.30 | 7.93 | 7.66 | - | 6.84 | 7.72 | 7.75 | 8.08 | 7.68 | 7.56 | 7.53 | 5.84 | 6.20 | 5.91 | 6.70 | 6.57 | 6.82 | 6.12 | 7.33 | - | 7.58 | 6.85 | 8.06 | 7.61 |
| Phylogenetic diversity (PD) | 49.62 | 45.76 | 42.84 | - | 35.68 | 37.04 | 39.73 | 42.22 | 37.27 | 42.93 | 39.27 | 26.55 | 20.34 | 21.17 | 20.36 | 18.92 | 23.11 | 19.01 | 46.60 | - | 46.67 | 49.47 | 50.22 | 42.35 |
| Shannon index | 8.30 | 7.93 | 7.66 | - | 6.84 | 7.72 | 7.75 | 8.08 | 7.68 | 7.56 | 7.53 | 5.84 | 6.20 | 5.91 | 6.70 | 6.57 | 6.82 | 6.12 | 7.33 | - | 7.58 | 6.85 | 8.06 | 7.61 |

**Supplementary Table S4**. Mean values of number of reads, observed OTUs and alpha diversity indices for all the sequenced samples.

| | Intestine | | | Hepatopancreas | | | Pond sediment | |
|---|---|---|---|---|---|---|---|---|
| | wt | healthy cultured | diseased cultured | wt | healthy cultured | diseased cultured | healthy | diseased |
| Number of reads | 12621.00 | 5664.00 | 6368.33 | 7696.33 | 4006.67 | 5197.67 | 3440.33 | 6126.00 |
| Number of observed OTUs | 27.02 | 21.82 | 23.79 | 21.61 | 13.45 | 13.43 | 27.04 | 27.43 |
| Chao1 | 7.96 | 7.28 | 7.84 | 6.98 | 6.27 | 6.51 | 7.45 | 7.51 |
| Phylogenetic diversity (PD) | 46.07 | 36.36 | 39.74 | 36.25 | 20.62 | 20.35 | 46.63 | 47.35 |
| Shannon index | 7.96 | 7.28 | 7.84 | 6.98 | 6.27 | 6.51 | 7.45 | 7.51 |

**Supplementary Table S5.** Good's Coverage of sequenced samples (10,000 iterations at 2,078 sequence depth).

| | Intestine | | | | | | | | | Hepatopancreas | | | | | | | | | Sediment | | | | | |
|---|---|---|---|---|---|---|---|---|---|---|---|---|---|---|---|---|---|---|---|---|---|---|---|---|
| | wt | | | healthy cultured | | | diseased cultured | | | wt | | | healthy cultured | | | diseased cultured | | | healthy | | | diseased | | |
| Sequence identity | i 1 wt | i 2 wt | i 3 wt | 139-i | 68B-i | 7A-i | 49B-i | 27B-i | 2B-i | h 1 wt | h 2 wt | h 3 wt | 139-H | 68B-H | 7A-H | 49B-H | 27B-H | 2B-H | 139-S | 68B-S | 7A-S | 49B-S | 27B-S | 2B-S |
| 97% | 0.814 | 0.806 | 0.835 | - | 0.848 | 0.846 | 0.833 | 0.813 | 0.85 | 0.847 | 0.869 | 0.902 | 0.909 | 0.910 | 0.909 | 0.919 | 0.8946 | 0.931 | 0.813 | - | 0.816 | 0.808 | 0.795 | 0.832 |

**Supplementary Table S5**. Mean values for good's coverage of sequenced samples (10,000 iterations at 2,078 sequence depth).

| | Intestine | | | Hepatopancreas | | | Sediment | |
|---|---|---|---|---|---|---|---|---|
| Sequence identity | wt | healthy cultured | diseased cultured | wt | healthy cultured | diseased cultured | healthy | diseased |
| 97% | 0.818 | 0.85 | 0.832 | 0.873 | 0.909 | 0.915 | 0.815 | 0.812 |

**Supplementary Table S6**. Linear Discriminant Analysis of enriched taxonomies in wt and cultured intestine samples using LEfSe.

**Cultured samples**

| Phyla | LDA |
|---|---|
| p_Bacteroidetes | 4.224 |
| p_Spirochaetes | 2.768 |
| p_Gemmatimonadetes | 3.907 |
| p_Fusobacteria | 3.143 |

| Class | LDA |
|---|---|
| c_Bacteroidia | 4.041 |
| c_Gemm_5 | 3.813 |
| c_Flavobacteriia | 3.571 |
| c_Gemm_2 | 3.383 |
| c_Fusobacteriia | 3.099 |
| c_TK17 | 2.980 |
| c_Rhodothermi | 2.976 |
| c_Nitriliruptoria | 2.902 |
| c_Epsilonproteobacteria | 2.824 |
| c_iii1_8 | 2.774 |
| c_Leptospirae | 2.764 |

| Order | LDA |
|---|---|
| o_Bacteroidales | 4.075 |
| o_Flavobacteriales | 3.592 |
| o_Legionellales | 3.294 |
| o_Desulfobacterales | 3.292 |
| o_Fusobacteriales | 3.109 |
| o_Rhodothermales | 2.975 |
| o_Nitriliruptorales | 2.898 |
| o_Campylobacterales | 2.800 |
| o_DS_18 | 2.796 |
| o_Leptospirales | 2.771 |
| o_Bifidobacteriales | 2.579 |
| o_SBR1031 | 2.490 |

| Family | LDA |
|---|---|
| f_Pseudoalteromonadaceae | 4.367 |
| f_Marinilabiaceae | 3.852 |
| f_Flavobacteriaceae | 3.558 |
| f_Alteromonadaceae | 3.429 |
| f_Lachnospiraceae | 3.396 |
| f_Desulfobulbaceae | 3.302 |
| f_Ruminococcaceae | 3.236 |
| f_Bacteroidaceae | 3.144 |
| f_Legionellaceae | 2.993 |
| f_Nitriliruptoraceae | 2.904 |
| f_Coxiellaceae | 2.902 |
| f_211ds20 | 2.876 |
| f_Balneolaceae | 2.812 |
| f_Psychromonadaceae | 2.734 |
| f_Helicobacteraceae | 2.666 |
| f_Rikenellaceae | 2.658 |
| f_Bifidobacteriaceae | 2.540 |
| f_Oceanospirillaceae | 2.533 |
| f_Rhodothermaceae | 2.502 |
| f_A4b | 2.493 |
| f_Flammeovirgaceae | 2.331 |
| f_Nannocystaceae | 2.313 |

**Wild-type samples**

| Phyla | LDA |
|---|---|
| p_Actinobacteria | 4.427 |
| p_Nitrospirae | 2.696 |

| Class | LDA |
|---|---|
| c_Nitrospira | 2.667 |
| c_RB25 | 2.763 |
| c_Synechococcophycideae | 4.063 |
| c_Thermoleophilia | 2.692 |

| Order | LDA |
|---|---|
| o_Caulobacterales | 4.032 |
| o_Rhizobiales | 4.006 |
| o_Rhodospirillales | 3.481 |
| o_Streptophyta | 3.342 |
| o_Thiohalorhabdales | 2.842 |
| o_Haptophyceae | 2.787 |
| o_Nitrospirales | 2.679 |
| o_MWH_UniP1 | 2.649 |
| o_Solirubrobacterales | 2.500 |

| Family | LDA |
|---|---|
| f_Moraxellaceae | 4.381 |
| f_Oxalobacteraceae | 4.378 |
| f_Caulobacteraceae | 4.005 |
| f_Ferrimonadaceae | 3.640 |
| f_Sphingomonadaceae | 3.547 |
| f_Aurantimonadaceae | 3.540 |
| f_Propionibacteriaceae | 3.355 |
| f_Hyphomicrobiaceae | 3.184 |
| f_Intrasporangiaceae | 2.984 |
| f_Methylocystaceae | 2.955 |
| f_Rhizobiaceae | 2.903 |
| f_Acetobacteraceae | 2.865 |
| f_Methylobacteriaceae | 2.748 |
| f_EB1017 | 2.709 |
| f_Dietziaceae | 2.687 |
| f_Phyllobacteriaceae | 2.629 |
| f_Streptomycetaceae | 2.291 |

| Genus | LDA |
|---|---|
| g_Pseudoalteromonas | 3.773 |
| g_Enterobacter | 3.722 |
| g_Escherichia | 3.533 |
| g_Robiginitalea | 3.267 |
| g_Phaeobacter | 3.265 |
| g_Trabulsiella | 3.249 |
| g_Shigella | 3.143 |
| g_Bacteroides | 3.123 |
| g_Agarivorans | 3.033 |
| g_Citrobacter | 2.865 |
| g_Bilophila | 2.834 |
| g_KSA1 | 2.767 |
| g_Brenneria | 2.747 |
| g_Aeromonas | 2.711 |
| g_Rhodovulum | 2.682 |
| g_Coprococcus | 2.678 |
| g_Microbulbifer | 2.632 |
| g_Faecalibacterium | 2.601 |
| g_Fusobacterium | 2.584 |
| g_Bifidobacterium | 2.561 |
| g_Blautia | 2.557 |
| g_Plesiocystis | 2.492 |
| g_Idiomarina | 2.483 |
| g_Clostridiisalibacter | 2.476 |
| g_Providencia | 2.391 |
| g_Butyrivibrio | 2.376 |
| g_Dickeya | 2.315 |
| g_Tolumonas | 2.280 |
| g_Loktanella | 2.262 |
| g_Marinobacter | 2.258 |

| Genus | LDA |
|---|---|
| g_Paracoccus | 4.165 |
| g_Rubellimicrobium | 3.664 |
| g_Ferrimonas | 3.626 |
| g_Janthinobacterium | 3.556 |
| g_Propionibacterium | 3.395 |
| g_Mycoplana | 3.339 |
| g_Rhodobacter | 3.290 |
| g_Agrococcus | 3.183 |
| g_Blastococcus | 3.152 |
| g_Ralstonia | 3.138 |
| g_Brevundimonas | 3.009 |
| g_Lysobacter | 2.993 |
| g_Pleomorphomonas | 2.923 |
| g_Nocardioides | 2.828 |
| g_Agrobacterium | 2.767 |
| g_Microbacterium | 2.724 |
| g_Variovorax | 2.664 |
| g_Dietzia | 2.659 |
| g_Methylobacterium | 2.548 |
| g_Microbispora | 2.543 |
| g_Jannaschia | 2.500 |
| g_Leptothrix | 2.454 |
| g_Serinicoccus | 2.392 |
| g_Streptomyces | 2.298 |
| g_Nitrobacteria | 2.200 |
| g_Rothia | 2.189 |
| g_Shinella | 2.176 |
| g_Novosphingobium | 2.162 |

**Supplementary Table S7.** Relative abundance of KEGG pathways obtained by Picrust analysis.

| Level 1 | Intestine wt | | | Cultured healthy intestine | | | Cultured diseased intestine | | | Hepatopancreas wt | | | Cultured healthy hepatopancreas | | | Cultured diseased hepatopancreas | | | Helathy pond sediment | | | Diseased pond sediment | | |
|---|---|---|---|---|---|---|---|---|---|---|---|---|---|---|---|---|---|---|---|---|---|---|---|---|
| KEGG_Pathways | i 1 wt | i 2 wt | i 3 wt | 139-i | 68B-i | 7A-i | 49B-i | 27B-i | 2B-i | h 1 wt | h 2 wt | h 3 wt | 139-H | 68B-H | 7A-H | 49B-H | 27B-H | 2B-H | 139-S | 68B-S | 7A-S | 49B-S | 27B-S | 2B-S |
| Cellular Processes | 0.045 | 0.054 | 0.048 | 0.052 | 0.039 | 0.052 | 0.049 | 0.043 | 0.049 | 0.036 | 0.037 | 0.048 | 0.042 | 0.041 | 0.041 | 0.040 | 0.042 | 0.039 | 0.040 | 0.039 | 0.039 | 0.036 | 0.046 | 0.040 |
| Environmental Information Processing | 0.164 | 0.159 | 0.152 | 0.170 | 0.145 | 0.168 | 0.155 | 0.154 | 0.157 | 0.163 | 0.165 | 0.164 | 0.179 | 0.179 | 0.179 | 0.180 | 0.178 | 0.180 | 0.142 | 0.153 | 0.142 | 0.153 | 0.132 | 0.129 |
| Genetic Information Processing | 0.147 | 0.154 | 0.159 | 0.153 | 0.161 | 0.154 | 0.156 | 0.155 | 0.156 | 0.161 | 0.163 | 0.157 | 0.150 | 0.149 | 0.151 | 0.152 | 0.152 | 0.152 | 0.167 | 0.162 | 0.175 | 0.170 | 0.172 | 0.173 |
| Human Diseases | 0.013 | 0.014 | 0.013 | 0.014 | 0.013 | 0.014 | 0.014 | 0.013 | 0.014 | 0.011 | 0.011 | 0.015 | 0.012 | 0.012 | 0.012 | 0.012 | 0.012 | 0.011 | 0.010 | 0.011 | 0.010 | 0.011 | 0.011 | 0.010 |
| Metabolism | 0.474 | 0.456 | 0.471 | 0.443 | 0.482 | 0.443 | 0.462 | 0.472 | 0.457 | 0.452 | 0.447 | 0.450 | 0.437 | 0.437 | 0.433 | 0.432 | 0.435 | 0.431 | 0.485 | 0.472 | 0.473 | 0.480 | 0.479 | 0.481 |
| None | 0.002 | 0.002 | 0.002 | 0.002 | 0.002 | 0.002 | 0.002 | 0.002 | 0.002 | 0.002 | 0.002 | 0.002 | 0.002 | 0.002 | 0.002 | 0.002 | 0.002 | 0.002 | 0.002 | 0.002 | 0.002 | 0.002 | 0.002 | 0.002 |
| Organismal Systems | 0.007 | 0.007 | 0.008 | 0.007 | 0.008 | 0.007 | 0.007 | 0.007 | 0.007 | 0.006 | 0.006 | 0.007 | 0.005 | 0.005 | 0.005 | 0.005 | 0.005 | 0.005 | 0.007 | 0.007 | 0.007 | 0.007 | 0.008 | 0.007 |
| Unclassified | 0.147 | 0.154 | 0.148 | 0.160 | 0.151 | 0.161 | 0.154 | 0.154 | 0.158 | 0.169 | 0.169 | 0.156 | 0.174 | 0.175 | 0.177 | 0.177 | 0.174 | 0.181 | 0.147 | 0.153 | 0.153 | 0.141 | 0.151 | 0.158 |

| Level 2 | | | | | | | | | | | | | | | | | | | | | | | | |
|---|---|---|---|---|---|---|---|---|---|---|---|---|---|---|---|---|---|---|---|---|---|---|---|---|
| KEGG_Pathways | i 1 wt | i 2 wt | i 3 wt | 139-i | 68B-i | 7A-i | 49B-i | 27B-i | 2B-i | h 1 wt | h 2 wt | h 3 wt | 139-H | 68B-H | 7A-H | 49B-H | 27B-H | 2B-H | 139-S | 68B-S | 7A-S | 49B-S | 27B-S | 2B-S |
| Amino Acid Metabolism | 0.101 | 0.095 | 0.095 | 0.090 | 0.091 | 0.090 | 0.096 | 0.099 | 0.092 | 0.090 | 0.088 | 0.092 | 0.087 | 0.087 | 0.085 | 0.084 | 0.086 | 0.082 | 0.104 | 0.098 | 0.100 | 0.103 | 0.102 | 0.103 |
| Biosynthesis of Other Secondary Metabolites | 0.008 | 0.006 | 0.007 | 0.006 | 0.009 | 0.006 | 0.007 | 0.007 | 0.007 | 0.006 | 0.006 | 0.006 | 0.006 | 0.006 | 0.006 | 0.006 | 0.006 | 0.006 | 0.008 | 0.008 | 0.008 | 0.009 | 0.008 | 0.009 |
| Cancers | 0.002 | 0.001 | 0.001 | 0.001 | 0.001 | 0.001 | 0.001 | 0.002 | 0.001 | 0.001 | 0.001 | 0.001 | 0.001 | 0.001 | 0.001 | 0.001 | 0.001 | 0.001 | 0.001 | 0.001 | 0.001 | 0.001 | 0.001 | 0.001 |
| Carbohydrate Metabolism | 0.095 | 0.090 | 0.092 | 0.092 | 0.090 | 0.092 | 0.094 | 0.094 | 0.094 | 0.096 | 0.096 | 0.091 | 0.093 | 0.093 | 0.095 | 0.095 | 0.094 | 0.096 | 0.094 | 0.096 | 0.091 | 0.100 | 0.092 | 0.092 |
| Cardiovascular Diseases | 0.000 | 0.000 | 0.000 | 0.000 | 0.000 | 0.000 | 0.000 | 0.000 | 0.000 | 0.000 | 0.000 | 0.000 | 0.000 | 0.000 | 0.000 | 0.000 | 0.000 | 0.000 | 0.000 | 0.000 | 0.000 | 0.000 | 0.000 | 0.000 |
| Cell Communication | 0.000 | 0.000 | 0.000 | 0.000 | 0.000 | 0.000 | 0.000 | 0.000 | 0.000 | 0.000 | 0.000 | 0.000 | 0.000 | 0.000 | 0.000 | 0.000 | 0.000 | 0.000 | 0.000 | 0.000 | 0.000 | 0.000 | 0.000 | 0.000 |
| Cell Growth and Death | 0.005 | 0.004 | 0.005 | 0.004 | 0.005 | 0.004 | 0.004 | 0.005 | 0.004 | 0.004 | 0.004 | 0.004 | 0.003 | 0.003 | 0.003 | 0.003 | 0.003 | 0.003 | 0.005 | 0.004 | 0.005 | 0.005 | 0.005 | 0.005 |
| Cell Motility | 0.037 | 0.046 | 0.040 | 0.045 | 0.031 | 0.046 | 0.042 | 0.036 | 0.042 | 0.030 | 0.031 | 0.041 | 0.037 | 0.036 | 0.036 | 0.035 | 0.037 | 0.034 | 0.033 | 0.032 | 0.032 | 0.028 | 0.038 | 0.032 |
| Cellular Processes and Signaling | 0.043 | 0.047 | 0.042 | 0.049 | 0.041 | 0.050 | 0.046 | 0.044 | 0.048 | 0.052 | 0.051 | 0.049 | 0.054 | 0.054 | 0.055 | 0.055 | 0.054 | 0.056 | 0.043 | 0.045 | 0.044 | 0.040 | 0.044 | 0.044 |
| Circulatory System | 0.001 | 0.001 | 0.000 | 0.000 | 0.000 | 0.000 | 0.001 | 0.000 | 0.000 | 0.000 | 0.000 | 0.001 | 0.000 | 0.000 | 0.000 | 0.000 | 0.000 | 0.000 | 0.000 | 0.000 | 0.000 | 0.000 | 0.001 | 0.000 |
| Digestive System | 0.000 | 0.001 | 0.001 | 0.001 | 0.001 | 0.001 | 0.001 | 0.000 | 0.001 | 0.000 | 0.000 | 0.000 | 0.000 | 0.000 | 0.000 | 0.000 | 0.000 | 0.000 | 0.000 | 0.000 | 0.000 | 0.000 | 0.000 | 0.001 |
| Endocrine System | 0.003 | 0.003 | 0.003 | 0.002 | 0.004 | 0.002 | 0.003 | 0.003 | 0.003 | 0.003 | 0.002 | 0.002 | 0.002 | 0.002 | 0.002 | 0.002 | 0.002 | 0.002 | 0.004 | 0.003 | 0.003 | 0.003 | 0.003 | 0.003 |
| Energy Metabolism | 0.053 | 0.054 | 0.064 | 0.053 | 0.076 | 0.053 | 0.055 | 0.058 | 0.057 | 0.051 | 0.050 | 0.053 | 0.049 | 0.049 | 0.048 | 0.049 | 0.049 | 0.048 | 0.054 | 0.054 | 0.055 | 0.053 | 0.058 | 0.055 |
| Environmental Adaptation | 0.001 | 0.002 | 0.002 | 0.002 | 0.001 | 0.002 | 0.002 | 0.001 | 0.002 | 0.001 | 0.001 | 0.002 | 0.001 | 0.001 | 0.001 | 0.001 | 0.001 | 0.001 | 0.002 | 0.001 | 0.001 | 0.001 | 0.002 | 0.001 |
| Enzyme Families | 0.019 | 0.020 | 0.021 | 0.020 | 0.023 | 0.020 | 0.019 | 0.020 | 0.020 | 0.020 | 0.020 | 0.020 | 0.020 | 0.020 | 0.020 | 0.020 | 0.020 | 0.020 | 0.019 | 0.020 | 0.022 | 0.019 | 0.020 | 0.021 |
| Excretory System | 0.000 | 0.000 | 0.000 | 0.000 | 0.000 | 0.000 | 0.000 | 0.000 | 0.000 | 0.000 | 0.000 | 0.000 | 0.000 | 0.000 | 0.000 | 0.000 | 0.000 | 0.000 | 0.000 | 0.000 | 0.000 | 0.000 | 0.000 | 0.000 |
| Folding, Sorting and Degradation | 0.022 | 0.024 | 0.025 | 0.023 | 0.026 | 0.024 | 0.024 | 0.023 | 0.024 | 0.024 | 0.024 | 0.024 | 0.023 | 0.023 | 0.023 | 0.023 | 0.023 | 0.023 | 0.025 | 0.024 | 0.025 | 0.024 | 0.027 | 0.025 |
| Genetic Information Processing | 0.025 | 0.025 | 0.027 | 0.027 | 0.029 | 0.027 | 0.027 | 0.028 | 0.027 | 0.030 | 0.031 | 0.027 | 0.030 | 0.030 | 0.031 | 0.031 | 0.030 | 0.032 | 0.026 | 0.027 | 0.028 | 0.025 | 0.028 | 0.028 |
| Glycan Biosynthesis and Metabolism | 0.018 | 0.021 | 0.020 | 0.023 | 0.021 | 0.023 | 0.022 | 0.021 | 0.023 | 0.024 | 0.024 | 0.023 | 0.025 | 0.025 | 0.026 | 0.026 | 0.025 | 0.026 | 0.021 | 0.022 | 0.023 | 0.020 | 0.022 | 0.024 |
| Immune System | 0.000 | 0.001 | 0.001 | 0.001 | 0.001 | 0.001 | 0.001 | 0.001 | 0.001 | 0.001 | 0.001 | 0.001 | 0.001 | 0.001 | 0.001 | 0.001 | 0.001 | 0.001 | 0.001 | 0.001 | 0.001 | 0.000 | 0.001 | 0.001 |
| Immune System Diseases | 0.000 | 0.001 | 0.001 | 0.001 | 0.000 | 0.001 | 0.001 | 0.000 | 0.001 | 0.001 | 0.001 | 0.001 | 0.001 | 0.001 | 0.000 | 0.000 | 0.000 | 0.000 | 0.001 | 0.001 | 0.000 | 0.001 | 0.000 | 0.000 |
| Infectious Diseases | 0.005 | 0.008 | 0.007 | 0.008 | 0.007 | 0.009 | 0.008 | 0.006 | 0.008 | 0.006 | 0.006 | 0.009 | 0.007 | 0.007 | 0.007 | 0.007 | 0.007 | 0.007 | 0.005 | 0.006 | 0.005 | 0.005 | 0.005 | 0.005 |
| Lipid Metabolism | 0.035 | 0.034 | 0.033 | 0.031 | 0.030 | 0.031 | 0.033 | 0.033 | 0.032 | 0.031 | 0.031 | 0.033 | 0.031 | 0.031 | 0.030 | 0.029 | 0.030 | 0.029 | 0.037 | 0.035 | 0.035 | 0.033 | 0.035 | 0.035 |
| Membrane Transport | 0.137 | 0.128 | 0.124 | 0.138 | 0.119 | 0.136 | 0.126 | 0.127 | 0.127 | 0.135 | 0.138 | 0.135 | 0.147 | 0.147 | 0.148 | 0.149 | 0.147 | 0.149 | 0.117 | 0.127 | 0.116 | 0.130 | 0.107 | 0.104 |
| Metabolic Diseases | 0.001 | 0.001 | 0.001 | 0.001 | 0.001 | 0.001 | 0.001 | 0.001 | 0.001 | 0.001 | 0.001 | 0.001 | 0.001 | 0.001 | 0.001 | 0.001 | 0.001 | 0.001 | 0.001 | 0.001 | 0.001 | 0.001 | 0.001 | 0.001 |
| Metabolism | 0.027 | 0.026 | 0.026 | 0.028 | 0.027 | 0.028 | 0.027 | 0.028 | 0.028 | 0.030 | 0.030 | 0.027 | 0.031 | 0.032 | 0.032 | 0.032 | 0.032 | 0.034 | 0.024 | 0.027 | 0.027 | 0.025 | 0.025 | 0.030 |
| Metabolism of Cofactors and Vitamins | 0.041 | 0.041 | 0.045 | 0.040 | 0.050 | 0.041 | 0.042 | 0.043 | 0.042 | 0.042 | 0.040 | 0.041 | 0.039 | 0.039 | 0.039 | 0.039 | 0.039 | 0.039 | 0.043 | 0.042 | 0.044 | 0.043 | 0.045 | 0.044 |
| Metabolism of Other Amino Acids | 0.020 | 0.018 | 0.017 | 0.017 | 0.017 | 0.017 | 0.018 | 0.018 | 0.017 | 0.017 | 0.017 | 0.018 | 0.017 | 0.017 | 0.017 | 0.017 | 0.017 | 0.017 | 0.018 | 0.018 | 0.017 | 0.018 | 0.018 | 0.018 |
| Metabolism of Terpenoids and Polyketides | 0.020 | 0.018 | 0.019 | 0.016 | 0.019 | 0.016 | 0.018 | 0.019 | 0.017 | 0.017 | 0.016 | 0.017 | 0.015 | 0.015 | 0.015 | 0.015 | 0.015 | 0.014 | 0.021 | 0.019 | 0.019 | 0.020 | 0.020 | 0.020 |
| Nervous System | 0.001 | 0.001 | 0.001 | 0.001 | 0.001 | 0.001 | 0.001 | 0.001 | 0.001 | 0.001 | 0.001 | 0.001 | 0.001 | 0.001 | 0.001 | 0.001 | 0.001 | 0.001 | 0.001 | 0.001 | 0.001 | 0.001 | 0.001 | 0.001 |
| Neurodegenerative Diseases | 0.004 | 0.003 | 0.003 | 0.003 | 0.002 | 0.003 | 0.003 | 0.003 | 0.003 | 0.002 | 0.002 | 0.003 | 0.002 | 0.002 | 0.002 | 0.002 | 0.002 | 0.002 | 0.003 | 0.002 | 0.002 | 0.003 | 0.003 | 0.003 |
| Nucleotide Metabolism | 0.031 | 0.032 | 0.033 | 0.032 | 0.033 | 0.032 | 0.033 | 0.032 | 0.033 | 0.033 | 0.033 | 0.034 | 0.030 | 0.030 | 0.031 | 0.031 | 0.031 | 0.031 | 0.034 | 0.033 | 0.035 | 0.035 | 0.035 | 0.035 |
| Poorly Characterized | 0.052 | 0.055 | 0.053 | 0.056 | 0.053 | 0.057 | 0.055 | 0.054 | 0.055 | 0.058 | 0.058 | 0.053 | 0.059 | 0.059 | 0.059 | 0.059 | 0.059 | 0.059 | 0.054 | 0.054 | 0.055 | 0.051 | 0.054 | 0.056 |
| Replication and Repair | 0.064 | 0.065 | 0.068 | 0.065 | 0.070 | 0.065 | 0.066 | 0.066 | 0.067 | 0.069 | 0.070 | 0.067 | 0.063 | 0.063 | 0.063 | 0.064 | 0.064 | 0.063 | 0.073 | 0.070 | 0.076 | 0.075 | 0.075 | 0.076 |
| Sensory System | 0.000 | 0.000 | 0.000 | 0.000 | 0.000 | 0.000 | 0.000 | 0.000 | 0.000 | 0.000 | 0.000 | 0.000 | 0.000 | 0.000 | 0.000 | 0.000 | 0.000 | 0.000 | 0.000 | 0.000 | 0.000 | 0.000 | 0.000 | 0.000 |
| Signal Transduction | 0.026 | 0.029 | 0.027 | 0.030 | 0.025 | 0.030 | 0.028 | 0.026 | 0.028 | 0.026 | 0.026 | 0.027 | 0.030 | 0.030 | 0.030 | 0.030 | 0.030 | 0.029 | 0.023 | 0.025 | 0.025 | 0.022 | 0.023 | 0.023 |
| Signaling Molecules and Interaction | 0.002 | 0.002 | 0.002 | 0.002 | 0.002 | 0.002 | 0.002 | 0.002 | 0.002 | 0.002 | 0.002 | 0.002 | 0.002 | 0.002 | 0.001 | 0.001 | 0.001 | 0.001 | 0.002 | 0.002 | 0.001 | 0.002 | 0.001 | 0.002 |
| Transcription | 0.025 | 0.025 | 0.024 | 0.026 | 0.022 | 0.026 | 0.025 | 0.024 | 0.025 | 0.027 | 0.027 | 0.025 | 0.028 | 0.028 | 0.029 | 0.029 | 0.028 | 0.029 | 0.024 | 0.026 | 0.025 | 0.025 | 0.023 | 0.025 |
| Translation | 0.037 | 0.040 | 0.043 | 0.039 | 0.043 | 0.039 | 0.041 | 0.041 | 0.041 | 0.042 | 0.042 | 0.041 | 0.036 | 0.036 | 0.037 | 0.037 | 0.037 | 0.036 | 0.046 | 0.043 | 0.049 | 0.046 | 0.048 | 0.048 |
| Transport and Catabolism | 0.003 | 0.003 | 0.003 | 0.003 | 0.003 | 0.003 | 0.003 | 0.003 | 0.003 | 0.002 | 0.002 | 0.003 | 0.002 | 0.002 | 0.002 | 0.002 | 0.002 | 0.002 | 0.003 | 0.003 | 0.003 | 0.003 | 0.003 | 0.003 |
| Xenobiotics Biodegradation and Metabolism | 0.035 | 0.028 | 0.026 | 0.024 | 0.024 | 0.023 | 0.027 | 0.029 | 0.024 | 0.026 | 0.026 | 0.024 | 0.024 | 0.025 | 0.023 | 0.022 | 0.023 | 0.022 | 0.032 | 0.028 | 0.025 | 0.026 | 0.026 | 0.026 |

| Level 3 | | | | | | | | | | | | | | | | | | | | | | | | |
|---|---|---|---|---|---|---|---|---|---|---|---|---|---|---|---|---|---|---|---|---|---|---|---|---|
| KEGG_Pathways | i 1 wt | i 2 wt | i 3 wt | 139-i | 68B-i | 7A-i | 49B-i | 27B-i | 2B-i | h 1 wt | h 2 wt | h 3 wt | 139-H | 68B-H | 7A-H | 49B-H | 27B-H | 2B-H | 139-S | 68B-S | 7A-S | 49B-S | 27B-S | 2B-S |
| 1,1,1-Trichloro-2,2-bis(4-chlorophenyl)ethane (DDT) degradation | 4.18E-06 | 1.39E-05 | 5.57E-06 | 5.27E-06 | 1.05E-05 | 5.79E-06 | 6.38E-06 | 7.17E-06 | 6.90E-06 | 9.09E-06 | 9.31E-06 | 2.98E-06 | 2.65E-06 | 2.10E-06 | 2.73E-06 | 5.07E-06 | 4.78E-06 | 1.17E-07 | 8.54E-06 | 4.70E-06 | 7.14E-06 | 1.28E-05 | 1.14E-05 | 6.99E-05 |
| ABC transporters | 4.01E-02 | 3.35E-02 | 3.42E-02 | 3.64E-02 | 3.40E-02 | 3.55E-02 | 3.39E-02 | 3.61E-02 | 3.38E-02 | 3.61E-02 | 3.62E-02 | 3.38E-02 | 3.89E-02 | 3.88E-02 | 3.91E-02 | 3.91E-02 | 3.89E-02 | 3.93E-02 | 3.32E-02 | 3.53E-02 | 3.22E-02 | 3.70E-02 | 2.83E-02 | 2.84E-02 |
| Adherens junction | 0.00E+00 | 0.00E+00 | 0.00E+00 | 1.46E-07 | 4.17E-08 | 3.29E-08 | 0.00E+00 | 9.96E-08 | 0.00E+00 | 0.00E+00 | 0.00E+00 | 0.00E+00 | 0.00E+00 | 0.00E+00 | 0.00E+00 | 3.60E-08 | 0.00E+00 | 0.00E+00 | 4.55E-08 | 0.00E+00 | 0.00E+00 | 5.15E-07 | 1.37E-06 | 1.83E-07 |
| Adipocytokine signaling pathway | 7.21E-04 | 6.57E-04 | 7.08E-04 | 5.53E-04 | 6.61E-04 | 5.55E-04 | 6.84E-04 | 6.68E-04 | 7.01E-04 | 4.99E-04 | 4.35E-04 | 5.08E-04 | 3.88E-04 | 3.86E-04 | 3.59E-04 | 3.48E-04 | 3.84E-04 | 3.22E-04 | 7.81E-04 | 7.10E-04 | 7.40E-04 | 8.55E-04 | 7.92E-04 | 6.98E-04 |
| African trypanosomiasis | 2.13E-04 | 1.71E-04 | 1.17E-04 | 2.11E-04 | 1.04E-04 | 2.07E-04 | 1.66E-04 | 1.51E-04 | 1.83E-04 | 2.28E-04 | 2.21E-04 | 1.23E-04 | 2.55E-04 | 2.63E-04 | 2.42E-04 | 2.42E-04 | 2.41E-04 | 2.38E-04 | 2.28E-04 | 2.04E-04 | 1.38E-04 | 2.27E-04 | 1.68E-04 | 1.54E-04 |
| Alanine, aspartate and glutamate metabolism | 8.54E-03 | 9.11E-03 | 9.18E-03 | 8.77E-03 | 8.93E-03 | 8.73E-03 | 9.02E-03 | 8.76E-03 | 9.08E-03 | 8.26E-03 | 8.09E-03 | 9.40E-03 | 7.82E-03 | 7.77E-03 | 7.70E-03 | 7.69E-03 | 7.83E-03 | 7.52E-03 | 9.24E-03 | 8.89E-03 | 9.67E-03 | 9.69E-03 | 9.36E-03 | 9.58E-03 |
| Aldosterone-regulated sodium reabsorption | 1.06E-06 | 7.00E-08 | 5.52E-08 | 1.46E-07 | 2.09E-07 | 9.87E-08 | 4.29E-07 | 2.49E-07 | 1.10E-07 | 6.71E-08 | 2.85E-08 | 0.00E+00 | 0.00E+00 | 0.00E+00 | 5.25E-08 | 0.00E+00 | 0.00E+00 | 0.00E+00 | 2.27E-07 | 5.87E-07 | 1.83E-06 | 1.34E-06 | 1.63E-06 | 1.52E-06 |
| Alzheimer's disease | 1.22E-03 | 1.23E-03 | 1.02E-03 | 1.02E-03 | 7.98E-04 | 1.04E-03 | 1.21E-03 | 1.11E-03 | 1.03E-03 | 6.66E-04 | 6.53E-04 | 1.14E-03 | 6.30E-04 | 5.94E-04 | 5.46E-04 | 5.20E-04 | 6.00E-04 | 4.33E-04 | 9.12E-04 | 7.62E-04 | 7.29E-04 | 8.91E-04 | 1.18E-03 | 9.29E-04 |
| Amino acid metabolism | 2.29E-03 | 1.75E-03 | 2.25E-03 | 2.04E-03 | 2.52E-03 | 2.01E-03 | 2.05E-03 | 2.45E-03 | 2.09E-03 | 2.53E-03 | 2.58E-03 | 1.78E-03 | 2.55E-03 | 2.59E-03 | 2.79E-03 | 2.78E-03 | 2.65E-03 | 3.08E-03 | 2.03E-03 | 2.44E-03 | 2.52E-03 | 2.34E-03 | 2.34E-03 | 2.62E-03 |
| Amino acid related enzymes | 1.14E-02 | 1.15E-02 | 1.18E-02 | 1.12E-02 | 1.18E-02 | 1.13E-02 | 1.19E-02 | 1.18E-02 | 1.17E-02 | 1.16E-02 | 1.17E-02 | 1.16E-02 | 1.07E-02 | 1.06E-02 | 1.07E-02 | 1.07E-02 | 1.08E-02 | 1.07E-02 | 1.27E-02 | 1.22E-02 | 1.35E-02 | 1.29E-02 | 1.31E-02 | 1.35E-02 |
| Amino sugar and nucleotide sugar metabolism | 8.45E-03 | 9.39E-03 | 1.02E-02 | 9.89E-03 | 1.08E-02 | 9.94E-03 | 9.82E-03 | 9.62E-03 | 1.03E-02 | 1.01E-02 | 1.03E-02 | 1.03E-02 | 9.74E-03 | 9.77E-03 | 9.88E-03 | 9.98E-03 | 9.90E-03 | 9.89E-03 | 9.39E-03 | 1.01E-02 | 1.07E-02 | 1.09E-02 | 9.79E-03 | 1.02E-02 |
| Aminoacyl-tRNA biosynthesis | 7.78E-03 | 7.30E-03 | 8.21E-03 | 6.74E-03 | 8.75E-03 | 6.77E-03 | 7.57E-03 | 8.01E-03 | 7.36E-03 | 7.59E-03 | 7.70E-03 | 7.28E-03 | 6.20E-03 | 6.18E-03 | 6.19E-03 | 6.14E-03 | 6.28E-03 | 6.12E-03 | 9.39E-03 | 8.50E-03 | 9.96E-03 | 9.36E-03 | 9.77E-03 | 9.71E-03 |
| Aminobenzoate degradation | 4.19E-03 | 2.82E-03 | 2.66E-03 | 2.11E-03 | 2.23E-03 | 2.02E-03 | 2.67E-03 | 3.12E-03 | 2.12E-03 | 2.69E-03 | 2.58E-03 | 2.15E-03 | 2.38E-03 | 2.49E-03 | 2.11E-03 | 2.01E-03 | 2.15E-03 | 1.93E-03 | 3.69E-03 | 3.12E-03 | 2.68E-03 | 2.76E-03 | 2.85E-03 | 2.91E-03 |
| Amoebiasis | 1.24E-04 | 6.31E-05 | 1.26E-04 | 4.46E-05 | 1.87E-04 | 4.26E-05 | 5.98E-05 | 8.83E-05 | 8.03E-05 | 1.49E-05 | 2.15E-05 | 3.67E-05 | 7.63E-06 | 5.88E-06 | 6.09E-06 | 6.37E-06 | 1.03E-05 | 4.69E-06 | 8.89E-05 | 5.75E-05 | 5.37E-05 | 1.52E-04 | 6.31E-05 | 3.54E-05 |
| Amyotrophic lateral sclerosis (ALS) | 6.71E-04 | 3.69E-04 | 2.44E-04 | 3.74E-04 | 2.10E-04 | 3.65E-04 | 3.61E-04 | 4.13E-04 | 3.35E-04 | 4.68E-04 | 4.26E-04 | 3.22E-04 | 4.47E-04 | 4.57E-04 | 4.58E-04 | 4.69E-04 | 4.45E-04 | 4.68E-04 | 3.34E-04 | 3.61E-04 | 2.75E-04 | 4.02E-04 | 3.27E-04 | 3.94E-04 |
| Antigen processing and presentation | 1.76E-04 | 2.48E-04 | 2.70E-04 | 2.45E-04 | 2.88E-04 | 2.48E-04 | 2.47E-04 | 2.39E-04 | 2.61E-04 | 2.43E-04 | 2.51E-04 | 2.55E-04 | 2.56E-04 | 2.57E-04 | 2.61E-04 | 2.63E-04 | 2.60E-04 | 2.54E-04 | 2.36E-04 | 2.44E-04 | 3.03E-04 | 1.99E-04 | 2.71E-04 | 3.05E-04 |
| Apoptosis | 3.21E-04 | 8.58E-05 | 4.63E-05 | 5.18E-05 | 5.70E-05 | 4.20E-05 | 1.08E-04 | 1.68E-04 | 5.70E-05 | 6.16E-05 | 7.58E-05 | 2.11E-05 | 7.31E-06 | 6.24E-06 | 8.14E-06 | 7.59E-06 | 1.14E-05 | 5.27E-06 | 9.28E-05 | 6.43E-05 | 3.77E-05 | 8.90E-05 | 7.73E-05 | 9.69E-05 |
| Arachidonic acid metabolism | 7.59E-04 | 6.33E-04 | 6.23E-04 | 5.67E-04 | 6.26E-04 | 5.58E-04 | 6.10E-04 | 6.59E-04 | 5.62E-04 | 5.76E-04 | 5.90E-04 | 4.80E-04 | 6.40E-04 | 6.57E-04 | 5.85E-04 | 5.61E-04 | 5.93E-04 | 5.52E-04 | 7.92E-04 | 6.82E-04 | 6.68E-04 | 5.98E-04 | 7.54E-04 | 7.33E-04 |
| Arginine and proline metabolism | 1.21E-02 | 1.12E-02 | 1.17E-02 | 1.08E-02 | 1.22E-02 | 1.07E-02 | 1.14E-02 | 1.19E-02 | 1.11E-02 | 1.03E-02 | 1.00E-02 | 1.05E-02 | 1.06E-02 | 1.07E-02 | 1.01E-02 | 9.94E-03 | 1.03E-02 | 9.81E-03 | 1.19E-02 | 1.16E-02 | 1.14E-02 | 1.22E-02 | 1.19E-02 | 1.17E-02 |
| Arrhythmogenic right ventricular cardiomyopathy (ARVC) | 0.00E+00 | 0.00E+00 | 0.00E+00 | 1.46E-07 | 4.17E-08 | 3.29E-08 | 0.00E+00 | 9.96E-08 | 0.00E+00 | 0.00E+00 | 0.00E+00 | 0.00E+00 | 0.00E+00 | 0.00E+00 | 0.00E+00 | 3.60E-08 | 0.00E+00 | 0.00E+00 | 4.55E-08 | 0.00E+00 | 0.00E+00 | 5.15E-07 | 1.37E-06 | 1.83E-07 |
| Ascorbate and aldarate metabolism | 1.99E-03 | 1.66E-03 | 1.53E-03 | 1.98E-03 | 1.47E-03 | 2.00E-03 | 1.73E-03 | 1.77E-03 | 1.77E-03 | 2.10E-03 | 2.16E-03 | 1.44E-03 | 2.24E-03 | 2.20E-03 | 2.42E-03 | 2.39E-03 | 2.32E-03 | 2.60E-03 | 1.60E-03 | 1.94E-03 | 1.79E-03 | 1.89E-03 | 1.51E-03 | 1.73E-03 |
| Atrazine degradation | 7.78E-04 | 4.64E-04 | 4.77E-04 | 4.09E-04 | 6.71E-04 | 3.60E-04 | 4.47E-04 | 5.54E-04 | 4.05E-04 | 2.75E-04 | 2.50E-04 | 2.55E-04 | 3.30E-04 | 3.49E-04 | 2.64E-04 | 2.32E-04 | 2.87E-04 | 2.24E-04 | 3.96E-04 | 3.74E-04 | 2.95E-04 | 3.02E-04 | 4.65E-04 | 2.83E-04 |
| Bacterial chemotaxis | 7.22E-03 | 9.62E-03 | 8.27E-03 | 9.10E-03 | 5.65E-03 | 9.25E-03 | 8.54E-03 | 6.78E-03 | 8.76E-03 | 5.25E-03 | 5.39E-03 | 8.27E-03 | 6.54E-03 | 6.27E-03 | 6.07E-03 | 5.95E-03 | 6.43E-03 | 5.40E-03 | 5.96E-03 | 6.06E-03 | 6.51E-03 | 5.40E-03 | 7.13E-03 | 6.66E-03 |
| Bacterial invasion of epithelial cells | 7.94E-05 | 2.90E-05 | 7.57E-05 | 9.31E-05 | 1.70E-04 | 8.95E-05 | 5.78E-05 | 1.02E-04 | 7.99E-05 | 1.98E-04 | 2.10E-04 | 5.08E-05 | 2.05E-04 | 2.17E-04 | 2.36E-04 | 2.47E-04 | 2.13E-04 | 2.79E-04 | 3.02E-05 | 1.09E-04 | 7.64E-05 | 6.11E-05 | 3.70E-05 | 7.90E-05 |
| Bacterial motility proteins | 1.96E-02 | 2.47E-02 | 2.17E-02 | 2.38E-02 | 1.73E-02 | 2.43E-02 | 2.21E-02 | 1.89E-02 | 2.21E-02 | 1.64E-02 | 1.66E-02 | 2.12E-02 | 1.98E-02 | 1.94E-02 | 1.92E-02 | 1.91E-02 | 1.97E-02 | 1.85E-02 | 1.76E-02 | 1.73E-02 | 1.69E-02 | 1.48E-02 | 2.05E-02 | 1.68E-02 |
| Bacterial secretion system | 8.85E-03 | 9.04E-03 | 7.74E-03 | 9.19E-03 | 6.62E-03 | 9.23E-03 | 8.55E-03 | 7.99E-03 | 8.61E-03 | 8.76E-03 | 8.64E-03 | 1.01E-02 | 9.87E-03 | 9.91E-03 | 9.56E-03 | 9.66E-03 | 9.56E-03 | 9.38E-03 | 7.92E-03 | 7.98E-03 | 7.18E-03 | 7.60E-03 | 8.35E-03 | 7.28E-03 |
| Bacterial toxins | 7.69E-04 | 9.22E-04 | 8.72E-04 | 7.93E-04 | 7.34E-04 | 8.29E-04 | 7.58E-04 | 6.16E-04 | 7.98E-04 | 8.72E-04 | 8.12E-04 | 1.26E-03 | 6.81E-04 | 6.61E-04 | 6.38E-04 | 5.93E-04 | 6.17E-04 | 6.76E-04 | 6.41E-04 | 6.71E-04 | 6.07E-04 | 6.72E-04 | 5.63E-04 | 5.98E-04 |
| Basal transcription factors | 5.08E-05 | 1.39E-05 | 1.92E-05 | 1.10E-05 | 1.72E-05 | 9.18E-06 | 2.50E-05 | 2.86E-05 | 1.12E-05 | 4.07E-05 | 1.14E-05 | 1.30E-05 | 3.78E-06 | 4.14E-06 | 4.78E-06 | 2.59E-06 | 6.39E-06 | 3.81E-06 | 6.54E-05 | 5.55E-05 | 1.42E-04 | 1.47E-04 | 5.15E-05 | 1.07E-04 |
| Base excision repair | 3.84E-03 | 3.31E-03 | 3.59E-03 | 3.31E-03 | 3.47E-03 | 3.27E-03 | 3.36E-03 | 3.48E-03 | 3.31E-03 | 3.74E-03 | 3.70E-03 | 3.44E-03 | 3.36E-03 | 3.37E-03 | 3.40E-03 | 3.41E-03 | 3.40E-03 | 3.43E-03 | 4.13E-03 | 4.02E-03 | 4.32E-03 | 4.74E-03 | 3.91E-03 | 4.15E-03 |
| Benzoate degradation | 5.59E-03 | 4.20E-03 | 3.52E-03 | 3.27E-03 | 2.60E-03 | 3.13E-03 | 3.91E-03 | 4.17E-03 | 3.22E-03 | 3.50E-03 | 3.47E-03 | 3.18E-03 | 3.41E-03 | 3.49E-03 | 3.05E-03 | 2.87E-03 | 3.10E-03 | 2.82E-03 | 4.87E-03 | 4.27E-03 | 3.52E-03 | 3.77E-03 | 3.65E-03 | 3.72E-03 |
| Betalain biosynthesis | 4.28E-05 | 9.47E-06 | 7.53E-06 | 1.79E-05 | 1.26E-05 | 1.17E-05 | 2.34E-05 | 5.11E-05 | 1.27E-05 | 6.34E-06 | 6.49E-06 | 3.09E-06 | 3.71E-05 | 4.32E-05 | 2.30E-05 | 2.02E-05 | 2.37E-05 | 1.62E-05 | 7.86E-06 | 1.76E-05 | 3.02E-05 | 2.07E-05 | 1.70E-05 | 3.17E-05 |
| Bile secretion | 2.96E-05 | 9.12E-05 | 9.82E-05 | 8.24E-05 | 4.20E-05 | 8.96E-05 | 9.22E-05 | 4.49E-05 | 8.21E-05 | 1.65E-05 | 1.79E-05 | 7.84E-05 | 2.35E-05 | 1.29E-05 | 2.14E-05 | 2.00E-05 | 2.58E-05 | 1.33E-05 | 5.90E-05 | 3.61E-05 | 3.07E-05 | 4.50E-05 | 4.73E-05 | 2.92E-05 |

| | | | | | | | | | | | | | | | | | | | | | | | | |
|---|---|---|---|---|---|---|---|---|---|---|---|---|---|---|---|---|---|---|---|---|---|---|---|---|
| Biosynthesis and biodegradation of secondary metabolites | 1.26E-03 | 8.78E-04 | 8.45E-04 | 1.23E-03 | 8.80E-04 | 1.19E-03 | 1.07E-03 | 1.24E-03 | 1.06E-03 | 1.35E-03 | 1.35E-03 | 8.59E-04 | 1.71E-03 | 1.75E-03 | 1.77E-03 | 1.76E-03 | 1.70E-03 | 1.87E-03 | 7.87E-04 | 1.19E-03 | 1.02E-03 | 8.69E-04 | 8.45E-04 | 9.86E-04 |
| Biosynthesis of 12-, 14- and 16-membered macrolides | 3.57E-06 | 2.68E-06 | 1.13E-06 | 4.39E-07 | 1.00E-06 | 6.25E-07 | 1.57E-06 | 7.47E-07 | 9.17E-07 | 8.72E-07 | 1.14E-06 | 1.37E-06 | 1.89E-07 | 1.80E-07 | 3.15E-07 | 1.80E-07 | 3.68E-07 | 2.34E-07 | 1.91E-06 | 5.87E-07 | 6.64E-07 | 4.43E-06 | 2.64E-06 | 7.31E-07 |
| Biosynthesis of ansamycins | 5.39E-04 | 4.77E-04 | 4.88E-04 | 5.28E-04 | 4.74E-04 | 5.11E-04 | 5.06E-04 | 5.17E-04 | 5.06E-04 | 5.00E-04 | 5.45E-04 | 5.09E-04 | 5.70E-04 | 5.68E-04 | 5.78E-04 | 5.76E-04 | 5.83E-04 | 5.72E-04 | 4.93E-04 | 5.46E-04 | 5.18E-04 | 5.22E-04 | 5.07E-04 | 5.64E-04 |
| Biosynthesis of siderophore group nonribosomal peptides | 7.72E-04 | 8.53E-04 | 8.05E-04 | 1.02E-03 | 5.94E-04 | 1.02E-03 | 8.27E-04 | 7.46E-04 | 8.92E-04 | 1.16E-03 | 1.12E-03 | 7.66E-04 | 1.26E-03 | 1.25E-03 | 1.29E-03 | 1.30E-03 | 1.24E-03 | 1.36E-03 | 6.98E-04 | 8.22E-04 | 5.56E-04 | 6.20E-04 | 4.53E-04 | 5.23E-04 |
| Biosynthesis of type II polyketide backbone | 2.35E-06 | 1.40E-07 | 8.55E-07 | 0.00E+00 | 0.00E+00 | 4.28E-07 | 0.00E+00 | 2.49E-07 | 0.00E+00 | 0.00E+00 | 5.12E-07 | 1.72E-07 | 0.00E+00 | 3.00E-07 | 2.63E-07 | 1.08E-07 | 4.60E-08 | 8.20E-07 | 3.00E-06 | 0.00E+00 | 1.83E-06 | 5.15E-07 | 1.30E-06 | 4.87E-07 |
| Biosynthesis of type II polyketide products | 8.01E-06 | 7.58E-06 | 4.66E-06 | 1.01E-05 | 2.54E-06 | 6.75E-06 | 4.19E-06 | 7.37E-06 | 4.95E-06 | 7.14E-06 | 5.95E-06 | 2.23E-06 | 3.74E-05 | 4.30E-05 | 2.29E-05 | 1.98E-05 | 2.30E-05 | 1.58E-05 | 1.31E-05 | 1.47E-05 | 8.31E-06 | 5.46E-06 | 6.97E-06 | 7.80E-06 |
| Biosynthesis of unsaturated fatty acids | 2.88E-03 | 3.59E-03 | 3.11E-03 | 3.25E-03 | 2.61E-03 | 3.26E-03 | 3.25E-03 | 3.04E-03 | 3.00E-03 | 3.08E-03 | 3.00E-03 | 3.77E-03 | 3.15E-03 | 3.13E-03 | 3.03E-03 | 3.01E-03 | 3.07E-03 | 2.93E-03 | 3.61E-03 | 3.17E-03 | 3.14E-03 | 2.96E-03 | 3.14E-03 | 3.17E-03 |
| Biosynthesis of vancomycin group antibiotics | 3.76E-04 | 2.65E-04 | 3.66E-04 | 2.71E-04 | 5.01E-04 | 2.73E-04 | 3.41E-04 | 3.80E-04 | 4.06E-04 | 2.81E-04 | 2.88E-04 | 2.58E-04 | 2.31E-04 | 2.28E-04 | 2.63E-04 | 2.60E-04 | 2.57E-04 | 2.96E-04 | 3.73E-04 | 4.26E-04 | 5.34E-04 | 4.31E-04 | 4.57E-04 | 6.00E-04 |
| Biotin metabolism | 1.40E-03 | 1.42E-03 | 1.50E-03 | 1.36E-03 | 1.52E-03 | 1.35E-03 | 1.39E-03 | 1.42E-03 | 1.36E-03 | 1.58E-03 | 1.46E-03 | 1.54E-03 | 1.41E-03 | 1.42E-03 | 1.46E-03 | 1.48E-03 | 1.45E-03 | 1.50E-03 | 1.56E-03 | 1.63E-03 | 2.01E-03 | 1.35E-03 | 1.80E-03 | 2.24E-03 |
| Bisphenol degradation | 1.12E-03 | 8.83E-04 | 8.34E-04 | 6.64E-04 | 6.01E-04 | 6.50E-04 | 7.94E-04 | 8.59E-04 | 6.38E-04 | 7.58E-04 | 7.35E-04 | 6.33E-04 | 6.16E-04 | 6.42E-04 | 4.95E-04 | 4.63E-04 | 5.28E-04 | 4.00E-04 | 1.13E-03 | 8.12E-04 | 6.64E-04 | 7.66E-04 | 7.65E-04 | 9.19E-04 |
| Bladder cancer | 1.68E-04 | 1.71E-04 | 1.42E-04 | 1.94E-04 | 9.58E-05 | 1.94E-04 | 1.80E-04 | 1.61E-04 | 1.63E-04 | 1.85E-04 | 1.56E-04 | 2.24E-04 | 1.81E-04 | 1.75E-04 | 1.96E-04 | 2.04E-04 | 1.94E-04 | 2.06E-04 | 1.28E-04 | 1.41E-04 | 1.01E-04 | 2.23E-04 | 1.06E-04 | 1.24E-04 |
| Butanoate metabolism | 1.07E-02 | 1.00E-02 | 9.19E-03 | 9.25E-03 | 7.14E-03 | 9.15E-03 | 9.71E-03 | 9.49E-03 | 8.97E-03 | 9.00E-03 | 8.89E-03 | 9.87E-03 | 8.81E-03 | 8.79E-03 | 8.70E-03 | 8.63E-03 | 8.76E-03 | 8.53E-03 | 1.04E-02 | 9.60E-03 | 8.50E-03 | 9.44E-03 | 9.29E-03 | 8.70E-03 |
| Butirosin and neomycin biosynthesis | 2.42E-04 | 1.45E-04 | 2.91E-04 | 1.49E-04 | 4.45E-04 | 1.45E-04 | 2.15E-04 | 2.77E-04 | 2.45E-04 | 1.64E-04 | 1.73E-04 | 1.09E-04 | 1.13E-04 | 1.15E-04 | 1.07E-04 | 1.00E-04 | 1.16E-04 | 8.78E-05 | 3.34E-04 | 3.12E-04 | 3.38E-04 | 5.26E-04 | 3.41E-04 | 3.40E-04 |
| C5-Branched dibasic acid metabolism | 2.72E-03 | 2.57E-03 | 2.64E-03 | 2.62E-03 | 2.61E-03 | 2.60E-03 | 2.76E-03 | 2.91E-03 | 2.69E-03 | 2.92E-03 | 2.85E-03 | 2.59E-03 | 2.67E-03 | 2.68E-03 | 2.75E-03 | 2.78E-03 | 2.74E-03 | 2.78E-03 | 3.03E-03 | 2.80E-03 | 2.66E-03 | 2.99E-03 | 2.85E-03 | 2.66E-03 |
| CAM ligands | 1.63E-08 | 0.00E+00 | 0.00E+00 | 2.93E-07 | 9.60E-07 | 1.88E-06 | 1.81E-06 | 1.84E-06 | 6.97E-07 | 0.00E+00 | 0.00E+00 | 0.00E+00 | 0.00E+00 | 6.00E-08 | 5.25E-08 | 0.00E+00 | 0.00E+00 | 0.00E+00 | 4.55E-08 | 0.00E+00 | 1.66E-07 | 0.00E+00 | 5.42E-07 | 6.09E-07 |
| Caffeine metabolism | 1.99E-05 | 5.34E-06 | 1.37E-05 | 2.34E-06 | 1.71E-06 | 1.12E-06 | 2.33E-06 | 3.89E-06 | 9.17E-07 | 2.78E-06 | 2.33E-06 | 7.55E-06 | 3.15E-07 | 3.00E-07 | 4.73E-07 | 2.88E-07 | 5.98E-07 | 1.76E-07 | 1.39E-05 | 2.03E-05 | 7.81E-06 | 6.08E-06 | 5.31E-06 | 2.92E-06 |
| Calcium signaling pathway | 9.05E-06 | 1.67E-05 | 6.76E-05 | 1.26E-05 | 1.49E-04 | 1.44E-05 | 1.99E-05 | 3.56E-05 | 2.90E-05 | 2.98E-06 | 2.25E-06 | 4.35E-06 | 2.21E-06 | 1.92E-06 | 1.63E-06 | 1.37E-06 | 2.76E-06 | 1.52E-06 | 4.73E-06 | 1.12E-05 | 1.03E-05 | 9.28E-06 | 1.07E-05 | 4.33E-06 |
| Caprolactam degradation | 2.92E-03 | 2.65E-03 | 2.18E-03 | 2.16E-03 | 1.35E-03 | 2.11E-03 | 2.37E-03 | 2.36E-03 | 1.91E-03 | 2.18E-03 | 2.05E-03 | 2.65E-03 | 2.14E-03 | 2.16E-03 | 2.03E-03 | 1.99E-03 | 2.05E-03 | 1.92E-03 | 2.72E-03 | 2.38E-03 | 1.78E-03 | 2.03E-03 | 2.18E-03 | 1.82E-03 |
| Carbohydrate digestion and absorption | 1.58E-04 | 2.94E-04 | 2.92E-04 | 3.70E-04 | 3.14E-04 | 4.08E-04 | 3.49E-04 | 2.50E-04 | 3.76E-04 | 2.62E-04 | 2.62E-04 | 3.11E-04 | 2.76E-04 | 2.54E-04 | 3.04E-04 | 3.01E-04 | 2.96E-04 | 3.16E-04 | 1.56E-04 | 1.78E-04 | 1.48E-04 | 1.57E-04 | 1.20E-04 | 1.55E-04 |
| Carbohydrate metabolism | 1.35E-03 | 9.78E-04 | 1.27E-03 | 1.23E-03 | 1.87E-03 | 1.23E-03 | 1.13E-03 | 1.34E-03 | 1.44E-03 | 1.59E-03 | 1.60E-03 | 1.17E-03 | 1.59E-03 | 1.57E-03 | 1.77E-03 | 1.71E-03 | 1.65E-03 | 2.04E-03 | 9.11E-04 | 1.36E-03 | 1.25E-03 | 1.29E-03 | 1.02E-03 | 1.14E-03 |
| Carbon fixation in photosynthetic organisms | 4.43E-03 | 4.36E-03 | 5.05E-03 | 4.47E-03 | 5.89E-03 | 4.49E-03 | 4.60E-03 | 4.84E-03 | 4.70E-03 | 4.41E-03 | 4.52E-03 | 4.47E-03 | 4.38E-03 | 4.36E-03 | 4.45E-03 | 4.49E-03 | 4.46E-03 | 4.47E-03 | 4.47E-03 | 4.65E-03 | 4.61E-03 | 4.66E-03 | 5.08E-03 | 4.93E-03 |
| Carbon fixation pathways in prokaryotes | 9.87E-03 | 9.94E-03 | 9.74E-03 | 9.51E-03 | 8.62E-03 | 9.46E-03 | 9.87E-03 | 9.87E-03 | 9.72E-03 | 9.78E-03 | 9.51E-03 | 1.02E-02 | 9.17E-03 | 9.15E-03 | 9.14E-03 | 9.16E-03 | 9.20E-03 | 9.02E-03 | 1.09E-02 | 1.01E-02 | 1.02E-02 | 9.82E-03 | 1.10E-02 | 1.07E-02 |
| Cardiac muscle contraction | 5.14E-04 | 5.94E-04 | 4.23E-04 | 4.51E-04 | 2.58E-04 | 4.61E-04 | 5.17E-04 | 4.04E-04 | 4.30E-04 | 1.83E-04 | 1.73E-04 | 5.73E-04 | 2.42E-04 | 2.25E-04 | 1.71E-04 | 1.52E-04 | 2.02E-04 | 9.76E-05 | 3.27E-04 | 2.52E-04 | 1.90E-04 | 2.83E-04 | 5.29E-04 | 2.75E-04 |
| Carotenoid biosynthesis | 4.39E-04 | 2.11E-04 | 6.22E-04 | 1.64E-04 | 1.27E-03 | 1.65E-04 | 2.41E-04 | 4.33E-04 | 3.07E-04 | 8.89E-05 | 7.93E-05 | 8.25E-05 | 7.45E-05 | 7.43E-05 | 5.56E-05 | 5.29E-05 | 7.07E-05 | 4.27E-05 | 2.24E-04 | 2.42E-04 | 2.22E-04 | 1.91E-04 | 3.32E-04 | 2.83E-04 |
| Cell cycle | 8.32E-07 | 2.33E-08 | 5.52E-08 | 1.46E-07 | 0.00E+00 | 0.00E+00 | 0.00E+00 | 4.98E-08 | 0.00E+00 | 0.00E+00 | 0.00E+00 | 0.00E+00 | 0.00E+00 | 0.00E+00 | 0.00E+00 | 0.00E+00 | 0.00E+00 | 0.00E+00 | 0.00E+00 | 0.00E+00 | 0.00E+00 | 0.00E+00 | 0.00E+00 | 0.00E+00 |
| Cell cycle - Caulobacter | 4.26E-03 | 3.86E-03 | 4.14E-03 | 3.48E-03 | 4.44E-03 | 3.51E-03 | 3.90E-03 | 4.11E-03 | 3.76E-03 | 3.38E-03 | 3.46E-03 | 3.56E-03 | 3.03E-03 | 3.02E-03 | 2.96E-03 | 2.94E-03 | 3.03E-03 | 2.84E-03 | 4.27E-03 | 4.02E-03 | 4.88E-03 | 4.24E-03 | 4.69E-03 | 4.85E-03 |
| Cell cycle - yeast | 0.00E+00 | 0.00E+00 | 0.00E+00 | 0.00E+00 | 0.00E+00 | 0.00E+00 | 0.00E+00 | 0.00E+00 | 0.00E+00 | 0.00E+00 | 0.00E+00 | 0.00E+00 | 0.00E+00 | 0.00E+00 | 0.00E+00 | 0.00E+00 | 0.00E+00 | 0.00E+00 | 0.00E+00 | 0.00E+00 | 0.00E+00 | 0.00E+00 | 0.00E+00 | 0.00E+00 |
| Cell division | 8.48E-04 | 7.61E-04 | 7.42E-04 | 7.58E-04 | 7.30E-04 | 7.59E-04 | 7.86E-04 | 8.10E-04 | 7.91E-04 | 8.15E-04 | 7.90E-04 | 6.70E-04 | 8.00E-04 | 8.01E-04 | 8.10E-04 | 8.10E-04 | 8.02E-04 | 8.24E-04 | 7.72E-04 | 7.66E-04 | 6.89E-04 | 7.22E-04 | 8.72E-04 | 7.19E-04 |
| Cell motility and secretion | 2.62E-03 | 3.09E-03 | 2.80E-03 | 2.95E-03 | 2.79E-03 | 3.00E-03 | 3.03E-03 | 2.91E-03 | 2.98E-03 | 2.64E-03 | 2.61E-03 | 2.61E-03 | 2.85E-03 | 2.85E-03 | 2.75E-03 | 2.73E-03 | 2.78E-03 | 2.66E-03 | 2.94E-03 | 2.84E-03 | 3.46E-03 | 2.56E-03 | 3.47E-03 | 3.38E-03 |
| Cellular antigens | 6.98E-04 | 6.69E-04 | 6.32E-04 | 5.67E-04 | 6.51E-04 | 5.73E-04 | 6.54E-04 | 6.60E-04 | 6.14E-04 | 4.26E-04 | 4.28E-04 | 6.88E-04 | 5.14E-04 | 5.14E-04 | 4.70E-04 | 4.60E-04 | 4.85E-04 | 4.18E-04 | 6.52E-04 | 6.17E-04 | 5.62E-04 | 6.08E-04 | 7.56E-04 | 8.57E-04 |
| Chagas disease (American trypanosomiasis) | 1.60E-04 | 1.44E-04 | 1.13E-04 | 1.99E-04 | 1.00E-04 | 1.98E-04 | 1.60E-04 | 1.37E-04 | 1.78E-04 | 1.93E-04 | 1.87E-04 | 1.26E-04 | 2.16E-04 | 2.19E-04 | 2.18E-04 | 2.22E-04 | 2.16E-04 | 2.22E-04 | 1.53E-04 | 1.77E-04 | 1.19E-04 | 2.12E-04 | 1.56E-04 | 1.42E-04 |
| Chaperones and folding catalysts | 9.22E-03 | 1.07E-02 | 1.04E-02 | 1.04E-02 | 1.06E-02 | 1.05E-02 | 1.03E-02 | 9.90E-03 | 1.04E-02 | 1.05E-02 | 1.05E-02 | 1.08E-02 | 1.03E-02 | 1.02E-02 | 1.03E-02 | 1.04E-02 | 1.03E-02 | 1.03E-02 | 1.02E-02 | 9.82E-03 | 9.93E-03 | 9.56E-03 | 1.07E-02 | 9.84E-03 |
| Chloroalkane and chloroalkene degradation | 2.81E-03 | 2.47E-03 | 2.31E-03 | 2.10E-03 | 2.40E-03 | 2.11E-03 | 2.36E-03 | 2.38E-03 | 2.28E-03 | 1.97E-03 | 2.01E-03 | 2.07E-03 | 1.78E-03 | 1.77E-03 | 1.65E-03 | 1.59E-03 | 1.71E-03 | 1.51E-03 | 2.78E-03 | 2.30E-03 | 2.58E-03 | 2.29E-03 | 2.38E-03 | 2.64E-03 |
| Chlorocyclohexane and chlorobenzene degradation | 8.44E-04 | 4.81E-04 | 4.96E-04 | 3.67E-04 | 6.90E-04 | 3.31E-04 | 4.43E-04 | 6.67E-04 | 3.87E-04 | 4.80E-04 | 4.63E-04 | 2.17E-04 | 5.19E-04 | 5.58E-04 | 4.56E-04 | 4.28E-04 | 4.53E-04 | 4.27E-04 | 6.57E-04 | 5.83E-04 | 4.78E-04 | 5.23E-04 | 4.88E-04 | 4.60E-04 |
| Cholinergic synapse | 0.00E+00 | 0.00E+00 | 0.00E+00 | 0.00E+00 | 0.00E+00 | 0.00E+00 | 0.00E+00 | 0.00E+00 | 0.00E+00 | 0.00E+00 | 0.00E+00 | 0.00E+00 | 0.00E+00 | 0.00E+00 | 0.00E+00 | 0.00E+00 | 0.00E+00 | 0.00E+00 | 0.00E+00 | 0.00E+00 | 0.00E+00 | 0.00E+00 | 0.00E+00 | 0.00E+00 |
| Chromosome | 1.26E-02 | 1.31E-02 | 1.30E-02 | 1.28E-02 | 1.40E-02 | 1.29E-02 | 1.30E-02 | 1.30E-02 | 1.29E-02 | 1.32E-02 | 1.35E-02 | 1.35E-02 | 1.27E-02 | 1.26E-02 | 1.28E-02 | 1.29E-02 | 1.28E-02 | 1.28E-02 | 1.33E-02 | 1.30E-02 | 1.39E-02 | 1.30E-02 | 1.42E-02 | 1.38E-02 |
| Chronic myeloid leukemia | 1.31E-07 | 3.27E-07 | 7.73E-07 | 2.93E-07 | 1.25E-07 | 1.97E-07 | 7.62E-07 | 3.99E-07 | 2.20E-07 | 3.35E-08 | 5.69E-08 | 6.87E-07 | 2.52E-07 | 1.20E-07 | 4.73E-07 | 2.16E-07 | 2.30E-07 | 0.00E+00 | 7.27E-07 | 8.80E-07 | 0.00E+00 | 6.19E-07 | 3.97E-07 | 3.05E-07 |
| Circadian rhythm - plant | 3.95E-05 | 1.22E-05 | 3.33E-05 | 9.07E-06 | 1.03E-05 | 5.96E-06 | 7.86E-06 | 1.31E-05 | 4.92E-06 | 6.44E-06 | 5.75E-06 | 1.88E-05 | 3.03E-06 | 2.16E-06 | 2.26E-06 | 2.48E-06 | 3.31E-06 | 1.82E-06 | 5.97E-05 | 4.73E-05 | 3.12E-05 | 1.23E-04 | 6.57E-05 | 3.58E-05 |
| Citrate cycle (TCA cycle) | 6.73E-03 | 6.47E-03 | 6.40E-03 | 6.35E-03 | 5.42E-03 | 6.29E-03 | 6.54E-03 | 6.51E-03 | 6.55E-03 | 7.03E-03 | 6.55E-03 | 6.42E-03 | 6.35E-03 | 6.34E-03 | 6.43E-03 | 6.47E-03 | 6.43E-03 | 6.43E-03 | 7.69E-03 | 7.17E-03 | 7.06E-03 | 8.25E-03 | 7.73E-03 | 7.50E-03 |
| Clavulanic acid biosynthesis | 5.14E-06 | 5.13E-07 | 1.93E-07 | 1.46E-07 | 1.25E-07 | 2.30E-07 | 3.33E-07 | 1.49E-07 | 0.00E+00 | 4.02E-07 | 2.28E-07 | 1.14E-07 | 0.00E+00 | 0.00E+00 | 0.00E+00 | 0.00E+00 | 9.20E-08 | 5.86E-08 | 5.00E-07 | 8.80E-07 | 3.32E-07 | 2.06E-07 | 1.81E-07 | 2.44E-07 |
| Colorectal cancer | 2.58E-04 | 4.24E-05 | 3.26E-05 | 4.02E-05 | 5.04E-05 | 2.92E-05 | 9.31E-05 | 1.59E-04 | 4.60E-05 | 1.30E-05 | 1.16E-05 | 1.22E-05 | 2.96E-06 | 4.02E-06 | 4.15E-06 | 3.89E-06 | 6.02E-06 | 4.10E-06 | 3.49E-05 | 4.70E-05 | 2.06E-05 | 7.40E-05 | 6.78E-05 | 4.50E-05 |
| Complement and coagulation cascades | 0.00E+00 | 0.00E+00 | 0.00E+00 | 0.00E+00 | 0.00E+00 | 0.00E+00 | 0.00E+00 | 0.00E+00 | 0.00E+00 | 0.00E+00 | 0.00E+00 | 0.00E+00 | 0.00E+00 | 0.00E+00 | 0.00E+00 | 0.00E+00 | 0.00E+00 | 0.00E+00 | 0.00E+00 | 0.00E+00 | 0.00E+00 | 0.00E+00 | 0.00E+00 | 0.00E+00 |
| Cyanoamino acid metabolism | 1.91E-03 | 1.60E-03 | 1.80E-03 | 1.60E-03 | 2.09E-03 | 1.55E-03 | 1.73E-03 | 1.83E-03 | 1.86E-03 | 1.51E-03 | 1.58E-03 | 1.52E-03 | 1.66E-03 | 1.68E-03 | 1.56E-03 | 1.51E-03 | 1.58E-03 | 1.48E-03 | 1.91E-03 | 1.96E-03 | 1.76E-03 | 2.02E-03 | 1.82E-03 | 1.97E-03 |
| Cysteine and methionine metabolism | 7.29E-03 | 7.29E-03 | 7.60E-03 | 7.51E-03 | 7.65E-03 | 7.49E-03 | 7.60E-03 | 7.77E-03 | 7.61E-03 | 7.62E-03 | 7.69E-03 | 7.44E-03 | 7.32E-03 | 7.25E-03 | 7.42E-03 | 7.40E-03 | 7.45E-03 | 7.42E-03 | 7.96E-03 | 8.09E-03 | 9.21E-03 | 8.36E-03 | 8.45E-03 | 8.91E-03 |
| Cytochrome P450 | 2.04E-06 | 1.63E-07 | 2.76E-07 | 2.93E-07 | 1.25E-07 | 3.29E-08 | 1.43E-07 | 1.49E-07 | 3.67E-08 | 2.21E-06 | 4.27E-07 | 0.00E+00 | 0.00E+00 | 0.00E+00 | 0.00E+00 | 7.20E-08 | 0.00E+00 | 5.86E-08 | 5.91E-07 | 0.00E+00 | 3.32E-07 | 6.19E-07 | 1.48E-06 | 4.87E-07 |
| Cytokine receptors | 1.63E-08 | 0.00E+00 | 0.00E+00 | 0.00E+00 | 0.00E+00 | 3.29E-08 | 0.00E+00 | 0.00E+00 | 0.00E+00 | 0.00E+00 | 0.00E+00 | 0.00E+00 | 0.00E+00 | 0.00E+00 | 0.00E+00 | 0.00E+00 | 0.00E+00 | 0.00E+00 | 9.09E-08 | 0.00E+00 | 0.00E+00 | 0.00E+00 | 0.00E+00 | 0.00E+00 |
| Cytokine-cytokine receptor interaction | 1.63E-08 | 0.00E+00 | 0.00E+00 | 0.00E+00 | 0.00E+00 | 3.29E-08 | 0.00E+00 | 0.00E+00 | 0.00E+00 | 0.00E+00 | 0.00E+00 | 0.00E+00 | 0.00E+00 | 0.00E+00 | 0.00E+00 | 0.00E+00 | 0.00E+00 | 0.00E+00 | 9.09E-08 | 0.00E+00 | 0.00E+00 | 0.00E+00 | 0.00E+00 | 0.00E+00 |
| Cytoskeleton proteins | 2.09E-03 | 2.14E-03 | 2.20E-03 | 1.74E-03 | 2.68E-03 | 1.77E-03 | 1.98E-03 | 1.96E-03 | 1.91E-03 | 1.69E-03 | 1.70E-03 | 2.24E-03 | 1.27E-03 | 1.22E-03 | 1.23E-03 | 1.21E-03 | 1.29E-03 | 1.15E-03 | 2.16E-03 | 1.82E-03 | 1.94E-03 | 2.02E-03 | 2.22E-03 | 1.93E-03 |
| Cytosolic DNA-sensing pathway | 0.00E+00 | 0.00E+00 | 0.00E+00 | 0.00E+00 | 0.00E+00 | 0.00E+00 | 0.00E+00 | 0.00E+00 | 0.00E+00 | 0.00E+00 | 0.00E+00 | 0.00E+00 | 0.00E+00 | 0.00E+00 | 0.00E+00 | 0.00E+00 | 0.00E+00 | 0.00E+00 | 0.00E+00 | 0.00E+00 | 0.00E+00 | 0.00E+00 | 0.00E+00 | 0.00E+00 |
| D-Alanine metabolism | 8.21E-04 | 7.80E-04 | 8.11E-04 | 8.17E-04 | 7.69E-04 | 8.17E-04 | 8.30E-04 | 8.58E-04 | 8.02E-04 | 9.71E-04 | 9.85E-04 | 8.08E-04 | 9.06E-04 | 9.26E-04 | 9.11E-04 | 9.24E-04 | 9.10E-04 | 9.23E-04 | 9.34E-04 | 9.30E-04 | 9.54E-04 | 1.10E-03 | 9.71E-04 | 9.29E-04 |
| D-Arginine and D-ornithine metabolism | 9.93E-05 | 4.31E-05 | 5.25E-05 | 3.12E-05 | 2.91E-05 | 2.97E-05 | 7.05E-05 | 7.83E-05 | 3.86E-05 | 1.49E-05 | 1.92E-05 | 1.15E-05 | 1.05E-05 | 8.10E-06 | 1.05E-05 | 9.39E-06 | 1.30E-05 | 6.97E-06 | 4.67E-05 | 4.52E-05 | 4.25E-05 | 5.68E-05 | 1.24E-04 | 7.75E-05 |
| D-Glutamine and D-glutamate metabolism | 1.10E-03 | 1.10E-03 | 1.13E-03 | 1.01E-03 | 1.19E-03 | 1.01E-03 | 1.04E-03 | 1.12E-03 | 1.04E-03 | 1.24E-03 | 1.24E-03 | 1.17E-03 | 1.06E-03 | 1.06E-03 | 1.06E-03 | 1.07E-03 | 1.06E-03 | 1.07E-03 | 1.30E-03 | 1.21E-03 | 1.34E-03 | 1.22E-03 | 1.19E-03 | 1.34E-03 |
| DNA repair and recombination proteins | 2.11E-02 | 2.16E-02 | 2.26E-02 | 2.14E-02 | 2.33E-02 | 2.15E-02 | 2.18E-02 | 2.19E-02 | 2.20E-02 | 2.32E-02 | 2.33E-02 | 2.26E-02 | 2.12E-02 | 2.12E-02 | 2.14E-02 | 2.15E-02 | 2.14E-02 | 2.14E-02 | 2.43E-02 | 2.35E-02 | 2.56E-02 | 2.52E-02 | 2.48E-02 | 2.53E-02 |
| DNA replication | 4.29E-03 | 4.52E-03 | 4.72E-03 | 4.55E-03 | 4.78E-03 | 4.59E-03 | 4.74E-03 | 4.63E-03 | 4.79E-03 | 4.75E-03 | 4.87E-03 | 4.52E-03 | 4.29E-03 | 4.28E-03 | 4.30E-03 | 4.34E-03 | 4.34E-03 | 4.27E-03 | 5.13E-03 | 4.87E-03 | 5.27E-03 | 5.45E-03 | 5.25E-03 | 5.47E-03 |
| DNA replication proteins | 7.68E-03 | 7.90E-03 | 8.17E-03 | 8.07E-03 | 8.70E-03 | 8.10E-03 | 8.25E-03 | 8.30E-03 | 8.38E-03 | 8.62E-03 | 8.93E-03 | 8.15E-03 | 8.01E-03 | 8.00E-03 | 8.13E-03 | 8.23E-03 | 8.14E-03 | 8.18E-03 | 8.64E-03 | 8.64E-03 | 9.39E-03 | 9.20E-03 | 9.12E-03 | 9.47E-03 |
| Dilated cardiomyopathy (DCM) | 0.00E+00 | 0.00E+00 | 0.00E+00 | 1.46E-07 | 4.17E-08 | 3.29E-08 | 0.00E+00 | 9.96E-08 | 0.00E+00 | 0.00E+00 | 0.00E+00 | 0.00E+00 | 0.00E+00 | 0.00E+00 | 0.00E+00 | 3.60E-08 | 0.00E+00 | 0.00E+00 | 4.55E-08 | 0.00E+00 | 0.00E+00 | 5.15E-07 | 1.37E-06 | 1.83E-07 |
| Dioxin degradation | 6.46E-04 | 5.02E-04 | 5.62E-04 | 6.05E-04 | 6.10E-04 | 6.10E-04 | 5.89E-04 | 6.22E-04 | 5.85E-04 | 6.73E-04 | 7.51E-04 | 5.61E-04 | 7.45E-04 | 7.35E-04 | 8.02E-04 | 7.91E-04 | 7.62E-04 | 8.81E-04 | 4.16E-04 | 6.24E-04 | 5.82E-04 | 4.33E-04 | 3.93E-04 | 4.84E-04 |
| Drug metabolism - cytochrome P450 | 2.38E-03 | 1.69E-03 | 1.51E-03 | 1.33E-03 | 1.93E-03 | 1.29E-03 | 1.49E-03 | 1.86E-03 | 1.29E-03 | 1.59E-03 | 1.50E-03 | 1.15E-03 | 1.51E-03 | 1.56E-03 | 1.38E-03 | 1.33E-03 | 1.40E-03 | 1.31E-03 | 1.75E-03 | 1.48E-03 | 1.26E-03 | 1.32E-03 | 1.52E-03 | 1.28E-03 |
| Drug metabolism - other enzymes | 2.06E-03 | 2.17E-03 | 2.12E-03 | 2.20E-03 | 1.88E-03 | 2.19E-03 | 2.23E-03 | 2.15E-03 | 2.28E-03 | 2.30E-03 | 2.30E-03 | 2.47E-03 | 1.94E-03 | 1.90E-03 | 1.99E-03 | 2.01E-03 | 2.00E-03 | 1.99E-03 | 2.29E-03 | 2.22E-03 | 2.09E-03 | 2.65E-03 | 2.08E-03 | 2.21E-03 |
| ECM-receptor interaction | 1.63E-08 | 0.00E+00 | 0.00E+00 | 2.93E-07 | 9.60E-07 | 1.88E-06 | 1.81E-06 | 1.84E-06 | 6.97E-07 | 0.00E+00 | 0.00E+00 | 0.00E+00 | 0.00E+00 | 6.00E-08 | 5.25E-08 | 0.00E+00 | 0.00E+00 | 0.00E+00 | 4.55E-08 | 0.00E+00 | 1.66E-07 | 0.00E+00 | 5.42E-07 | 6.09E-07 |
| Electron transfer carriers | 5.89E-04 | 6.45E-04 | 6.42E-04 | 9.29E-04 | 5.43E-04 | 9.07E-04 | 7.59E-04 | 7.91E-04 | 7.53E-04 | 9.77E-04 | 9.74E-04 | 1.22E-03 | 1.21E-03 | 1.23E-03 | 1.34E-03 | 1.39E-03 | 1.27E-03 | 1.47E-03 | 4.13E-04 | 7.18E-04 | 5.58E-04 | 6.48E-04 | 4.52E-04 | 4.72E-04 |
| Endocrine and other factor-regulated calcium reabsorption | 1.04E-06 | 7.00E-08 | 5.52E-08 | 1.46E-07 | 2.09E-07 | 9.87E-08 | 4.76E-08 | 1.99E-07 | 1.10E-07 | 0.00E+00 | 2.85E-08 | 0.00E+00 | 0.00E+00 | 0.00E+00 | 5.25E-08 | 0.00E+00 | 0.00E+00 | 0.00E+00 | 2.27E-07 | 5.87E-07 | 1.83E-06 | 1.34E-06 | 1.34E-06 | 1.52E-06 |
| Endocytosis | 2.70E-05 | 5.55E-06 | 1.49E-06 | 5.41E-06 | 5.92E-06 | 3.39E-06 | 1.01E-05 | 1.75E-05 | 4.07E-06 | 2.78E-06 | 1.71E-06 | 1.54E-06 | 5.04E-07 | 6.60E-07 | 6.83E-07 | 5.40E-07 | 9.66E-07 | 7.03E-07 | 3.45E-06 | 3.82E-06 | 2.99E-06 | 1.23E-05 | 6.07E-06 | 7.43E-06 |
| Energy metabolism | 8.61E-03 | 9.35E-03 | 8.55E-03 | 9.30E-03 | 7.53E-03 | 9.39E-03 | 9.43E-03 | 9.00E-03 | 9.39E-03 | 9.09E-03 | 9.16E-03 | 9.80E-03 | 9.60E-03 | 9.62E-03 | 9.74E-03 | 9.73E-03 | 9.64E-03 | 9.91E-03 | 8.53E-03 | 8.72E-03 | 8.56E-03 | 7.86E-03 | 8.88E-03 | 9.13E-03 |
| Epithelial cell signaling in Helicobacter pylori infection | 4.70E-04 | 6.15E-04 | 5.62E-04 | 6.33E-04 | 6.41E-04 | 6.37E-04 | 6.00E-04 | 5.52E-04 | 6.41E-04 | 5.17E-04 | 5.54E-04 | 8.02E-04 | 6.01E-04 | 5.97E-04 | 6.17E-04 | 6.15E-04 | 6.15E-04 | 6.30E-04 | 3.57E-04 | 4.94E-04 | 6.18E-04 | 4.15E-04 | 5.00E-04 | 5.53E-04 |
| ErbB signaling pathway | 0.00E+00 | 0.00E+00 | 0.00E+00 | 0.00E+00 | 0.00E+00 | 0.00E+00 | 0.00E+00 | 0.00E+00 | 0.00E+00 | 0.00E+00 | 0.00E+00 | 0.00E+00 | 0.00E+00 | 0.00E+00 | 0.00E+00 | 0.00E+00 | 0.00E+00 | 0.00E+00 | 0.00E+00 | 0.00E+00 | 0.00E+00 | 0.00E+00 | 0.00E+00 | 0.00E+00 |
| Ether lipid metabolism | 1.80E-04 | 8.29E-05 | 4.52E-05 | 2.53E-05 | 1.69E-05 | 1.78E-05 | 2.34E-05 | 3.72E-05 | 1.73E-05 | 3.70E-05 | 3.65E-05 | 3.68E-05 | 4.11E-05 | 4.71E-05 | 2.76E-05 | 2.42E-05 | 2.95E-05 | 1.85E-05 | 6.24E-05 | 6.75E-05 | 4.32E-05 | 4.14E-05 | 5.27E-05 | 6.49E-05 |
| Ethylbenzene degradation | 7.68E-04 | 8.59E-04 | 6.75E-04 | 6.64E-04 | 5.32E-04 | 6.72E-04 | 7.21E-04 | 6.49E-04 | 6.54E-04 | 7.16E-04 | 7.10E-04 | 9.13E-04 | 5.40E-04 | 5.17E-04 | 5.51E-04 | 5.43E-04 | 5.60E-04 | 5.48E-04 | 9.25E-04 | 7.01E-04 | 7.28E-04 | 7.74E-04 | 6.60E-04 | 9.48E-04 |
| Fat digestion and absorption | 0.00E+00 | 2.33E-08 | 1.66E-07 | 0.00E+00 | 0.00E+00 | 6.58E-08 | 4.76E-08 | 4.98E-08 | 7.34E-08 | 0.00E+00 | 0.00E+00 | 0.00E+00 | 0.00E+00 | 0.00E+00 | 0.00E+00 | 0.00E+00 | 0.00E+00 | 0.00E+00 | 1.36E-07 | 2.93E-07 | 0.00E+00 | 2.06E-07 | 3.61E-07 | 6.09E-08 |
| Fatty acid biosynthesis | 4.69E-03 | 4.42E-03 | 4.54E-03 | 4.25E-03 | 4.50E-03 | 4.18E-03 | 4.43E-03 | 4.60E-03 | 4.34E-03 | 3.92E-03 | 4.09E-03 | 4.08E-03 | 4.10E-03 | 4.15E-03 | 3.91E-03 | 3.87E-03 | 3.99E-03 | 3.72E-03 | 5.05E-03 | 4.80E-03 | 5.39E-03 | 4.52E-03 | 5.25E-03 | 5.68E-03 |
| Fatty acid elongation in mitochondria | 6.53E-08 | 4.43E-07 | 1.85E-06 | 1.46E-07 | 2.50E-07 | 2.30E-07 | 7.62E-07 | 8.97E-07 | 1.10E-07 | 6.71E-08 | 2.85E-08 | 0.00E+00 | 6.30E-08 | 0.00E+00 | 0.00E+00 | 3.60E-08 | 0.00E+00 | 5.86E-08 | 9.09E-08 | 0.00E+00 | 0.00E+00 | 6.19E-07 | 1.52E-06 | 3.05E-07 |
| Fatty acid metabolism | 8.00E-03 | 6.86E-03 | 6.27E-03 | 5.79E-03 | 5.15E-03 | 5.74E-03 | 6.64E-03 | 6.84E-03 | 5.67E-03 | 5.87E-03 | 5.64E-03 | 6.32E-03 | 5.63E-03 | 5.68E-03 | 5.37E-03 | 5.25E-03 | 5.46E-03 | 5.13E-03 | 7.74E-03 | 6.77E-03 | 5.98E-03 | 6.47E-03 | 6.65E-03 | 5.93E-03 |
| Fc epsilon RI signaling pathway | 0.00E+00 | 2.33E-08 | 2.76E-08 | 0.00E+00 | 0.00E+00 | 6.58E-08 | 0.00E+00 | 0.00E+00 | 7.34E-08 | 0.00E+00 | 0.00E+00 | 0.00E+00 | 0.00E+00 | 0.00E+00 | 0.00E+00 | 0.00E+00 | 0.00E+00 | 0.00E+00 | 9.09E-08 | 2.93E-07 | 0.00E+00 | 0.00E+00 | 1.45E-07 | 0.00E+00 |
| Fc gamma R-mediated phagocytosis | 2.70E-05 | 5.55E-06 | 1.63E-06 | 6.00E-06 | 9.43E-06 | 3.42E-06 | 1.01E-05 | 2.45E-05 | 4.15E-06 | 2.78E-06 | 1.71E-06 | 1.54E-06 | 5.04E-07 | 6.60E-07 | 7.36E-07 | 5.40E-07 | 9.66E-07 | 7.03E-07 | 3.55E-06 | 4.40E-06 | 2.99E-06 | 1.27E-05 | 6.39E-06 | 7.49E-06 |
| Flagellar assembly | 8.41E-03 | 9.91E-03 | 8.25E-03 | 1.04E-02 | 5.32E-03 | 1.05E-02 | 9.35E-03 | 8.04E-03 | 9.04E-03 | 7.08E-03 | 7.28E-03 | 9.33E-03 | 8.89E-03 | 8.67E-03 | 9.05E-03 | 9.08E-03 | 9.10E-03 | 9.12E-03 | 7.09E-03 | 7.29E-03 | 6.18E-03 | 6.13E-03 | 8.39E-03 | 6.58E-03 |
| Flavone and flavonol biosynthesis | 2.83E-05 | 1.05E-05 | 1.62E-05 | 4.04E-05 | 2.85E-05 | 4.32E-05 | 2.93E-05 | 3.61E-05 | 6.35E-05 | 7.11E-05 | 8.84E-05 | 2.48E-05 | 6.02E-05 | 6.02E-05 | 7.75E-05 | 7.65E-05 | 6.61E-05 | 9.80E-05 | 2.18E-05 | 3.58E-05 | 2.69E-05 | 2.53E-05 | 1.52E-05 | 5.39E-05 |
| Flavonoid biosynthesis | 9.49E-05 | 8.33E-05 | 2.40E-04 | 6.54E-05 | 4.53E-04 | 7.07E-05 | 1.10E-04 | 1.41E-04 | 1.27E-04 | 2.86E-05 | 3.08E-05 | 6.11E-05 | 5.04E-05 | 5.27E-05 | 3.31E-05 | 2.85E-05 | 3.84E-05 | 2.47E-05 | 1.34E-04 | 1.41E-04 | 1.02E-04 | 1.84E-04 | 1.85E-04 | 8.81E-05 |
| Fluorobenzoate degradation | 5.36E-04 | 3.52E-04 | 3.64E-04 | 3.05E-04 | 4.41E-04 | 2.74E-04 | 3.12E-04 | 3.97E-04 | 3.08E-04 | 3.52E-04 | 3.41E-04 | 1.64E-04 | 5.40E-04 | 5.87E-04 | 4.57E-04 | 4.32E-04 | 4.48E-04 | 4.35E-04 | 3.54E-04 | 4.41E-04 | 3.51E-04 | 3.50E-04 | 2.98E-04 | 3.04E-04 |
| Focal adhesion | 1.63E-08 | 0.00E+00 | 0.00E+00 | 4.39E-07 | 1.00E-06 | 1.91E-06 | 1.81E-06 | 1.94E-06 | 6.97E-07 | 0.00E+00 | 0.00E+00 | 0.00E+00 | 0.00E+00 | 6.00E-08 | 5.25E-08 | 3.60E-08 | 0.00E+00 | 0.00E+00 | 9.09E-08 | 0.00E+00 | 1.66E-07 | 5.15E-07 | 1.91E-06 | 7.92E-07 |
| Folate biosynthesis | 4.12E-03 | 4.19E-03 | 4.30E-03 | 4.11E-03 | 4.42E-03 | 4.15E-03 | 4.15E-03 | 4.14E-03 | 4.22E-03 | 4.50E-03 | 4.46E-03 | 3.77E-03 | 4.17E-03 | 4.18E-03 | 4.12E-03 | 4.12E-03 | 4.13E-03 | 4.11E-03 | 4.73E-03 | 4.39E-03 | 4.53E-03 | 4.46E-03 | 4.84E-03 | 4.66E-03 |
| Fructose and mannose metabolism | 5.09E-03 | 5.16E-03 | 5.93E-03 | 6.18E-03 | 6.72E-03 | 6.20E-03 | 5.99E-03 | 6.11E-03 | 6.53E-03 | 6.77E-03 | 7.15E-03 | 5.42E-03 | 6.75E-03 | 6.78E-03 | 7.05E-03 | 7.14E-03 | 6.95E-03 | 7.16E-03 | 5.70E-03 | 6.31E-03 | 6.45E-03 | 6.84E-03 | 5.47E-03 | 6.42E-03 |
| Function unknown | 1.90E-02 | 2.26E-02 | 1.99E-02 | 2.33E-02 | 1.78E-02 | 2.35E-02 | 2.17E-02 | 2.02E-02 | 2.17E-02 | 2.27E-02 | 2.25E-02 | 2.21E-02 | 2.38E-02 | 2.37E-02 | 2.39E-02 | 2.40E-02 | 2.38E-02 | 2.40E-02 | 1.95E-02 | 1.97E-02 | 1.88E-02 | 1.85E-02 | 1.92E-02 | 1.93E-02 |
| G protein-coupled receptors | 2.30E-06 | 2.33E-08 | 2.76E-08 | 0.00E+00 | 3.75E-07 | 3.29E-08 | 4.76E-08 | 9.96E-08 | 3.67E-08 | 0.00E+00 | 1.42E-07 | 0.00E+00 | 6.30E-08 | 6.00E-08 | 0.00E+00 | 0.00E+00 | 0.00E+00 | 0.00E+00 | 8.64E-07 | 8.80E-07 | 9.97E-07 | 6.19E-07 | 3.61E-07 | 2.07E-06 |
| GTP-binding proteins | 8.32E-07 | 2.33E-08 | 5.52E-08 | 1.46E-07 | 0.00E+00 | 0.00E+00 | 0.00E+00 | 4.98E-08 | 0.00E+00 | 0.00E+00 | 0.00E+00 | 0.00E+00 | 0.00E+00 | 0.00E+00 | 0.00E+00 | 0.00E+00 | 0.00E+00 | 0.00E+00 | 0.00E+00 | 0.00E+00 | 0.00E+00 | 0.00E+00 | 0.00E+00 | 0.00E+00 |
| Galactose metabolism | 3.45E-03 | 3.51E-03 | 3.63E-03 | 4.04E-03 | 3.84E-03 | 4.12E-03 | 3.90E-03 | 3.77E-03 | 4.35E-03 | 4.72E-03 | 5.13E-03 | 4.56E-03 | 4.66E-03 | 4.69E-03 | 5.08E-03 | 5.12E-03 | 4.86E-03 | 5.55E-03 | 3.37E-03 | 4.29E-03 | 3.88E-03 | 4.64E-03 | 3.41E-03 | 3.72E-03 |
| Gastric acid secretion | 1.04E-06 | 7.00E-08 | 5.52E-08 | 1.46E-07 | 2.09E-07 | 9.87E-08 | 4.76E-08 | 1.99E-07 | 1.10E-07 | 0.00E+00 | 2.85E-08 | 0.00E+00 | 0.00E+00 | 0.00E+00 | 5.25E-08 | 0.00E+00 | 0.00E+00 | 0.00E+00 | 2.27E-07 | 5.87E-07 | 1.83E-06 | 1.34E-06 | 1.34E-06 | 1.52E-06 |
| General function prediction only | 3.32E-02 | 3.28E-02 | 3.31E-02 | 3.29E-02 | 3.55E-02 | 3.30E-02 | 3.29E-02 | 3.41E-02 | 3.37E-02 | 3.52E-02 | 3.54E-02 | 3.13E-02 | 3.52E-02 | 3.55E-02 | 3.50E-02 | 3.50E-02 | 3.49E-02 | 3.51E-02 | 3.49E-02 | 3.48E-02 | 3.58E-02 | 3.29E-02 | 3.45E-02 | 3.63E-02 |
| Geraniol degradation | 4.10E-03 | 3.87E-03 | 3.17E-03 | 3.06E-03 | 2.03E-03 | 3.02E-03 | 3.47E-03 | 3.39E-03 | 2.81E-03 | 3.15E-03 | 2.94E-03 | 3.66E-03 | 3.01E-03 | 3.04E-03 | 2.76E-03 | 2.67E-03 | 2.82E-03 | 2.53E-03 | 4.28E-03 | 3.46E-03 | 2.67E-03 | 3.08E-03 | 3.22E-03 | 2.79E-03 |
| Germination | 3.36E-05 | 1.41E-05 | 5.26E-05 | 9.66E-06 | 2.51E-05 | 6.75E-06 | 9.29E-06 | 8.17E-06 | 5.69E-06 | 6.54E-06 | 2.73E-05 | 1.97E-05 | 4.92E-06 | 2.70E-06 | 2.78E-06 | 3.49E-06 | 6.25E-06 | 1.41E-06 | 1.04E-05 | 2.11E-05 | 2.62E-05 | 2.13E-05 | 1.53E-05 | 1.11E-05 |
| Glioma | 0.00E+00 | 0.00E+00 | 0.00E+00 | 0.00E+00 | 0.00E+00 | 0.00E+00 | 0.00E+00 | 0.00E+00 | 0.00E+00 | 0.00E+00 | 0.00E+00 | 0.00E+00 | 0.00E+00 | 0.00E+00 | 0.00E+00 | 0.00E+00 | 0.00E+00 | 0.00E+00 | 0.00E+00 | 0.00E+00 | 0.00E+00 | 0.00E+00 | 0.00E+00 | 0.00E+00 |

| | | | | | | | | | | | | | | | | | | | | | | | | |
|---|---|---|---|---|---|---|---|---|---|---|---|---|---|---|---|---|---|---|---|---|---|---|---|---|
| Glutamatergic synapse | 9.34E-04 | 7.03E-04 | 7.92E-04 | 6.83E-04 | 8.53E-04 | 6.45E-04 | 7.48E-04 | 8.80E-04 | 6.68E-04 | 7.09E-04 | 6.74E-04 | 7.22E-04 | 7.63E-04 | 7.98E-04 | 6.93E-04 | 6.74E-04 | 6.99E-04 | 6.67E-04 | 7.60E-04 | 8.22E-04 | 7.80E-04 | 9.31E-04 | 7.47E-04 | 8.11E-04 |
| Glutathione metabolism | 5.05E-03 | 4.55E-03 | 4.17E-03 | 4.21E-03 | 4.53E-03 | 4.15E-03 | 4.28E-03 | 4.58E-03 | 4.08E-03 | 4.51E-03 | 4.36E-03 | 4.31E-03 | 4.46E-03 | 4.53E-03 | 4.34E-03 | 4.34E-03 | 4.36E-03 | 4.25E-03 | 4.26E-03 | 4.05E-03 | 3.31E-03 | 4.11E-03 | 4.33E-03 | 3.48E-03 |
| Glycan bindng proteins | 0.00E+00 | 0.00E+00 | 0.00E+00 | 0.00E+00 | 0.00E+00 | 0.00E+00 | 0.00E+00 | 0.00E+00 | 0.00E+00 | 0.00E+00 | 0.00E+00 | 0.00E+00 | 0.00E+00 | 0.00E+00 | 0.00E+00 | 0.00E+00 | 0.00E+00 | 0.00E+00 | 0.00E+00 | 0.00E+00 | 0.00E+00 | 0.00E+00 | 0.00E+00 | 0.00E+00 |
| Glycan biosynthesis and metabolism | 8.63E-04 | 1.10E-03 | 9.72E-04 | 1.20E-03 | 8.73E-04 | 1.24E-03 | 1.07E-03 | 1.00E-03 | 1.10E-03 | 1.21E-03 | 1.20E-03 | 1.21E-03 | 1.39E-03 | 1.38E-03 | 1.47E-03 | 1.49E-03 | 1.42E-03 | 1.54E-03 | 8.60E-04 | 1.02E-03 | 9.92E-04 | 8.08E-04 | 1.05E-03 | 9.77E-04 |
| Glycerolipid metabolism | 3.25E-03 | 3.05E-03 | 3.31E-03 | 3.10E-03 | 3.28E-03 | 3.11E-03 | 3.10E-03 | 3.17E-03 | 3.08E-03 | 3.50E-03 | 3.43E-03 | 2.95E-03 | 3.29E-03 | 3.33E-03 | 3.30E-03 | 3.28E-03 | 3.26E-03 | 3.34E-03 | 3.21E-03 | 3.31E-03 | 3.27E-03 | 3.34E-03 | 3.10E-03 | 3.06E-03 |
| Glycerophospholipid metabolism | 5.05E-03 | 5.11E-03 | 4.81E-03 | 5.17E-03 | 4.51E-03 | 5.16E-03 | 5.02E-03 | 5.01E-03 | 4.97E-03 | 5.77E-03 | 5.69E-03 | 5.45E-03 | 5.68E-03 | 5.72E-03 | 5.79E-03 | 5.82E-03 | 5.71E-03 | 5.96E-03 | 5.24E-03 | 5.43E-03 | 5.77E-03 | 5.17E-03 | 5.41E-03 | 5.69E-03 |
| Glycine, serine and threonine metabolism | 9.54E-03 | 8.92E-03 | 8.65E-03 | 9.06E-03 | 8.14E-03 | 8.98E-03 | 9.58E-03 | 9.93E-03 | 9.10E-03 | 8.40E-03 | 8.21E-03 | 8.73E-03 | 8.54E-03 | 8.53E-03 | 8.34E-03 | 8.30E-03 | 8.43E-03 | 8.14E-03 | 9.30E-03 | 9.05E-03 | 8.89E-03 | 9.72E-03 | 9.44E-03 | 9.28E-03 |
| Glycolysis / Gluconeogenesis | 9.25E-03 | 9.01E-03 | 9.71E-03 | 9.07E-03 | 1.03E-02 | 9.12E-03 | 9.43E-03 | 9.58E-03 | 9.41E-03 | 9.65E-03 | 9.61E-03 | 9.22E-03 | 8.88E-03 | 8.82E-03 | 9.05E-03 | 9.08E-03 | 9.05E-03 | 9.12E-03 | 9.83E-03 | 9.70E-03 | 9.50E-03 | 1.08E-02 | 9.77E-03 | 9.66E-03 |
| Glycosaminoglycan biosynthesis - chondroitin sulfate | 1.39E-06 | 4.66E-06 | 1.71E-06 | 8.78E-07 | 9.18E-07 | 3.95E-07 | 1.43E-07 | 1.49E-07 | 5.14E-07 | 1.01E-07 | 2.85E-07 | 1.72E-07 | 6.30E-08 | 1.20E-07 | 3.68E-07 | 1.08E-07 | 1.84E-07 | 0.00E+00 | 4.55E-08 | 2.93E-07 | 6.64E-07 | 0.00E+00 | 1.81E-07 | 2.44E-07 |
| Glycosaminoglycan degradation | 1.11E-04 | 3.90E-04 | 2.95E-04 | 4.03E-04 | 2.38E-04 | 4.21E-04 | 3.92E-04 | 2.37E-04 | 4.38E-04 | 2.23E-04 | 2.46E-04 | 5.33E-04 | 2.11E-04 | 1.91E-04 | 2.15E-04 | 2.22E-04 | 2.28E-04 | 1.99E-04 | 2.10E-04 | 2.12E-04 | 1.60E-04 | 2.79E-04 | 1.87E-04 | 2.89E-04 |
| Glycosphingolipid biosynthesis - ganglio series | 6.92E-05 | 3.37E-04 | 2.68E-04 | 3.44E-04 | 1.84E-04 | 3.57E-04 | 3.28E-04 | 1.86E-04 | 3.33E-04 | 1.47E-04 | 1.47E-04 | 4.09E-04 | 1.49E-04 | 1.28E-04 | 1.36E-04 | 1.43E-04 | 1.59E-04 | 9.85E-05 | 1.80E-04 | 1.58E-04 | 1.23E-04 | 2.35E-04 | 1.62E-04 | 1.66E-04 |
| Glycosphingolipid biosynthesis - globo series | 1.51E-04 | 5.62E-04 | 5.10E-04 | 5.81E-04 | 3.53E-04 | 6.32E-04 | 6.10E-04 | 3.79E-04 | 6.27E-04 | 2.52E-04 | 3.05E-04 | 6.91E-04 | 2.66E-04 | 2.27E-04 | 2.64E-04 | 2.60E-04 | 2.83E-04 | 2.34E-04 | 3.78E-04 | 3.67E-04 | 3.71E-04 | 4.90E-04 | 3.52E-04 | 3.58E-04 |
| Glycosphingolipid biosynthesis - lacto and neolacto series | 2.25E-05 | 1.45E-06 | 1.10E-07 | 1.46E-07 | 1.25E-07 | 3.29E-08 | 0.00E+00 | 0.00E+00 | 1.47E-07 | 4.36E-07 | 1.42E-07 | 0.00E+00 | 0.00E+00 | 0.00E+00 | 0.00E+00 | 3.60E-08 | 0.00E+00 | 0.00E+00 | 4.55E-08 | 2.93E-07 | 0.00E+00 | 0.00E+00 | 0.00E+00 | 0.00E+00 |
| Glycosylphosphatidylinositol(GPI)-anchor biosynthesis | 3.59E-07 | 7.70E-07 | 1.24E-06 | 1.46E-06 | 8.76E-07 | 1.12E-06 | 1.05E-06 | 2.04E-06 | 7.34E-07 | 0.00E+00 | 0.00E+00 | 1.14E-07 | 5.04E-07 | 3.00E-07 | 3.68E-07 | 2.16E-07 | 4.60E-07 | 1.76E-07 | 1.68E-06 | 2.05E-06 | 1.33E-06 | 1.96E-06 | 3.41E-05 | 2.25E-06 |
| Glycosyltransferases | 3.50E-03 | 3.14E-03 | 3.59E-03 | 3.66E-03 | 4.61E-03 | 3.62E-03 | 3.47E-03 | 3.84E-03 | 3.75E-03 | 4.49E-03 | 4.50E-03 | 3.46E-03 | 4.81E-03 | 4.95E-03 | 4.94E-03 | 5.01E-03 | 4.79E-03 | 5.16E-03 | 3.31E-03 | 4.01E-03 | 4.04E-03 | 3.36E-03 | 3.44E-03 | 3.97E-03 |
| Glyoxylate and dicarboxylate metabolism | 8.64E-03 | 6.64E-03 | 6.21E-03 | 6.72E-03 | 5.86E-03 | 6.57E-03 | 6.78E-03 | 7.36E-03 | 6.38E-03 | 6.92E-03 | 6.69E-03 | 6.11E-03 | 7.44E-03 | 7.60E-03 | 7.45E-03 | 7.44E-03 | 7.35E-03 | 7.63E-03 | 6.65E-03 | 6.89E-03 | 6.06E-03 | 6.51E-03 | 7.01E-03 | 6.32E-03 |
| GnRH signaling pathway | 2.70E-05 | 5.57E-06 | 1.52E-06 | 5.41E-06 | 5.92E-06 | 3.46E-06 | 1.01E-05 | 1.75E-05 | 4.15E-06 | 2.78E-06 | 1.71E-06 | 1.54E-06 | 5.04E-07 | 6.60E-07 | 6.83E-07 | 5.40E-07 | 9.66E-07 | 7.03E-07 | 3.55E-06 | 4.11E-06 | 2.99E-06 | 1.23E-05 | 6.21E-06 | 7.43E-06 |
| Hedgehog signaling pathway | 0.00E+00 | 0.00E+00 | 0.00E+00 | 0.00E+00 | 0.00E+00 | 0.00E+00 | 0.00E+00 | 0.00E+00 | 0.00E+00 | 0.00E+00 | 0.00E+00 | 0.00E+00 | 0.00E+00 | 0.00E+00 | 0.00E+00 | 0.00E+00 | 0.00E+00 | 0.00E+00 | 0.00E+00 | 0.00E+00 | 0.00E+00 | 0.00E+00 | 0.00E+00 | 0.00E+00 |
| Hematopoietic cell lineage | 1.31E-06 | 2.33E-08 | 1.10E-07 | 0.00E+00 | 0.00E+00 | 0.00E+00 | 4.76E-08 | 0.00E+00 | 0.00E+00 | 3.35E-08 | 1.42E-07 | 0.00E+00 | 0.00E+00 | 0.00E+00 | 0.00E+00 | 0.00E+00 | 0.00E+00 | 0.00E+00 | 2.27E-07 | 0.00E+00 | 0.00E+00 | 1.03E-07 | 3.61E-08 | 1.22E-07 |
| Hepatitis C | 0.00E+00 | 0.00E+00 | 0.00E+00 | 0.00E+00 | 0.00E+00 | 0.00E+00 | 0.00E+00 | 0.00E+00 | 0.00E+00 | 0.00E+00 | 0.00E+00 | 0.00E+00 | 0.00E+00 | 0.00E+00 | 0.00E+00 | 0.00E+00 | 0.00E+00 | 0.00E+00 | 0.00E+00 | 0.00E+00 | 0.00E+00 | 0.00E+00 | 0.00E+00 | 0.00E+00 |
| Histidine metabolism | 5.16E-03 | 5.07E-03 | 5.03E-03 | 4.57E-03 | 5.04E-03 | 4.55E-03 | 4.90E-03 | 5.00E-03 | 4.91E-03 | 4.43E-03 | 4.25E-03 | 4.53E-03 | 4.12E-03 | 4.15E-03 | 3.82E-03 | 3.76E-03 | 3.98E-03 | 3.50E-03 | 5.69E-03 | 5.13E-03 | 5.54E-03 | 5.74E-03 | 5.38E-03 | 5.81E-03 |
| Homologous recombination | 6.03E-03 | 6.70E-03 | 7.09E-03 | 6.63E-03 | 7.18E-03 | 6.72E-03 | 6.85E-03 | 6.68E-03 | 7.02E-03 | 6.97E-03 | 7.05E-03 | 6.68E-03 | 6.17E-03 | 6.12E-03 | 6.20E-03 | 6.23E-03 | 6.26E-03 | 6.14E-03 | 7.62E-03 | 7.13E-03 | 8.14E-03 | 7.66E-03 | 7.84E-03 | 8.15E-03 |
| Huntington's disease | 1.21E-03 | 1.13E-03 | 9.20E-04 | 1.01E-03 | 8.76E-04 | 1.02E-03 | 1.10E-03 | 1.04E-03 | 1.02E-03 | 8.09E-04 | 7.88E-04 | 1.10E-03 | 8.35E-04 | 8.23E-04 | 7.72E-04 | 7.74E-04 | 7.99E-04 | 6.93E-04 | 9.21E-04 | 7.89E-04 | 6.93E-04 | 8.36E-04 | 1.07E-03 | 8.63E-04 |
| Hypertrophic cardiomyopathy (HCM) | 1.22E-05 | 1.07E-05 | 4.77E-06 | 2.78E-06 | 2.09E-06 | 1.84E-06 | 2.19E-06 | 2.29E-06 | 1.61E-06 | 1.39E-05 | 1.07E-05 | 4.63E-06 | 5.67E-07 | 3.60E-07 | 7.88E-07 | 4.68E-07 | 1.20E-06 | 2.34E-07 | 3.68E-06 | 5.87E-06 | 1.66E-06 | 5.57E-06 | 1.03E-05 | 2.74E-06 |
| Indole alkaloid biosynthesis | 3.27E-05 | 4.73E-06 | 6.92E-06 | 7.90E-06 | 1.09E-05 | 4.97E-06 | 1.84E-05 | 3.95E-05 | 8.33E-06 | 6.71E-07 | 7.12E-07 | 1.89E-06 | 3.78E-07 | 9.60E-07 | 7.88E-07 | 7.56E-07 | 1.24E-06 | 7.03E-07 | 6.54E-06 | 7.34E-06 | 2.77E-05 | 1.88E-05 | 1.47E-05 | 2.89E-05 |
| Influenza A | 2.58E-04 | 4.32E-05 | 3.34E-05 | 4.08E-05 | 5.09E-05 | 2.99E-05 | 9.34E-05 | 1.59E-04 | 4.61E-05 | 1.30E-05 | 1.18E-05 | 1.24E-05 | 3.03E-06 | 4.02E-06 | 4.20E-06 | 3.96E-06 | 6.02E-06 | 4.10E-06 | 3.49E-05 | 4.70E-05 | 2.06E-05 | 7.45E-05 | 6.95E-05 | 4.54E-05 |
| Inorganic ion transport and metabolism | 4.11E-03 | 3.96E-03 | 3.40E-03 | 4.33E-03 | 2.83E-03 | 4.32E-03 | 3.89E-03 | 3.89E-03 | 3.85E-03 | 4.69E-03 | 4.57E-03 | 3.81E-03 | 5.00E-03 | 5.05E-03 | 5.16E-03 | 5.21E-03 | 5.05E-03 | 5.34E-03 | 3.62E-03 | 3.83E-03 | 3.05E-03 | 3.69E-03 | 3.53E-03 | 3.14E-03 |
| Inositol phosphate metabolism | 2.18E-03 | 1.74E-03 | 1.61E-03 | 1.57E-03 | 1.38E-03 | 1.61E-03 | 1.68E-03 | 1.67E-03 | 1.63E-03 | 1.72E-03 | 1.51E-03 | 1.33E-03 | 1.62E-03 | 1.63E-03 | 1.56E-03 | 1.48E-03 | 1.52E-03 | 1.63E-03 | 1.65E-03 | 1.69E-03 | 1.52E-03 | 1.63E-03 | 1.46E-03 | 1.45E-03 |
| Insulin signaling pathway | 5.37E-04 | 5.66E-04 | 7.74E-04 | 5.63E-04 | 1.17E-03 | 5.76E-04 | 5.73E-04 | 6.40E-04 | 6.41E-04 | 6.17E-04 | 6.43E-04 | 5.13E-04 | 5.56E-04 | 5.54E-04 | 5.78E-04 | 5.78E-04 | 5.77E-04 | 5.95E-04 | 6.14E-04 | 6.59E-04 | 8.31E-04 | 7.09E-04 | 6.99E-04 | 7.45E-04 |
| Ion channels | 2.62E-04 | 3.18E-04 | 2.88E-04 | 3.61E-04 | 3.07E-04 | 3.62E-04 | 3.11E-04 | 2.63E-04 | 3.28E-04 | 4.01E-04 | 4.28E-04 | 3.91E-04 | 3.63E-04 | 3.55E-04 | 3.68E-04 | 3.68E-04 | 3.62E-04 | 3.72E-04 | 3.18E-04 | 2.75E-04 | 2.38E-04 | 2.88E-04 | 1.57E-04 | 2.16E-04 |
| Isoflavonoid biosynthesis | 6.30E-06 | 1.10E-06 | 1.66E-06 | 5.41E-06 | 7.72E-06 | 4.61E-06 | 4.72E-06 | 1.03E-05 | 4.66E-06 | 2.35E-07 | 3.99E-07 | 6.87E-07 | 1.89E-06 | 2.40E-06 | 3.05E-06 | 1.22E-06 | 3.49E-06 | 2.75E-06 | 2.31E-05 | 3.08E-05 | 1.15E-04 | 2.83E-05 | 2.89E-05 | 9.74E-05 |
| Isoquinoline alkaloid biosynthesis | 6.22E-04 | 5.20E-04 | 5.07E-04 | 5.51E-04 | 4.85E-04 | 5.34E-04 | 5.50E-04 | 6.10E-04 | 5.48E-04 | 5.20E-04 | 5.32E-04 | 5.19E-04 | 6.59E-04 | 6.77E-04 | 6.22E-04 | 6.09E-04 | 6.16E-04 | 6.17E-04 | 5.60E-04 | 5.78E-04 | 5.49E-04 | 5.61E-04 | 5.22E-04 | 6.02E-04 |
| Leishmaniasis | 1.16E-06 | 7.00E-08 | 1.10E-07 | 4.39E-07 | 4.59E-07 | 9.87E-08 | 4.76E-07 | 1.15E-06 | 1.47E-07 | 3.35E-08 | 8.54E-08 | 0.00E+00 | 0.00E+00 | 6.00E-08 | 0.00E+00 | 3.60E-08 | 0.00E+00 | 0.00E+00 | 0.00E+00 | 0.00E+00 | 0.00E+00 | 0.00E+00 | 3.61E-07 | 0.00E+00 |
| Leukocyte transendothelial migration | 0.00E+00 | 0.00E+00 | 0.00E+00 | 1.46E-07 | 4.17E-08 | 3.29E-08 | 0.00E+00 | 9.96E-08 | 0.00E+00 | 0.00E+00 | 0.00E+00 | 0.00E+00 | 0.00E+00 | 0.00E+00 | 0.00E+00 | 3.60E-08 | 0.00E+00 | 0.00E+00 | 4.55E-08 | 0.00E+00 | 0.00E+00 | 5.15E-07 | 1.37E-06 | 1.83E-07 |
| Limonene and pinene degradation | 4.23E-03 | 3.62E-03 | 3.11E-03 | 2.77E-03 | 2.16E-03 | 2.71E-03 | 3.32E-03 | 3.46E-03 | 2.63E-03 | 2.91E-03 | 2.75E-03 | 3.32E-03 | 2.44E-03 | 2.44E-03 | 2.27E-03 | 2.20E-03 | 2.35E-03 | 2.07E-03 | 4.09E-03 | 3.28E-03 | 2.82E-03 | 3.07E-03 | 3.16E-03 | 3.24E-03 |
| Linoleic acid metabolism | 5.61E-04 | 5.86E-04 | 4.73E-04 | 4.37E-04 | 3.03E-04 | 4.43E-04 | 4.83E-04 | 4.04E-04 | 4.42E-04 | 4.56E-04 | 4.75E-04 | 4.71E-04 | 2.55E-04 | 2.38E-04 | 2.09E-04 | 1.91E-04 | 2.37E-04 | 1.39E-04 | 7.40E-04 | 4.67E-04 | 4.29E-04 | 4.71E-04 | 4.96E-04 | 5.98E-04 |
| Lipid biosynthesis proteins | 7.17E-03 | 6.88E-03 | 6.92E-03 | 6.33E-03 | 6.57E-03 | 6.29E-03 | 6.76E-03 | 6.88E-03 | 6.51E-03 | 6.09E-03 | 5.97E-03 | 6.17E-03 | 6.01E-03 | 6.05E-03 | 5.72E-03 | 5.63E-03 | 5.84E-03 | 5.45E-03 | 7.65E-03 | 7.14E-03 | 7.58E-03 | 6.85E-03 | 7.72E-03 | 7.78E-03 |
| Lipid metabolism | 1.40E-03 | 1.21E-03 | 1.21E-03 | 1.21E-03 | 1.36E-03 | 1.20E-03 | 1.14E-03 | 1.25E-03 | 1.15E-03 | 1.29E-03 | 1.32E-03 | 9.04E-04 | 1.58E-03 | 1.60E-03 | 1.50E-03 | 1.45E-03 | 1.49E-03 | 1.45E-03 | 1.18E-03 | 1.35E-03 | 1.39E-03 | 1.09E-03 | 1.14E-03 | 1.27E-03 |
| Lipoic acid metabolism | 6.29E-04 | 6.32E-04 | 7.47E-04 | 5.86E-04 | 9.30E-04 | 5.93E-04 | 6.33E-04 | 6.88E-04 | 6.58E-04 | 6.83E-04 | 6.52E-04 | 6.84E-04 | 6.18E-04 | 6.18E-04 | 6.30E-04 | 6.47E-04 | 6.26E-04 | 6.53E-04 | 6.54E-04 | 6.60E-04 | 5.44E-04 | 7.93E-04 | 6.77E-04 | 5.95E-04 |
| Lipopolysaccharide biosynthesis | 3.53E-03 | 4.07E-03 | 3.61E-03 | 4.43E-03 | 3.32E-03 | 4.46E-03 | 4.27E-03 | 4.11E-03 | 4.34E-03 | 4.65E-03 | 4.62E-03 | 4.19E-03 | 5.18E-03 | 5.23E-03 | 5.32E-03 | 5.42E-03 | 5.21E-03 | 5.46E-03 | 3.92E-03 | 4.09E-03 | 4.27E-03 | 3.30E-03 | 4.42E-03 | 4.52E-03 |
| Lipopolysaccharide biosynthesis proteins | 5.01E-03 | 5.37E-03 | 4.67E-03 | 6.30E-03 | 4.30E-03 | 6.23E-03 | 5.63E-03 | 5.63E-03 | 5.88E-03 | 7.12E-03 | 7.11E-03 | 5.81E-03 | 8.30E-03 | 8.49E-03 | 8.46E-03 | 8.59E-03 | 8.21E-03 | 8.73E-03 | 5.22E-03 | 6.00E-03 | 6.12E-03 | 4.72E-03 | 5.64E-03 | 6.22E-03 |
| Long-term depression | 0.00E+00 | 2.33E-08 | 2.76E-08 | 0.00E+00 | 0.00E+00 | 6.58E-08 | 0.00E+00 | 0.00E+00 | 7.34E-08 | 0.00E+00 | 0.00E+00 | 0.00E+00 | 0.00E+00 | 0.00E+00 | 0.00E+00 | 0.00E+00 | 0.00E+00 | 0.00E+00 | 9.09E-08 | 2.93E-07 | 0.00E+00 | 0.00E+00 | 1.45E-07 | 0.00E+00 |
| Long-term potentiation | 0.00E+00 | 0.00E+00 | 0.00E+00 | 0.00E+00 | 0.00E+00 | 0.00E+00 | 0.00E+00 | 0.00E+00 | 0.00E+00 | 0.00E+00 | 0.00E+00 | 0.00E+00 | 0.00E+00 | 0.00E+00 | 0.00E+00 | 0.00E+00 | 0.00E+00 | 0.00E+00 | 0.00E+00 | 0.00E+00 | 0.00E+00 | 0.00E+00 | 0.00E+00 | 0.00E+00 |
| Lysine biosynthesis | 5.28E-03 | 5.51E-03 | 5.63E-03 | 5.50E-03 | 5.57E-03 | 5.48E-03 | 5.55E-03 | 5.61E-03 | 5.64E-03 | 5.44E-03 | 5.43E-03 | 5.58E-03 | 5.25E-03 | 5.25E-03 | 5.19E-03 | 5.20E-03 | 5.26E-03 | 5.09E-03 | 6.15E-03 | 5.97E-03 | 6.49E-03 | 6.21E-03 | 6.34E-03 | 6.45E-03 |
| Lysine degradation | 5.62E-03 | 4.67E-03 | 4.16E-03 | 3.98E-03 | 3.03E-03 | 3.94E-03 | 4.54E-03 | 4.76E-03 | 3.82E-03 | 4.08E-03 | 3.90E-03 | 4.49E-03 | 4.00E-03 | 4.01E-03 | 3.87E-03 | 3.78E-03 | 3.88E-03 | 3.78E-03 | 5.25E-03 | 4.74E-03 | 4.16E-03 | 4.55E-03 | 4.83E-03 | 4.61E-03 |
| Lysosome | 1.77E-04 | 5.22E-04 | 3.69E-04 | 5.00E-04 | 3.87E-04 | 5.37E-04 | 5.51E-04 | 3.25E-04 | 7.16E-04 | 3.21E-04 | 2.96E-04 | 8.52E-04 | 2.38E-04 | 2.10E-04 | 2.36E-04 | 2.44E-04 | 2.56E-04 | 2.09E-04 | 2.97E-04 | 3.07E-04 | 2.32E-04 | 4.60E-04 | 2.87E-04 | 4.39E-04 |
| MAPK signaling pathway | 0.00E+00 | 2.33E-08 | 2.76E-08 | 0.00E+00 | 0.00E+00 | 6.58E-08 | 0.00E+00 | 0.00E+00 | 7.34E-08 | 0.00E+00 | 0.00E+00 | 0.00E+00 | 0.00E+00 | 0.00E+00 | 0.00E+00 | 0.00E+00 | 0.00E+00 | 0.00E+00 | 9.09E-08 | 2.93E-07 | 0.00E+00 | 0.00E+00 | 1.45E-07 | 0.00E+00 |
| MAPK signaling pathway - yeast | 3.57E-04 | 4.35E-04 | 4.15E-04 | 3.89E-04 | 3.92E-04 | 4.09E-04 | 3.97E-04 | 3.69E-04 | 4.08E-04 | 2.41E-04 | 2.79E-04 | 2.84E-04 | 2.46E-04 | 2.26E-04 | 2.34E-04 | 2.34E-04 | 2.55E-04 | 1.85E-04 | 3.16E-04 | 2.93E-04 | 3.14E-04 | 2.70E-04 | 3.61E-04 | 3.34E-04 |
| Measles | 0.00E+00 | 0.00E+00 | 0.00E+00 | 0.00E+00 | 0.00E+00 | 0.00E+00 | 0.00E+00 | 0.00E+00 | 0.00E+00 | 0.00E+00 | 0.00E+00 | 0.00E+00 | 0.00E+00 | 0.00E+00 | 0.00E+00 | 0.00E+00 | 0.00E+00 | 0.00E+00 | 0.00E+00 | 0.00E+00 | 0.00E+00 | 0.00E+00 | 0.00E+00 | 0.00E+00 |
| Meiosis - yeast | 3.18E-04 | 1.55E-04 | 3.51E-04 | 1.22E-04 | 6.96E-04 | 1.17E-04 | 2.81E-04 | 4.81E-04 | 2.07E-04 | 5.66E-05 | 4.81E-05 | 6.21E-05 | 2.16E-05 | 2.32E-05 | 2.07E-05 | 1.56E-05 | 3.28E-05 | 2.13E-05 | 2.11E-04 | 2.34E-04 | 3.92E-04 | 3.62E-04 | 3.79E-04 | 3.48E-04 |
| Melanogenesis | 1.02E-05 | 4.73E-06 | 5.52E-07 | 9.95E-06 | 1.67E-06 | 6.75E-06 | 4.95E-06 | 1.17E-05 | 4.37E-06 | 5.50E-06 | 5.78E-06 | 1.20E-06 | 3.67E-05 | 4.22E-05 | 2.22E-05 | 1.94E-05 | 2.25E-05 | 1.55E-05 | 1.50E-06 | 1.03E-05 | 2.66E-06 | 2.06E-06 | 2.31E-06 | 2.92E-06 |
| Membrane and intracellular structural molecules | 8.05E-03 | 8.77E-03 | 7.39E-03 | 9.08E-03 | 6.90E-03 | 9.14E-03 | 8.57E-03 | 8.10E-03 | 8.61E-03 | 9.01E-03 | 9.08E-03 | 8.89E-03 | 9.80E-03 | 9.78E-03 | 9.98E-03 | 1.00E-02 | 9.82E-03 | 1.02E-02 | 7.59E-03 | 8.03E-03 | 7.98E-03 | 6.74E-03 | 8.40E-03 | 8.35E-03 |
| Metabolism of cofactors and vitamins | 2.11E-03 | 2.36E-03 | 2.25E-03 | 2.37E-03 | 1.98E-03 | 2.39E-03 | 2.32E-03 | 2.18E-03 | 2.27E-03 | 2.13E-03 | 2.11E-03 | 2.42E-03 | 2.33E-03 | 2.32E-03 | 2.29E-03 | 2.29E-03 | 2.30E-03 | 2.24E-03 | 1.91E-03 | 1.92E-03 | 1.54E-03 | 1.76E-03 | 2.05E-03 | 1.67E-03 |
| Metabolism of xenobiotics by cytochrome P450 | 2.29E-03 | 1.66E-03 | 1.48E-03 | 1.29E-03 | 1.88E-03 | 1.26E-03 | 1.44E-03 | 1.76E-03 | 1.26E-03 | 1.56E-03 | 1.48E-03 | 1.14E-03 | 1.44E-03 | 1.48E-03 | 1.33E-03 | 1.29E-03 | 1.35E-03 | 1.28E-03 | 1.71E-03 | 1.42E-03 | 1.23E-03 | 1.27E-03 | 1.47E-03 | 1.24E-03 |
| Methane metabolism | 9.32E-03 | 9.48E-03 | 9.92E-03 | 9.02E-03 | 9.85E-03 | 9.03E-03 | 9.55E-03 | 9.76E-03 | 9.45E-03 | 8.85E-03 | 8.87E-03 | 9.66E-03 | 8.49E-03 | 8.49E-03 | 8.55E-03 | 8.55E-03 | 8.62E-03 | 8.53E-03 | 9.61E-03 | 9.47E-03 | 1.02E-02 | 9.35E-03 | 9.82E-03 | 1.01E-02 |
| Mineral absorption | 2.28E-04 | 1.06E-04 | 1.88E-04 | 6.48E-05 | 4.72E-04 | 6.81E-05 | 9.71E-05 | 1.59E-04 | 1.34E-04 | 8.72E-05 | 8.41E-05 | 2.33E-05 | 5.16E-05 | 5.28E-05 | 3.29E-05 | 2.96E-05 | 3.97E-05 | 2.43E-05 | 1.42E-04 | 1.03E-04 | 1.29E-04 | 1.94E-04 | 1.43E-04 | 2.01E-04 |
| Mismatch repair | 5.25E-03 | 5.59E-03 | 5.89E-03 | 5.54E-03 | 6.19E-03 | 5.60E-03 | 5.72E-03 | 5.60E-03 | 5.85E-03 | 5.68E-03 | 5.88E-03 | 5.83E-03 | 5.23E-03 | 5.20E-03 | 5.26E-03 | 5.30E-03 | 5.30E-03 | 5.23E-03 | 6.13E-03 | 5.85E-03 | 6.24E-03 | 6.20E-03 | 6.28E-03 | 6.41E-03 |
| N-Glycan biosynthesis | 1.12E-04 | 8.95E-05 | 2.33E-04 | 8.08E-05 | 5.18E-04 | 8.72E-05 | 1.42E-04 | 2.01E-04 | 1.96E-04 | 6.57E-05 | 4.96E-05 | 6.05E-05 | 2.49E-05 | 2.29E-05 | 3.00E-05 | 2.54E-05 | 3.69E-05 | 3.12E-05 | 1.99E-04 | 2.08E-04 | 3.64E-04 | 2.65E-04 | 2.05E-04 | 3.36E-04 |
| NOD-like receptor signaling pathway | 1.86E-04 | 3.67E-04 | 3.44E-04 | 3.01E-04 | 3.22E-04 | 3.19E-04 | 3.07E-04 | 2.61E-04 | 3.06E-04 | 2.62E-04 | 2.76E-04 | 4.88E-04 | 2.70E-04 | 2.67E-04 | 2.72E-04 | 2.76E-04 | 2.76E-04 | 2.58E-04 | 2.59E-04 | 2.61E-04 | 3.22E-04 | 2.22E-04 | 2.88E-04 | 3.21E-04 |
| Naphthalene degradation | 2.59E-03 | 2.39E-03 | 2.01E-03 | 1.83E-03 | 1.86E-03 | 1.83E-03 | 2.14E-03 | 2.20E-03 | 1.88E-03 | 2.12E-03 | 2.06E-03 | 2.07E-03 | 1.65E-03 | 1.64E-03 | 1.53E-03 | 1.47E-03 | 1.57E-03 | 1.41E-03 | 2.70E-03 | 2.03E-03 | 2.00E-03 | 1.76E-03 | 1.91E-03 | 2.32E-03 |
| Neuroactive ligand-receptor interaction | 1.63E-07 | 8.16E-07 | 8.00E-07 | 4.39E-07 | 4.59E-07 | 7.24E-07 | 3.33E-07 | 4.98E-08 | 1.10E-07 | 3.35E-08 | 1.14E-07 | 1.72E-07 | 6.30E-08 | 0.00E+00 | 5.25E-08 | 3.60E-08 | 0.00E+00 | 0.00E+00 | 0.00E+00 | 0.00E+00 | 0.00E+00 | 0.00E+00 | 3.25E-07 | 2.44E-07 |
| Neurotrophin signaling pathway | 0.00E+00 | 0.00E+00 | 0.00E+00 | 0.00E+00 | 0.00E+00 | 0.00E+00 | 0.00E+00 | 0.00E+00 | 0.00E+00 | 0.00E+00 | 0.00E+00 | 0.00E+00 | 0.00E+00 | 0.00E+00 | 0.00E+00 | 0.00E+00 | 0.00E+00 | 0.00E+00 | 0.00E+00 | 0.00E+00 | 0.00E+00 | 0.00E+00 | 0.00E+00 | 0.00E+00 |
| Nicotinate and nicotinamide metabolism | 4.19E-03 | 4.49E-03 | 4.40E-03 | 4.51E-03 | 4.33E-03 | 4.54E-03 | 4.55E-03 | 4.44E-03 | 4.51E-03 | 4.44E-03 | 4.31E-03 | 4.86E-03 | 4.39E-03 | 4.38E-03 | 4.41E-03 | 4.46E-03 | 4.42E-03 | 4.39E-03 | 4.60E-03 | 4.46E-03 | 4.28E-03 | 4.79E-03 | 4.62E-03 | 4.36E-03 |
| Nitrogen metabolism | 8.54E-03 | 8.37E-03 | 7.74E-03 | 8.73E-03 | 7.35E-03 | 8.67E-03 | 8.42E-03 | 8.10E-03 | 8.77E-03 | 8.48E-03 | 8.26E-03 | 8.03E-03 | 9.10E-03 | 9.24E-03 | 9.10E-03 | 9.12E-03 | 9.02E-03 | 9.19E-03 | 7.42E-03 | 7.94E-03 | 7.95E-03 | 7.47E-03 | 7.52E-03 | 7.82E-03 |
| Nitrotoluene degradation | 9.61E-04 | 8.72E-04 | 9.63E-04 | 1.02E-03 | 7.08E-04 | 1.02E-03 | 9.73E-04 | 1.05E-03 | 9.96E-04 | 9.94E-04 | 1.01E-03 | 8.50E-04 | 1.03E-03 | 1.03E-03 | 1.15E-03 | 1.17E-03 | 1.11E-03 | 1.25E-03 | 1.07E-03 | 1.01E-03 | 8.33E-04 | 8.06E-04 | 9.66E-04 | 8.86E-04 |
| Non-homologous end-joining | 4.70E-04 | 2.63E-04 | 2.48E-04 | 1.76E-04 | 1.04E-04 | 1.61E-04 | 1.73E-04 | 1.48E-04 | 1.44E-04 | 1.06E-04 | 9.27E-05 | 2.68E-04 | 1.07E-04 | 1.09E-04 | 7.51E-05 | 6.85E-05 | 8.87E-05 | 4.76E-05 | 3.34E-04 | 2.68E-04 | 1.60E-04 | 3.74E-04 | 2.41E-04 | 2.28E-04 |
| Notch signaling pathway | 1.31E-07 | 3.27E-07 | 7.73E-07 | 2.93E-07 | 1.25E-07 | 1.97E-07 | 7.62E-07 | 3.99E-07 | 2.20E-07 | 3.35E-08 | 5.69E-08 | 6.87E-07 | 2.52E-07 | 1.20E-07 | 4.73E-07 | 2.16E-07 | 2.30E-07 | 0.00E+00 | 7.27E-07 | 8.80E-07 | 0.00E+00 | 6.19E-07 | 3.97E-07 | 3.05E-07 |
| Novobiocin biosynthesis | 1.23E-03 | 1.22E-03 | 1.22E-03 | 1.21E-03 | 1.17E-03 | 1.21E-03 | 1.22E-03 | 1.24E-03 | 1.21E-03 | 1.19E-03 | 1.16E-03 | 1.27E-03 | 1.21E-03 | 1.22E-03 | 1.21E-03 | 1.22E-03 | 1.22E-03 | 1.20E-03 | 1.30E-03 | 1.31E-03 | 1.50E-03 | 1.45E-03 | 1.39E-03 | 1.53E-03 |
| Nucleotide excision repair | 2.50E-03 | 2.24E-03 | 2.50E-03 | 2.09E-03 | 2.58E-03 | 2.07E-03 | 2.26E-03 | 2.35E-03 | 2.23E-03 | 2.37E-03 | 2.36E-03 | 2.34E-03 | 1.95E-03 | 1.96E-03 | 1.93E-03 | 1.93E-03 | 1.97E-03 | 1.90E-03 | 2.95E-03 | 2.73E-03 | 3.12E-03 | 3.29E-03 | 2.97E-03 | 3.10E-03 |
| Nucleotide metabolism | 6.17E-04 | 4.10E-04 | 4.11E-04 | 7.45E-04 | 3.00E-04 | 7.44E-04 | 5.29E-04 | 6.16E-04 | 5.94E-04 | 1.23E-03 | 1.26E-03 | 5.61E-04 | 1.27E-03 | 1.28E-03 | 1.52E-03 | 1.54E-03 | 1.39E-03 | 1.79E-03 | 4.14E-04 | 7.99E-04 | 7.02E-04 | 5.21E-04 | 4.77E-04 | 5.80E-04 |
| Olfactory transduction | 0.00E+00 | 0.00E+00 | 0.00E+00 | 0.00E+00 | 0.00E+00 | 0.00E+00 | 0.00E+00 | 0.00E+00 | 0.00E+00 | 0.00E+00 | 0.00E+00 | 0.00E+00 | 0.00E+00 | 0.00E+00 | 0.00E+00 | 0.00E+00 | 0.00E+00 | 0.00E+00 | 0.00E+00 | 0.00E+00 | 0.00E+00 | 0.00E+00 | 0.00E+00 | 0.00E+00 |
| One carbon pool by folate | 4.40E-03 | 4.20E-03 | 4.52E-03 | 4.12E-03 | 4.53E-03 | 4.13E-03 | 4.43E-03 | 4.63E-03 | 4.40E-03 | 4.45E-03 | 4.42E-03 | 4.09E-03 | 4.01E-03 | 4.03E-03 | 3.98E-03 | 3.98E-03 | 4.01E-03 | 3.96E-03 | 4.71E-03 | 4.64E-03 | 5.17E-03 | 4.94E-03 | 5.00E-03 | 5.23E-03 |
| Oocyte meiosis | 0.00E+00 | 0.00E+00 | 0.00E+00 | 0.00E+00 | 0.00E+00 | 0.00E+00 | 0.00E+00 | 0.00E+00 | 0.00E+00 | 0.00E+00 | 0.00E+00 | 0.00E+00 | 0.00E+00 | 0.00E+00 | 0.00E+00 | 0.00E+00 | 0.00E+00 | 0.00E+00 | 0.00E+00 | 0.00E+00 | 0.00E+00 | 0.00E+00 | 0.00E+00 | 0.00E+00 |
| Other glycan degradation | 3.70E-04 | 8.77E-04 | 7.36E-04 | 9.48E-04 | 9.84E-04 | 1.06E-03 | 1.05E-03 | 6.82E-04 | 1.62E-03 | 7.06E-04 | 6.94E-04 | 1.23E-03 | 5.50E-04 | 4.87E-04 | 5.95E-04 | 5.94E-04 | 6.04E-04 | 6.06E-04 | 6.05E-04 | 7.11E-04 | 6.47E-04 | 9.46E-04 | 5.98E-04 | 8.96E-04 |
| Other ion-coupled transporters | 1.26E-02 | 1.48E-02 | 1.27E-02 | 1.59E-02 | 1.18E-02 | 1.61E-02 | 1.46E-02 | 1.35E-02 | 1.53E-02 | 1.78E-02 | 1.73E-02 | 1.68E-02 | 1.80E-02 | 1.81E-02 | 1.85E-02 | 1.85E-02 | 1.80E-02 | 1.91E-02 | 1.37E-02 | 1.44E-02 | 1.43E-02 | 1.28E-02 | 1.22E-02 | 1.37E-02 |
| Other transporters | 2.50E-03 | 3.08E-03 | 2.83E-03 | 3.05E-03 | 2.74E-03 | 3.07E-03 | 2.84E-03 | 2.69E-03 | 2.88E-03 | 3.12E-03 | 3.05E-03 | 3.04E-03 | 3.20E-03 | 3.22E-03 | 3.17E-03 | 3.22E-03 | 3.18E-03 | 3.11E-03 | 2.79E-03 | 2.63E-03 | 2.60E-03 | 2.37E-03 | 2.65E-03 | 2.73E-03 |
| Other types of O-glycan biosynthesis | 0.00E+00 | 0.00E+00 | 0.00E+00 | 0.00E+00 | 0.00E+00 | 0.00E+00 | 0.00E+00 | 0.00E+00 | 0.00E+00 | 0.00E+00 | 0.00E+00 | 0.00E+00 | 0.00E+00 | 0.00E+00 | 0.00E+00 | 0.00E+00 | 0.00E+00 | 0.00E+00 | 0.00E+00 | 0.00E+00 | 0.00E+00 | 0.00E+00 | 0.00E+00 | 0.00E+00 |
| Others | 9.95E-03 | 9.33E-03 | 9.69E-03 | 9.76E-03 | 1.15E-02 | 9.66E-03 | 9.72E-03 | 1.04E-02 | 9.94E-03 | 1.07E-02 | 1.10E-02 | 9.89E-03 | 1.10E-02 | 1.11E-02 | 1.12E-02 | 1.13E-02 | 1.10E-02 | 1.17E-02 | 8.60E-03 | 9.74E-03 | 9.67E-03 | 1.00E-02 | 8.51E-03 | 1.30E-02 |
| Oxidative phosphorylation | 1.19E-02 | 1.12E-02 | 1.23E-02 | 1.08E-02 | 1.26E-02 | 1.07E-02 | 1.12E-02 | 1.18E-02 | 1.11E-02 | 1.10E-02 | 1.02E-02 | 1.09E-02 | 1.01E-02 | 1.01E-02 | 9.93E-03 | 9.91E-03 | 1.01E-02 | 9.78E-03 | 1.29E-02 | 1.22E-02 | 1.28E-02 | 1.21E-02 | 1.44E-02 | 1.29E-02 |
| PPAR signaling pathway | 1.70E-03 | 1.47E-03 | 1.62E-03 | 1.09E-03 | 1.44E-03 | 1.10E-03 | 1.48E-03 | 1.52E-03 | 1.26E-03 | 1.14E-03 | 1.03E-03 | 1.12E-03 | 8.11E-04 | 7.95E-04 | 7.31E-04 | 6.98E-04 | 7.86E-04 | 6.33E-04 | 1.81E-03 | 1.49E-03 | 1.30E-03 | 1.66E-03 | 1.65E-03 | 1.24E-03 |
| Pancreatic cancer | 0.00E+00 | 0.00E+00 | 0.00E+00 | 0.00E+00 | 0.00E+00 | 0.00E+00 | 0.00E+00 | 0.00E+00 | 0.00E+00 | 0.00E+00 | 0.00E+00 | 0.00E+00 | 0.00E+00 | 0.00E+00 | 0.00E+00 | 0.00E+00 | 0.00E+00 | 0.00E+00 | 0.00E+00 | 0.00E+00 | 0.00E+00 | 0.00E+00 | 0.00E+00 | 0.00E+00 |
| Pancreatic secretion | 1.24E-06 | 1.89E-06 | 1.10E-06 | 5.85E-07 | 6.68E-07 | 1.02E-06 | 4.29E-07 | 2.99E-07 | 3.30E-07 | 3.35E-08 | 1.42E-07 | 4.01E-07 | 1.26E-07 | 0.00E+00 | 1.05E-07 | 7.20E-08 | 0.00E+00 | 0.00E+00 | 3.18E-07 | 8.80E-07 | 1.83E-06 | 1.34E-06 | 1.88E-06 | 1.83E-06 |
| Pantothenate and CoA biosynthesis | 4.99E-03 | 4.53E-03 | 4.93E-03 | 4.56E-03 | 5.43E-03 | 4.52E-03 | 4.77E-03 | 5.14E-03 | 4.77E-03 | 4.96E-03 | 4.99E-03 | 4.47E-03 | 4.59E-03 | 4.61E-03 | 4.69E-03 | 4.71E-03 | 4.68E-03 | 4.78E-03 | 5.34E-03 | 5.23E-03 | 5.62E-03 | 5.48E-03 | 5.37E-03 | 5.56E-03 |
| Parkinson's disease | 7.72E-04 | 6.37E-04 | 4.58E-04 | 4.92E-04 | 3.09E-04 | 4.90E-04 | 6.10E-04 | 5.64E-04 | 4.76E-04 | 1.96E-04 | 1.84E-04 | 5.86E-04 | 2.45E-04 | 2.29E-04 | 1.75E-04 | 1.56E-04 | 2.08E-04 | 1.02E-04 | 3.62E-04 | 2.98E-04 | 2.09E-04 | 3.56E-04 | 5.96E-04 | 3.18E-04 |
| Pathogenic Escherichia coli infection | 9.01E-06 | 5.48E-06 | 9.71E-06 | 3.91E-05 | 1.23E-05 | 3.74E-05 | 2.31E-05 | 3.65E-05 | 2.70E-05 | 1.45E-04 | 1.37E-04 | 3.95E-05 | 1.20E-04 | 1.32E-04 | 1.41E-04 | 1.56E-04 | 1.31E-04 | 1.14E-04 | 6.41E-06 | 4.11E-05 | 2.41E-05 | 1.39E-05 | 1.09E-05 | 1.15E-05 |
| Pathways in cancer | 6.97E-04 | 5.04E-04 | 5.34E-04 | 5.18E-04 | 5.97E-04 | 5.02E-04 | 5.69E-04 | 6.39E-04 | 5.23E-04 | 5.31E-04 | 5.06E-04 | 4.83E-04 | 5.21E-04 | 5.29E-04 | 5.12E-04 | 5.16E-04 | 5.15E-04 | 4.99E-04 | 5.29E-04 | 5.35E-04 | 5.26E-04 | 5.61E-04 | 6.16E-04 | 5.83E-04 |
| Penicillin and cephalosporin biosynthesis | 4.94E-04 | 4.44E-04 | 3.80E-04 | 3.54E-04 | 3.63E-04 | 3.55E-04 | 3.65E-04 | 3.49E-04 | 3.36E-04 | 3.39E-04 | 3.35E-04 | 3.48E-04 | 2.99E-04 | 2.96E-04 | 2.82E-04 | 2.73E-04 | 2.92E-04 | 2.58E-04 | 5.19E-04 | 4.16E-04 | 3.82E-04 | 5.07E-04 | 4.14E-04 | 4.36E-04 |
| Pentose and glucuronate interconversions | 3.82E-03 | 3.49E-03 | 3.46E-03 | 3.99E-03 | 3.48E-03 | 4.00E-03 | 3.92E-03 | 3.86E-03 | 4.17E-03 | 4.17E-03 | 4.25E-03 | 3.87E-03 | 4.20E-03 | 4.10E-03 | 4.52E-03 | 4.49E-03 | 4.38E-03 | 4.77E-03 | 3.50E-03 | 4.13E-03 | 3.74E-03 | 4.34E-03 | 3.32E-03 | 3.96E-03 |
| Pentose phosphate pathway | 6.32E-03 | 6.27E-03 | 6.79E-03 | 6.77E-03 | 7.47E-03 | 6.81E-03 | 6.65E-03 | 6.66E-03 | 6.92E-03 | 6.73E-03 | 6.75E-03 | 6.66E-03 | 6.86E-03 | 6.84E-03 | 6.93E-03 | 6.99E-03 | 6.93E-03 | 6.96E-03 | 6.16E-03 | 6.68E-03 | 5.96E-03 | 7.10E-03 | 6.24E-03 | 6.23E-03 |
| Peptidases | 1.39E-02 | 1.44E-02 | 1.53E-02 | 1.42E-02 | 1.76E-02 | 1.43E-02 | 1.44E-02 | 1.48E-02 | 1.49E-02 | 1.46E-02 | 1.48E-02 | 1.53E-02 | 1.41E-02 | 1.40E-02 | 1.43E-02 | 1.43E-02 | 1.43E-02 | 1.45E-02 | 1.47E-02 | 1.52E-02 | 1.66E-02 | 1.49E-02 | 1.56E-02 | 1.66E-02 |

| | | | | | | | | | | | | | | | | | | | | | | | | |
|---|---|---|---|---|---|---|---|---|---|---|---|---|---|---|---|---|---|---|---|---|---|---|---|---|
| Peptidoglycan biosynthesis | 5.58E-03 | 5.83E-03 | 6.12E-03 | 5.76E-03 | 6.19E-03 | 5.71E-03 | 5.87E-03 | 5.99E-03 | 5.89E-03 | 6.06E-03 | 6.31E-03 | 6.19E-03 | 5.65E-03 | 5.67E-03 | 5.67E-03 | 5.68E-03 | 5.70E-03 | 5.67E-03 | 6.48E-03 | 6.39E-03 | 7.26E-03 | 6.63E-03 | 6.73E-03 | 7.10E-03 |
| Peroxisome | 2.67E-03 | 2.62E-03 | 2.45E-03 | 2.33E-03 | 2.29E-03 | 2.34E-03 | 2.54E-03 | 2.44E-03 | 2.47E-03 | 2.13E-03 | 1.98E-03 | 2.32E-03 | 1.89E-03 | 1.87E-03 | 1.79E-03 | 1.78E-03 | 1.86E-03 | 1.63E-03 | 2.69E-03 | 2.38E-03 | 2.30E-03 | 2.64E-03 | 2.55E-03 | 2.48E-03 |
| Pertussis | 9.84E-04 | 7.73E-04 | 6.64E-04 | 1.37E-03 | 6.61E-04 | 1.31E-03 | 8.82E-04 | 1.10E-03 | 1.07E-03 | 1.69E-03 | 1.68E-03 | 7.21E-04 | 2.58E-03 | 2.73E-03 | 2.57E-03 | 2.62E-03 | 2.47E-03 | 2.75E-03 | 6.81E-04 | 1.29E-03 | 8.94E-04 | 7.68E-04 | 6.35E-04 | 8.48E-04 |
| Phagosome | 0.00E+00 | 0.00E+00 | 0.00E+00 | 1.46E-07 | 4.17E-08 | 3.29E-08 | 0.00E+00 | 9.96E-08 | 0.00E+00 | 0.00E+00 | 0.00E+00 | 0.00E+00 | 0.00E+00 | 0.00E+00 | 0.00E+00 | 3.60E-08 | 0.00E+00 | 0.00E+00 | 4.55E-08 | 0.00E+00 | 0.00E+00 | 5.15E-07 | 1.37E-06 | 1.83E-07 |
| Phenylalanine metabolism | 3.75E-03 | 2.73E-03 | 2.75E-03 | 2.56E-03 | 2.43E-03 | 2.52E-03 | 2.88E-03 | 3.15E-03 | 2.62E-03 | 2.61E-03 | 2.51E-03 | 2.35E-03 | 2.83E-03 | 2.86E-03 | 2.78E-03 | 2.68E-03 | 2.73E-03 | 2.90E-03 | 3.23E-03 | 3.19E-03 | 3.01E-03 | 3.11E-03 | 2.97E-03 | 3.24E-03 |
| Phenylalanine, tyrosine and tryptophan biosynthesis | 5.96E-03 | 6.16E-03 | 6.37E-03 | 5.99E-03 | 6.65E-03 | 5.99E-03 | 6.22E-03 | 6.29E-03 | 6.25E-03 | 6.33E-03 | 6.26E-03 | 6.06E-03 | 5.79E-03 | 5.77E-03 | 5.76E-03 | 5.76E-03 | 5.81E-03 | 5.72E-03 | 7.00E-03 | 6.55E-03 | 7.10E-03 | 7.13E-03 | 6.99E-03 | 7.11E-03 |
| Phenylpropanoid biosynthesis | 7.00E-04 | 7.03E-04 | 8.61E-04 | 6.92E-04 | 1.11E-03 | 7.21E-04 | 8.47E-04 | 7.97E-04 | 1.03E-03 | 6.09E-04 | 6.59E-04 | 7.02E-04 | 5.65E-04 | 5.24E-04 | 6.00E-04 | 5.73E-04 | 6.00E-04 | 6.05E-04 | 8.79E-04 | 9.11E-04 | 7.21E-04 | 9.70E-04 | 8.43E-04 | 9.19E-04 |
| Phosphatidylinositol signaling system | 9.49E-04 | 8.84E-04 | 1.03E-03 | 9.13E-04 | 1.13E-03 | 9.15E-04 | 9.70E-04 | 1.02E-03 | 1.00E-03 | 9.10E-04 | 9.39E-04 | 7.93E-04 | 9.25E-04 | 9.31E-04 | 9.09E-04 | 9.16E-04 | 9.09E-04 | 9.10E-04 | 9.74E-04 | 9.40E-04 | 9.58E-04 | 9.57E-04 | 1.00E-03 | 9.45E-04 |
| Phosphonate and phosphinate metabolism | 7.16E-04 | 7.16E-04 | 5.80E-04 | 7.76E-04 | 4.30E-04 | 7.68E-04 | 7.22E-04 | 6.31E-04 | 6.95E-04 | 5.74E-04 | 5.96E-04 | 6.33E-04 | 6.57E-04 | 6.44E-04 | 6.15E-04 | 5.92E-04 | 6.30E-04 | 5.80E-04 | 5.59E-04 | 5.75E-04 | 6.00E-04 | 5.63E-04 | 4.42E-04 | 5.59E-04 |
| Phosphotransferase system (PTS) | 3.00E-03 | 4.35E-03 | 3.56E-03 | 6.04E-03 | 2.94E-03 | 6.11E-03 | 4.62E-03 | 4.08E-03 | 5.06E-03 | 6.90E-03 | 7.66E-03 | 5.71E-03 | 7.61E-03 | 7.58E-03 | 8.31E-03 | 8.56E-03 | 8.03E-03 | 8.92E-03 | 2.91E-03 | 4.59E-03 | 3.80E-03 | 4.10E-03 | 2.55E-03 | 2.91E-03 |
| Photosynthesis | 2.54E-03 | 3.61E-03 | 6.68E-03 | 3.18E-03 | 1.15E-02 | 3.36E-03 | 3.78E-03 | 4.39E-03 | 4.33E-03 | 2.44E-03 | 2.45E-03 | 3.23E-03 | 2.06E-03 | 1.99E-03 | 1.99E-03 | 2.02E-03 | 2.14E-03 | 1.95E-03 | 2.79E-03 | 2.88E-03 | 2.60E-03 | 3.17E-03 | 3.16E-03 | 2.41E-03 |
| Photosynthesis - antenna proteins | 1.07E-04 | 2.96E-04 | 1.28E-03 | 1.97E-04 | 2.53E-03 | 2.37E-04 | 3.05E-04 | 5.51E-04 | 4.79E-04 | 4.31E-05 | 2.86E-05 | 5.74E-05 | 3.32E-05 | 2.90E-05 | 1.77E-05 | 2.06E-05 | 3.89E-05 | 2.39E-05 | 5.08E-05 | 1.54E-04 | 1.62E-04 | 1.32E-04 | 1.42E-04 | 4.97E-05 |
| Photosynthesis proteins | 3.12E-03 | 4.16E-03 | 8.24E-03 | 3.62E-03 | 1.43E-02 | 3.84E-03 | 4.34E-03 | 5.23E-03 | 5.04E-03 | 2.70E-03 | 2.69E-03 | 3.43E-03 | 2.29E-03 | 2.21E-03 | 2.22E-03 | 2.25E-03 | 2.38E-03 | 2.19E-03 | 3.06E-03 | 3.22E-03 | 2.93E-03 | 3.48E-03 | 3.55E-03 | 2.63E-03 |
| Phototransduction | 0.00E+00 | 0.00E+00 | 0.00E+00 | 0.00E+00 | 0.00E+00 | 0.00E+00 | 0.00E+00 | 0.00E+00 | 0.00E+00 | 0.00E+00 | 0.00E+00 | 0.00E+00 | 0.00E+00 | 0.00E+00 | 0.00E+00 | 0.00E+00 | 0.00E+00 | 0.00E+00 | 0.00E+00 | 0.00E+00 | 0.00E+00 | 0.00E+00 | 0.00E+00 | 0.00E+00 |
| Phototransduction - fly | 0.00E+00 | 0.00E+00 | 0.00E+00 | 1.46E-07 | 4.17E-08 | 3.29E-08 | 0.00E+00 | 9.96E-08 | 0.00E+00 | 0.00E+00 | 0.00E+00 | 0.00E+00 | 0.00E+00 | 0.00E+00 | 0.00E+00 | 3.60E-08 | 0.00E+00 | 0.00E+00 | 4.55E-08 | 0.00E+00 | 0.00E+00 | 5.15E-07 | 1.37E-06 | 1.83E-07 |
| Plant-pathogen interaction | 1.25E-03 | 1.64E-03 | 1.64E-03 | 1.62E-03 | 1.23E-03 | 1.64E-03 | 1.61E-03 | 1.38E-03 | 1.58E-03 | 1.19E-03 | 1.23E-03 | 1.66E-03 | 1.20E-03 | 1.14E-03 | 1.20E-03 | 1.19E-03 | 1.23E-03 | 1.13E-03 | 1.47E-03 | 1.34E-03 | 1.42E-03 | 1.32E-03 | 1.56E-03 | 1.43E-03 |
| Polycyclic aromatic hydrocarbon degradation | 1.08E-03 | 7.83E-04 | 9.45E-04 | 5.59E-04 | 1.01E-03 | 5.18E-04 | 7.65E-04 | 9.47E-04 | 6.62E-04 | 7.14E-04 | 7.10E-04 | 5.92E-04 | 4.95E-04 | 5.15E-04 | 3.69E-04 | 3.34E-04 | 4.09E-04 | 2.65E-04 | 1.14E-03 | 8.71E-04 | 6.43E-04 | 9.70E-04 | 7.70E-04 | 7.93E-04 |
| Polyketide sugar unit biosynthesis | 1.13E-03 | 8.43E-04 | 1.08E-03 | 8.63E-04 | 1.63E-03 | 8.40E-04 | 1.08E-03 | 1.20E-03 | 1.18E-03 | 8.88E-04 | 9.13E-04 | 8.22E-04 | 8.15E-04 | 8.38E-04 | 8.41E-04 | 8.11E-04 | 8.33E-04 | 9.18E-04 | 1.23E-03 | 1.32E-03 | 1.72E-03 | 1.42E-03 | 1.46E-03 | 1.88E-03 |
| Pores ion channels | 6.40E-03 | 6.36E-03 | 5.41E-03 | 6.44E-03 | 6.11E-03 | 6.52E-03 | 6.08E-03 | 5.82E-03 | 6.54E-03 | 6.90E-03 | 6.72E-03 | 5.94E-03 | 7.34E-03 | 7.40E-03 | 7.23E-03 | 7.22E-03 | 7.16E-03 | 7.21E-03 | 5.87E-03 | 5.95E-03 | 5.81E-03 | 4.91E-03 | 6.19E-03 | 6.31E-03 |
| Porphyrin and chlorophyll metabolism | 9.21E-03 | 8.53E-03 | 1.10E-02 | 8.16E-03 | 1.36E-02 | 8.14E-03 | 8.63E-03 | 9.55E-03 | 9.21E-03 | 7.60E-03 | 6.76E-03 | 8.23E-03 | 7.07E-03 | 7.09E-03 | 6.73E-03 | 6.68E-03 | 6.91E-03 | 6.45E-03 | 7.88E-03 | 8.06E-03 | 8.08E-03 | 7.84E-03 | 8.91E-03 | 8.02E-03 |
| Prenyltransferases | 2.43E-03 | 2.45E-03 | 3.15E-03 | 2.37E-03 | 3.79E-03 | 2.38E-03 | 2.56E-03 | 2.74E-03 | 2.67E-03 | 2.48E-03 | 2.40E-03 | 2.38E-03 | 2.11E-03 | 2.09E-03 | 2.10E-03 | 2.10E-03 | 2.14E-03 | 2.08E-03 | 3.11E-03 | 2.91E-03 | 3.23E-03 | 3.54E-03 | 3.27E-03 | 3.26E-03 |
| Primary bile acid biosynthesis | 1.74E-04 | 2.20E-04 | 1.73E-04 | 1.67E-04 | 9.71E-05 | 1.79E-04 | 1.74E-04 | 1.35E-04 | 1.66E-04 | 1.17E-04 | 1.18E-04 | 3.07E-04 | 1.38E-04 | 1.40E-04 | 1.18E-04 | 1.20E-04 | 1.25E-04 | 9.71E-05 | 1.75E-04 | 1.65E-04 | 1.03E-04 | 1.14E-04 | 1.14E-04 | 8.62E-05 |
| Primary immunodeficiency | 4.73E-04 | 5.82E-04 | 5.11E-04 | 5.45E-04 | 4.53E-04 | 5.52E-04 | 5.42E-04 | 4.62E-04 | 5.30E-04 | 5.49E-04 | 5.38E-04 | 6.57E-04 | 4.71E-04 | 4.60E-04 | 4.69E-04 | 4.70E-04 | 4.77E-04 | 4.60E-04 | 5.38E-04 | 5.00E-04 | 4.96E-04 | 5.79E-04 | 4.37E-04 | 4.52E-04 |
| Prion diseases | 1.14E-04 | 1.10E-04 | 8.02E-05 | 1.19E-04 | 5.04E-05 | 1.21E-04 | 1.04E-04 | 9.50E-05 | 1.06E-04 | 1.72E-04 | 1.68E-04 | 1.78E-04 | 1.84E-04 | 1.86E-04 | 2.06E-04 | 2.18E-04 | 1.93E-04 | 2.20E-04 | 9.37E-05 | 1.23E-04 | 8.11E-05 | 8.81E-05 | 1.01E-04 | 1.26E-04 |
| Progesterone-mediated oocyte maturation | 1.76E-04 | 2.48E-04 | 2.70E-04 | 2.45E-04 | 2.88E-04 | 2.48E-04 | 2.47E-04 | 2.39E-04 | 2.61E-04 | 2.43E-04 | 2.51E-04 | 2.55E-04 | 2.56E-04 | 2.57E-04 | 2.61E-04 | 2.63E-04 | 2.60E-04 | 2.54E-04 | 2.36E-04 | 2.44E-04 | 3.03E-04 | 1.99E-04 | 2.71E-04 | 3.05E-04 |
| Propanoate metabolism | 9.93E-03 | 8.74E-03 | 8.08E-03 | 7.85E-03 | 6.28E-03 | 7.77E-03 | 8.57E-03 | 8.58E-03 | 7.69E-03 | 8.00E-03 | 7.77E-03 | 8.02E-03 | 7.63E-03 | 7.70E-03 | 7.35E-03 | 7.26E-03 | 7.43E-03 | 7.04E-03 | 9.55E-03 | 8.45E-03 | 7.47E-03 | 8.36E-03 | 8.55E-03 | 7.71E-03 |
| Prostate cancer | 1.76E-04 | 2.48E-04 | 2.70E-04 | 2.45E-04 | 2.89E-04 | 2.48E-04 | 2.48E-04 | 2.39E-04 | 2.62E-04 | 2.43E-04 | 2.51E-04 | 2.55E-04 | 2.56E-04 | 2.57E-04 | 2.61E-04 | 2.63E-04 | 2.60E-04 | 2.54E-04 | 2.36E-04 | 2.44E-04 | 3.04E-04 | 1.99E-04 | 2.72E-04 | 3.05E-04 |
| Proteasome | 2.64E-04 | 2.75E-04 | 3.55E-04 | 2.59E-04 | 2.98E-04 | 2.53E-04 | 2.62E-04 | 2.59E-04 | 2.66E-04 | 3.39E-04 | 2.85E-04 | 3.18E-04 | 2.57E-04 | 2.59E-04 | 2.63E-04 | 2.65E-04 | 2.63E-04 | 2.55E-04 | 3.00E-04 | 3.34E-04 | 3.58E-04 | 2.31E-04 | 3.02E-04 | 3.19E-04 |
| Protein digestion and absorption | 5.58E-05 | 3.91E-05 | 4.25E-05 | 2.15E-05 | 4.67E-05 | 2.54E-05 | 4.25E-05 | 3.77E-05 | 5.07E-05 | 2.10E-05 | 2.63E-05 | 3.54E-05 | 5.48E-06 | 3.54E-06 | 5.46E-06 | 3.67E-06 | 6.21E-06 | 1.70E-06 | 5.92E-05 | 7.13E-05 | 3.95E-05 | 4.61E-05 | 8.90E-05 | 1.32E-04 |
| Protein export | 4.34E-03 | 4.46E-03 | 4.78E-03 | 4.22E-03 | 4.86E-03 | 4.28E-03 | 4.52E-03 | 4.56E-03 | 4.53E-03 | 4.67E-03 | 4.68E-03 | 4.41E-03 | 3.93E-03 | 3.87E-03 | 3.99E-03 | 4.02E-03 | 4.02E-03 | 3.99E-03 | 5.26E-03 | 4.84E-03 | 5.28E-03 | 5.68E-03 | 5.44E-03 | 5.27E-03 |
| Protein folding and associated processing | 7.83E-03 | 7.97E-03 | 8.27E-03 | 8.00E-03 | 8.88E-03 | 7.98E-03 | 8.05E-03 | 8.15E-03 | 8.28E-03 | 8.02E-03 | 7.86E-03 | 7.76E-03 | 8.06E-03 | 8.07E-03 | 8.09E-03 | 8.11E-03 | 8.09E-03 | 8.17E-03 | 7.57E-03 | 7.60E-03 | 7.16E-03 | 7.65E-03 | 8.45E-03 | 7.58E-03 |
| Protein kinases | 4.65E-03 | 5.20E-03 | 5.17E-03 | 5.45E-03 | 5.81E-03 | 5.50E-03 | 4.91E-03 | 4.85E-03 | 5.24E-03 | 5.28E-03 | 5.19E-03 | 5.06E-03 | 5.76E-03 | 5.78E-03 | 5.73E-03 | 5.75E-03 | 5.70E-03 | 5.73E-03 | 4.38E-03 | 4.83E-03 | 5.09E-03 | 4.46E-03 | 4.09E-03 | 4.28E-03 |
| Protein processing in endoplasmic reticulum | 3.68E-04 | 3.86E-04 | 5.71E-04 | 3.57E-04 | 8.22E-04 | 3.67E-04 | 4.47E-04 | 4.96E-04 | 5.12E-04 | 3.11E-04 | 2.91E-04 | 3.17E-04 | 3.19E-04 | 3.21E-04 | 3.06E-04 | 3.02E-04 | 3.17E-04 | 2.85E-04 | 5.65E-04 | 5.18E-04 | 6.94E-04 | 5.33E-04 | 7.73E-04 | 7.56E-04 |
| Proximal tubule bicarbonate reclamation | 3.69E-04 | 3.32E-04 | 2.72E-04 | 2.73E-04 | 2.92E-04 | 2.70E-04 | 2.66E-04 | 2.95E-04 | 2.49E-04 | 3.76E-04 | 3.66E-04 | 3.76E-04 | 3.05E-04 | 3.06E-04 | 3.20E-04 | 3.20E-04 | 3.11E-04 | 3.40E-04 | 4.07E-04 | 3.31E-04 | 3.50E-04 | 3.50E-04 | 2.81E-04 | 3.53E-04 |
| Purine metabolism | 1.87E-02 | 1.92E-02 | 1.96E-02 | 1.92E-02 | 1.98E-02 | 1.92E-02 | 1.94E-02 | 1.94E-02 | 1.94E-02 | 1.98E-02 | 1.99E-02 | 2.04E-02 | 1.86E-02 | 1.85E-02 | 1.88E-02 | 1.89E-02 | 1.88E-02 | 1.89E-02 | 1.98E-02 | 1.95E-02 | 2.04E-02 | 2.05E-02 | 2.04E-02 | 2.04E-02 |
| Pyrimidine metabolism | 1.19E-02 | 1.28E-02 | 1.33E-02 | 1.28E-02 | 1.34E-02 | 1.29E-02 | 1.32E-02 | 1.29E-02 | 1.33E-02 | 1.32E-02 | 1.35E-02 | 1.39E-02 | 1.18E-02 | 1.17E-02 | 1.20E-02 | 1.21E-02 | 1.21E-02 | 1.19E-02 | 1.38E-02 | 1.34E-02 | 1.46E-02 | 1.46E-02 | 1.42E-02 | 1.50E-02 |
| Pyruvate metabolism | 1.07E-02 | 1.04E-02 | 1.06E-02 | 1.02E-02 | 1.05E-02 | 1.02E-02 | 1.06E-02 | 1.07E-02 | 1.04E-02 | 9.82E-03 | 1.00E-02 | 9.96E-03 | 9.63E-03 | 9.58E-03 | 9.65E-03 | 9.66E-03 | 9.69E-03 | 9.57E-03 | 1.05E-02 | 1.01E-02 | 9.72E-03 | 1.02E-02 | 1.05E-02 | 9.97E-03 |
| RIG-I-like receptor signaling pathway | 3.36E-05 | 2.17E-05 | 1.59E-05 | 1.48E-05 | 1.11E-05 | 1.99E-05 | 1.32E-05 | 1.71E-05 | 1.30E-05 | 5.05E-05 | 3.63E-05 | 1.72E-05 | 1.49E-05 | 1.18E-05 | 2.16E-05 | 2.14E-05 | 2.19E-05 | 2.23E-05 | 1.85E-05 | 2.38E-05 | 2.57E-05 | 2.30E-05 | 1.49E-05 | 4.64E-05 |
| RNA degradation | 3.78E-03 | 4.10E-03 | 4.47E-03 | 3.93E-03 | 5.02E-03 | 3.98E-03 | 4.24E-03 | 4.21E-03 | 4.28E-03 | 3.85E-03 | 3.80E-03 | 4.09E-03 | 3.55E-03 | 3.51E-03 | 3.53E-03 | 3.54E-03 | 3.58E-03 | 3.48E-03 | 4.25E-03 | 4.09E-03 | 4.37E-03 | 4.51E-03 | 4.64E-03 | 4.47E-03 |
| RNA polymerase | 1.07E-03 | 1.05E-03 | 1.23E-03 | 9.94E-04 | 1.37E-03 | 9.86E-04 | 1.07E-03 | 1.14E-03 | 1.08E-03 | 1.13E-03 | 1.17E-03 | 1.06E-03 | 9.27E-04 | 9.27E-04 | 9.32E-04 | 9.32E-04 | 9.43E-04 | 9.27E-04 | 1.26E-03 | 1.19E-03 | 1.38E-03 | 1.35E-03 | 1.32E-03 | 1.31E-03 |
| RNA transport | 6.69E-04 | 9.62E-04 | 1.02E-03 | 9.95E-04 | 1.01E-03 | 1.02E-03 | 9.78E-04 | 9.18E-04 | 1.02E-03 | 1.00E-03 | 1.13E-03 | 9.96E-04 | 1.06E-03 | 1.07E-03 | 1.08E-03 | 1.09E-03 | 1.08E-03 | 1.10E-03 | 9.37E-04 | 1.01E-03 | 1.36E-03 | 8.25E-04 | 1.16E-03 | 1.41E-03 |
| Regulation of actin cytoskeleton | 0.00E+00 | 0.00E+00 | 0.00E+00 | 1.46E-07 | 4.17E-08 | 3.29E-08 | 0.00E+00 | 9.96E-08 | 0.00E+00 | 0.00E+00 | 0.00E+00 | 0.00E+00 | 0.00E+00 | 0.00E+00 | 0.00E+00 | 3.60E-08 | 0.00E+00 | 0.00E+00 | 4.55E-08 | 0.00E+00 | 0.00E+00 | 5.15E-07 | 1.37E-06 | 1.83E-07 |
| Renal cell carcinoma | 2.62E-04 | 2.14E-04 | 2.31E-04 | 2.32E-04 | 2.57E-04 | 2.23E-04 | 2.26E-04 | 2.37E-04 | 2.14E-04 | 2.75E-04 | 2.44E-04 | 2.15E-04 | 2.62E-04 | 2.68E-04 | 2.47E-04 | 2.48E-04 | 2.48E-04 | 2.42E-04 | 2.57E-04 | 2.44E-04 | 2.01E-04 | 2.87E-04 | 2.76E-04 | 2.33E-04 |
| Renin-angiotensin system | 5.42E-05 | 3.33E-05 | 1.00E-05 | 6.00E-06 | 3.42E-06 | 5.07E-06 | 6.86E-06 | 1.03E-05 | 3.26E-06 | 4.81E-05 | 4.30E-05 | 6.01E-06 | 2.52E-06 | 1.44E-06 | 2.10E-06 | 1.26E-06 | 3.17E-06 | 5.27E-07 | 5.68E-05 | 2.00E-05 | 1.36E-05 | 1.63E-05 | 1.55E-05 | 8.90E-06 |
| Replication, recombination and repair proteins | 7.94E-03 | 6.81E-03 | 7.61E-03 | 8.55E-03 | 9.11E-03 | 8.30E-03 | 7.75E-03 | 8.93E-03 | 8.05E-03 | 1.11E-02 | 1.13E-02 | 7.69E-03 | 1.07E-02 | 1.10E-02 | 1.17E-02 | 1.20E-02 | 1.11E-02 | 1.24E-02 | 7.36E-03 | 8.76E-03 | 8.84E-03 | 7.58E-03 | 8.26E-03 | 8.75E-03 |
| Restriction enzyme | 7.45E-04 | 1.16E-03 | 1.12E-03 | 1.06E-03 | 1.29E-03 | 1.06E-03 | 1.20E-03 | 1.12E-03 | 1.20E-03 | 1.14E-03 | 1.14E-03 | 1.24E-03 | 7.73E-04 | 7.59E-04 | 7.76E-04 | 7.52E-04 | 8.16E-04 | 7.50E-04 | 1.42E-03 | 1.09E-03 | 1.71E-03 | 1.02E-03 | 1.68E-03 | 1.85E-03 |
| Retinol metabolism | 8.62E-04 | 7.76E-04 | 7.58E-04 | 6.47E-04 | 9.44E-04 | 6.52E-04 | 7.18E-04 | 7.62E-04 | 6.77E-04 | 7.59E-04 | 7.29E-04 | 6.93E-04 | 6.53E-04 | 6.52E-04 | 6.27E-04 | 6.03E-04 | 6.29E-04 | 6.18E-04 | 9.68E-04 | 7.55E-04 | 8.14E-04 | 6.98E-04 | 7.91E-04 | 8.01E-04 |
| Rheumatoid arthritis | 0.00E+00 | 0.00E+00 | 0.00E+00 | 0.00E+00 | 0.00E+00 | 0.00E+00 | 0.00E+00 | 0.00E+00 | 0.00E+00 | 0.00E+00 | 0.00E+00 | 0.00E+00 | 0.00E+00 | 0.00E+00 | 0.00E+00 | 0.00E+00 | 0.00E+00 | 0.00E+00 | 0.00E+00 | 0.00E+00 | 0.00E+00 | 0.00E+00 | 0.00E+00 | 0.00E+00 |
| Riboflavin metabolism | 2.67E-03 | 2.81E-03 | 2.80E-03 | 2.82E-03 | 3.00E-03 | 2.81E-03 | 2.73E-03 | 2.73E-03 | 2.78E-03 | 3.00E-03 | 2.87E-03 | 2.97E-03 | 2.93E-03 | 2.95E-03 | 2.95E-03 | 2.98E-03 | 2.94E-03 | 2.98E-03 | 2.88E-03 | 2.85E-03 | 2.98E-03 | 3.09E-03 | 2.90E-03 | 2.87E-03 |
| Ribosome | 1.41E-02 | 1.43E-02 | 1.60E-02 | 1.34E-02 | 1.70E-02 | 1.36E-02 | 1.48E-02 | 1.53E-02 | 1.48E-02 | 1.54E-02 | 1.56E-02 | 1.45E-02 | 1.26E-02 | 1.25E-02 | 1.27E-02 | 1.27E-02 | 1.28E-02 | 1.27E-02 | 1.77E-02 | 1.63E-02 | 1.85E-02 | 1.83E-02 | 1.83E-02 | 1.84E-02 |
| Ribosome Biogenesis | 1.11E-02 | 1.36E-02 | 1.29E-02 | 1.33E-02 | 1.21E-02 | 1.35E-02 | 1.34E-02 | 1.26E-02 | 1.32E-02 | 1.36E-02 | 1.37E-02 | 1.41E-02 | 1.27E-02 | 1.25E-02 | 1.28E-02 | 1.28E-02 | 1.28E-02 | 1.27E-02 | 1.33E-02 | 1.28E-02 | 1.39E-02 | 1.30E-02 | 1.35E-02 | 1.36E-02 |
| Ribosome biogenesis in eukaryotes | 4.87E-04 | 5.05E-04 | 4.89E-04 | 4.85E-04 | 4.33E-04 | 4.84E-04 | 4.85E-04 | 4.73E-04 | 4.70E-04 | 5.40E-04 | 5.32E-04 | 4.34E-04 | 4.98E-04 | 5.00E-04 | 4.85E-04 | 4.81E-04 | 4.91E-04 | 4.66E-04 | 5.97E-04 | 5.51E-04 | 6.08E-04 | 6.16E-04 | 6.02E-04 | 5.74E-04 |
| Salivary secretion | 1.04E-06 | 7.00E-08 | 5.52E-08 | 1.46E-07 | 2.09E-07 | 9.87E-08 | 4.76E-08 | 1.99E-07 | 1.10E-07 | 0.00E+00 | 2.85E-08 | 0.00E+00 | 0.00E+00 | 0.00E+00 | 5.25E-08 | 0.00E+00 | 0.00E+00 | 0.00E+00 | 2.27E-07 | 5.87E-07 | 1.83E-06 | 1.34E-06 | 1.34E-06 | 1.52E-06 |
| Secondary bile acid biosynthesis | 3.48E-05 | 1.55E-04 | 9.61E-05 | 1.38E-04 | 7.31E-05 | 1.56E-04 | 1.23E-04 | 7.80E-05 | 1.39E-04 | 8.56E-05 | 9.47E-05 | 2.77E-04 | 9.70E-05 | 9.23E-05 | 8.91E-05 | 9.73E-05 | 9.42E-05 | 7.87E-05 | 1.09E-04 | 7.63E-05 | 6.64E-05 | 6.33E-05 | 5.42E-05 | 5.20E-05 |
| Secretion system | 2.14E-02 | 2.72E-02 | 2.29E-02 | 2.71E-02 | 1.92E-02 | 2.72E-02 | 2.44E-02 | 2.11E-02 | 2.44E-02 | 2.28E-02 | 2.28E-02 | 3.02E-02 | 2.63E-02 | 2.62E-02 | 2.56E-02 | 2.59E-02 | 2.58E-02 | 2.52E-02 | 2.06E-02 | 2.07E-02 | 1.87E-02 | 1.82E-02 | 2.15E-02 | 1.78E-02 |
| Selenocompound metabolism | 3.29E-03 | 2.83E-03 | 3.02E-03 | 2.92E-03 | 3.13E-03 | 2.91E-03 | 3.01E-03 | 3.25E-03 | 2.97E-03 | 3.42E-03 | 3.45E-03 | 3.22E-03 | 3.17E-03 | 3.18E-03 | 3.36E-03 | 3.39E-03 | 3.30E-03 | 3.53E-03 | 3.23E-03 | 3.54E-03 | 4.05E-03 | 3.40E-03 | 3.51E-03 | 4.09E-03 |
| Sesquiterpenoid biosynthesis | 3.59E-07 | 1.17E-07 | 2.76E-07 | 0.00E+00 | 0.00E+00 | 0.00E+00 | 0.00E+00 | 0.00E+00 | 0.00E+00 | 0.00E+00 | 1.14E-07 | 5.15E-07 | 0.00E+00 | 0.00E+00 | 0.00E+00 | 0.00E+00 | 0.00E+00 | 0.00E+00 | 0.00E+00 | 0.00E+00 | 0.00E+00 | 0.00E+00 | 2.89E-07 | 0.00E+00 |
| Shigellosis | 8.14E-06 | 2.05E-05 | 8.47E-06 | 2.77E-05 | 8.59E-06 | 2.21E-05 | 2.05E-05 | 2.10E-05 | 1.52E-05 | 7.31E-05 | 7.11E-05 | 1.03E-04 | 6.02E-05 | 6.63E-05 | 6.82E-05 | 7.48E-05 | 6.39E-05 | 5.51E-05 | 5.36E-06 | 2.99E-05 | 1.66E-05 | 1.09E-05 | 8.74E-06 | 8.77E-06 |
| Signal transduction mechanisms | 4.83E-03 | 5.38E-03 | 5.51E-03 | 5.63E-03 | 5.78E-03 | 5.70E-03 | 5.39E-03 | 5.24E-03 | 5.57E-03 | 5.47E-03 | 5.54E-03 | 5.41E-03 | 5.93E-03 | 5.95E-03 | 6.05E-03 | 6.12E-03 | 6.01E-03 | 6.14E-03 | 4.65E-03 | 5.21E-03 | 5.04E-03 | 4.70E-03 | 5.68E-03 | 4.91E-03 |
| Small cell lung cancer | 2.59E-04 | 4.24E-05 | 3.27E-05 | 4.10E-05 | 5.19E-05 | 3.12E-05 | 9.54E-05 | 1.62E-04 | 4.68E-05 | 1.30E-05 | 1.17E-05 | 1.22E-05 | 2.96E-06 | 4.14E-06 | 4.20E-06 | 3.92E-06 | 6.02E-06 | 4.10E-06 | 3.49E-05 | 4.70E-05 | 2.08E-05 | 7.40E-05 | 6.87E-05 | 4.55E-05 |
| Sphingolipid metabolism | 3.32E-04 | 5.72E-04 | 6.59E-04 | 6.47E-04 | 7.44E-04 | 7.24E-04 | 8.32E-04 | 6.07E-04 | 1.13E-03 | 4.30E-04 | 4.59E-04 | 6.11E-04 | 3.54E-04 | 3.12E-04 | 3.72E-04 | 3.56E-04 | 3.79E-04 | 3.51E-04 | 5.49E-04 | 6.14E-04 | 6.35E-04 | 6.75E-04 | 5.75E-04 | 7.49E-04 |
| Spliceosome | 0.00E+00 | 1.63E-07 | 2.48E-07 | 0.00E+00 | 8.34E-08 | 0.00E+00 | 0.00E+00 | 0.00E+00 | 0.00E+00 | 0.00E+00 | 0.00E+00 | 5.72E-08 | 0.00E+00 | 0.00E+00 | 0.00E+00 | 0.00E+00 | 4.60E-08 | 0.00E+00 | 2.27E-07 | 0.00E+00 | 0.00E+00 | 0.00E+00 | 0.00E+00 | 1.83E-07 |
| Sporulation | 2.81E-04 | 3.89E-04 | 7.50E-04 | 3.42E-04 | 8.38E-04 | 3.36E-04 | 3.48E-04 | 4.02E-04 | 3.74E-04 | 1.24E-04 | 4.08E-04 | 4.85E-04 | 9.38E-05 | 7.44E-05 | 9.02E-05 | 7.90E-05 | 1.47E-04 | 5.88E-05 | 4.44E-04 | 5.57E-04 | 8.44E-04 | 5.39E-04 | 4.21E-04 | 5.56E-04 |
| Staphylococcus aureus infection | 6.06E-05 | 5.70E-05 | 4.73E-05 | 6.53E-05 | 4.90E-05 | 7.31E-05 | 5.95E-05 | 4.97E-05 | 6.46E-05 | 1.64E-04 | 2.75E-04 | 5.14E-05 | 6.63E-05 | 7.25E-05 | 4.26E-05 | 3.98E-05 | 5.01E-05 | 2.55E-05 | 3.27E-05 | 5.81E-05 | 1.92E-04 | 3.27E-05 | 2.53E-05 | 2.72E-05 |
| Starch and sucrose metabolism | 4.75E-03 | 4.78E-03 | 5.92E-03 | 5.65E-03 | 7.22E-03 | 5.80E-03 | 5.54E-03 | 5.62E-03 | 6.43E-03 | 5.99E-03 | 6.26E-03 | 5.04E-03 | 5.88E-03 | 5.80E-03 | 6.22E-03 | 6.22E-03 | 6.12E-03 | 6.45E-03 | 5.36E-03 | 6.18E-03 | 6.10E-03 | 6.53E-03 | 5.52E-03 | 5.95E-03 |
| Steroid biosynthesis | 6.81E-05 | 5.63E-05 | 1.46E-04 | 5.25E-05 | 3.07E-04 | 5.88E-05 | 7.34E-05 | 9.40E-05 | 9.58E-05 | 2.32E-05 | 1.80E-05 | 3.00E-05 | 1.10E-05 | 9.30E-06 | 1.07E-05 | 7.88E-06 | 1.50E-05 | 8.91E-06 | 8.41E-05 | 1.00E-04 | 1.71E-04 | 8.82E-05 | 1.40E-04 | 1.34E-04 |
| Steroid hormone biosynthesis | 1.56E-04 | 2.20E-04 | 3.12E-04 | 2.32E-04 | 2.84E-04 | 2.56E-04 | 3.26E-04 | 2.56E-04 | 2.73E-04 | 1.27E-04 | 1.21E-04 | 1.31E-04 | 1.29E-04 | 1.15E-04 | 1.15E-04 | 1.13E-04 | 1.29E-04 | 7.03E-05 | 2.11E-04 | 2.05E-04 | 1.29E-04 | 1.70E-04 | 2.19E-04 | 1.62E-04 |
| Stilbenoid, diarylheptanoid and gingerol biosynthesis | 2.42E-04 | 1.33E-04 | 3.42E-04 | 7.45E-05 | 3.53E-04 | 6.99E-05 | 1.65E-04 | 2.39E-04 | 1.06E-04 | 4.87E-05 | 3.95E-05 | 1.30E-04 | 1.59E-05 | 1.46E-05 | 1.73E-05 | 1.18E-05 | 2.14E-05 | 1.02E-05 | 1.76E-04 | 2.03E-04 | 1.20E-04 | 1.59E-04 | 2.13E-04 | 1.32E-04 |
| Streptomycin biosynthesis | 2.23E-03 | 1.64E-03 | 2.14E-03 | 1.67E-03 | 2.89E-03 | 1.64E-03 | 2.01E-03 | 2.25E-03 | 2.12E-03 | 1.73E-03 | 1.69E-03 | 1.56E-03 | 1.61E-03 | 1.65E-03 | 1.61E-03 | 1.57E-03 | 1.61E-03 | 1.67E-03 | 2.27E-03 | 2.36E-03 | 2.72E-03 | 2.69E-03 | 2.57E-03 | 2.80E-03 |
| Styrene degradation | 1.05E-03 | 7.37E-04 | 6.94E-04 | 6.12E-04 | 5.39E-04 | 5.78E-04 | 6.92E-04 | 7.54E-04 | 5.85E-04 | 4.48E-04 | 4.31E-04 | 4.48E-04 | 6.12E-04 | 6.45E-04 | 4.74E-04 | 4.29E-04 | 5.04E-04 | 3.80E-04 | 8.00E-04 | 7.57E-04 | 5.61E-04 | 7.51E-04 | 7.01E-04 | 6.62E-04 |
| Sulfur metabolism | 3.09E-03 | 3.07E-03 | 3.11E-03 | 3.08E-03 | 3.26E-03 | 3.07E-03 | 3.07E-03 | 3.06E-03 | 3.20E-03 | 3.12E-03 | 3.05E-03 | 3.14E-03 | 3.04E-03 | 3.03E-03 | 3.05E-03 | 3.04E-03 | 3.05E-03 | 3.06E-03 | 3.09E-03 | 3.18E-03 | 3.65E-03 | 2.95E-03 | 3.34E-03 | 3.53E-03 |
| Sulfur relay system | 3.57E-03 | 4.06E-03 | 4.15E-03 | 4.04E-03 | 4.01E-03 | 4.11E-03 | 3.96E-03 | 3.87E-03 | 3.87E-03 | 3.99E-03 | 3.99E-03 | 3.97E-03 | 4.25E-03 | 4.27E-03 | 4.25E-03 | 4.28E-03 | 4.24E-03 | 4.28E-03 | 4.05E-03 | 3.97E-03 | 3.99E-03 | 3.61E-03 | 4.42E-03 | 3.79E-03 |
| Synthesis and degradation of ketone bodies | 1.69E-03 | 1.10E-03 | 9.05E-04 | 7.52E-04 | 5.31E-04 | 7.35E-04 | 9.71E-04 | 1.06E-03 | 7.42E-04 | 8.21E-04 | 8.11E-04 | 6.70E-04 | 6.74E-04 | 6.92E-04 | 5.59E-04 | 5.11E-04 | 5.91E-04 | 4.59E-04 | 1.32E-03 | 9.79E-04 | 7.56E-04 | 9.23E-04 | 1.02E-03 | 8.98E-04 |
| Systemic lupus erythematosus | 1.80E-05 | 6.58E-06 | 3.01E-06 | 1.05E-05 | 3.59E-06 | 7.01E-06 | 6.67E-06 | 1.48E-05 | 5.10E-06 | 1.52E-05 | 1.30E-05 | 5.15E-07 | 3.68E-05 | 4.27E-05 | 2.24E-05 | 1.96E-05 | 2.26E-05 | 1.58E-05 | 2.05E-06 | 1.14E-05 | 3.49E-06 | 4.74E-06 | 2.82E-06 | 3.59E-06 |
| TGF-beta signaling pathway | 0.00E+00 | 0.00E+00 | 0.00E+00 | 0.00E+00 | 0.00E+00 | 0.00E+00 | 0.00E+00 | 0.00E+00 | 0.00E+00 | 0.00E+00 | 0.00E+00 | 0.00E+00 | 0.00E+00 | 0.00E+00 | 0.00E+00 | 0.00E+00 | 0.00E+00 | 0.00E+00 | 0.00E+00 | 0.00E+00 | 0.00E+00 | 0.00E+00 | 0.00E+00 | 0.00E+00 |
| Taurine and hypotaurine metabolism | 1.35E-03 | 1.40E-03 | 1.31E-03 | 1.42E-03 | 1.25E-03 | 1.40E-03 | 1.45E-03 | 1.36E-03 | 1.41E-03 | 1.15E-03 | 1.19E-03 | 1.45E-03 | 1.19E-03 | 1.18E-03 | 1.16E-03 | 1.15E-03 | 1.18E-03 | 1.13E-03 | 1.29E-03 | 1.23E-03 | 1.34E-03 | 1.35E-03 | 1.31E-03 | 1.40E-03 |
| Terpenoid backbone biosynthesis | 4.22E-03 | 3.86E-03 | 4.51E-03 | 3.59E-03 | 4.69E-03 | 3.58E-03 | 4.00E-03 | 4.25E-03 | 4.00E-03 | 3.87E-03 | 3.85E-03 | 3.61E-03 | 3.24E-03 | 3.23E-03 | 3.18E-03 | 3.15E-03 | 3.24E-03 | 3.07E-03 | 4.90E-03 | 4.53E-03 | 5.04E-03 | 5.33E-03 | 4.88E-03 | 4.85E-03 |
| Tetracycline biosynthesis | 1.27E-03 | 1.30E-03 | 1.37E-03 | 1.30E-03 | 1.49E-03 | 1.30E-03 | 1.33E-03 | 1.36E-03 | 1.33E-03 | 1.18E-03 | 1.31E-03 | 1.27E-03 | 1.30E-03 | 1.31E-03 | 1.25E-03 | 1.26E-03 | 1.27E-03 | 1.23E-03 | 1.39E-03 | 1.26E-03 | 1.18E-03 | 1.02E-03 | 1.48E-03 | 1.31E-03 |
| Thiamine metabolism | 3.11E-03 | 3.25E-03 | 3.60E-03 | 3.31E-03 | 3.80E-03 | 3.27E-03 | 3.34E-03 | 3.42E-03 | 3.52E-03 | 3.49E-03 | 3.58E-03 | 3.62E-03 | 3.12E-03 | 3.09E-03 | 3.21E-03 | 3.23E-03 | 3.22E-03 | 3.27E-03 | 3.84E-03 | 3.72E-03 | 4.35E-03 | 3.81E-03 | 3.93E-03 | 4.28E-03 |
| Tight junction | 0.00E+00 | 0.00E+00 | 0.00E+00 | 1.46E-07 | 4.17E-08 | 3.29E-08 | 0.00E+00 | 9.96E-08 | 0.00E+00 | 0.00E+00 | 0.00E+00 | 0.00E+00 | 0.00E+00 | 0.00E+00 | 0.00E+00 | 3.60E-08 | 0.00E+00 | 0.00E+00 | 4.55E-08 | 0.00E+00 | 0.00E+00 | 5.15E-07 | 1.37E-06 | 1.83E-07 |
| Toluene degradation | 1.93E-03 | 2.00E-03 | 1.79E-03 | 1.94E-03 | 1.60E-03 | 1.93E-03 | 1.83E-03 | 1.82E-03 | 1.83E-03 | 2.20E-03 | 2.02E-03 | 1.90E-03 | 2.08E-03 | 2.07E-03 | 2.10E-03 | 2.11E-03 | 2.08E-03 | 2.14E-03 | 2.15E-03 | 2.03E-03 | 1.99E-03 | 2.01E-03 | 1.88E-03 | 1.99E-03 |
| Toxoplasmosis | 2.58E-04 | 4.24E-05 | 3.26E-05 | 4.02E-05 | 5.04E-05 | 2.93E-05 | 9.31E-05 | 1.59E-04 | 4.61E-05 | 1.30E-05 | 1.16E-05 | 1.22E-05 | 2.96E-06 | 4.02E-06 | 4.15E-06 | 3.89E-06 | 6.02E-06 | 4.10E-06 | 3.50E-05 | 4.73E-05 | 2.06E-05 | 7.40E-05 | 6.79E-05 | 4.50E-05 |
| Transcription factors | 1.74E-02 | 1.74E-02 | 1.62E-02 | 1.98E-02 | 1.41E-02 | 1.97E-02 | 1.78E-02 | 1.72E-02 | 1.81E-02 | 2.00E-02 | 2.06E-02 | 1.81E-02 | 2.18E-02 | 2.17E-02 | 2.26E-02 | 2.26E-02 | 2.21E-02 | 2.36E-02 | 1.55E-02 | 1.76E-02 | 1.54E-02 | 1.66E-02 | 1.42E-02 | 1.49E-02 |
| Transcription machinery | 6.23E-03 | 6.05E-03 | 6.59E-03 | 5.62E-03 | 6.61E-03 | 5.62E-03 | 6.01E-03 | 6.12E-03 | 6.05E-03 | 5.54E-03 | 5.56E-03 | 5.95E-03 | 5.35E-03 | 5.38E-03 | 5.16E-03 | 5.10E-03 | 5.25E-03 | 4.99E-03 | 7.12E-03 | 6.96E-03 | 7.87E-03 | 6.90E-03 | 7.60E-03 | 8.37E-03 |
| Transcription related proteins | 2.07E-04 | 1.94E-04 | 1.81E-04 | 2.96E-04 | 1.24E-04 | 2.96E-04 | 2.15E-04 | 2.32E-04 | 2.33E-04 | 4.08E-04 | 4.21E-04 | 1.90E-04 | 4.75E-04 | 4.78E-04 | 5.32E-04 | 5.23E-04 | 4.84E-04 | 6.25E-04 | 1.42E-04 | 2.59E-04 | 1.75E-04 | 1.69E-04 | 1.38E-04 | 1.60E-04 |
| Translation factors | 3.39E-03 | 3.62E-03 | 3.96E-03 | 3.53E-03 | 4.15E-03 | 3.57E-03 | 3.79E-03 | 3.81E-03 | 3.82E-03 | 3.68E-03 | 3.77E-03 | 3.77E-03 | 3.32E-03 | 3.30E-03 | 3.37E-03 | 3.39E-03 | 3.39E-03 | 3.38E-03 | 4.13E-03 | 3.98E-03 | 4.60E-03 | 4.16E-03 | 4.40E-03 | 4.56E-03 |
| Translation proteins | 6.94E-03 | 8.07E-03 | 8.33E-03 | 7.98E-03 | 8.23E-03 | 8.05E-03 | 8.02E-03 | 7.84E-03 | 8.05E-03 | 8.27E-03 | 8.44E-03 | 8.30E-03 | 7.99E-03 | 7.96E-03 | 8.09E-03 | 8.12E-03 | 8.07E-03 | 8.15E-03 | 8.06E-03 | 7.89E-03 | 8.54E-03 | 7.37E-03 | 8.41E-03 | 8.65E-03 |
| Transporters | 6.35E-02 | 5.38E-02 | 5.55E-02 | 5.94E-02 | 5.61E-02 | 5.82E-02 | 5.49E-02 | 5.80E-02 | 5.54E-02 | 6.09E-02 | 6.24E-02 | 5.53E-02 | 6.46E-02 | 6.46E-02 | 6.55E-02 | 6.56E-02 | 6.49E-02 | 6.66E-02 | 5.27E-02 | 5.85E-02 | 5.42E-02 | 6.27E-02 | 4.65E-02 | 4.73E-02 |
| Tropane, piperidine and pyridine alkaloid biosynthesis | 1.31E-03 | 1.12E-03 | 1.13E-03 | 1.11E-03 | 1.08E-03 | 1.11E-03 | 1.11E-03 | 1.23E-03 | 1.10E-03 | 1.31E-03 | 1.26E-03 | 1.02E-03 | 1.42E-03 | 1.46E-03 | 1.39E-03 | 1.38E-03 | 1.36E-03 | 1.41E-03 | 1.28E-03 | 1.28E-03 | 1.26E-03 | 1.28E-03 | 1.18E-03 | 1.32E-03 |
| Tryptophan metabolism | 6.61E-03 | 5.48E-03 | 4.83E-03 | 4.65E-03 | 3.68E-03 | 4.59E-03 | 5.28E-03 | 5.46E-03 | 4.45E-03 | 4.53E-03 | 4.30E-03 | 4.97E-03 | 4.59E-03 | 4.65E-03 | 4.25E-03 | 4.15E-03 | 4.33E-03 | 3.97E-03 | 6.04E-03 | 5.36E-03 | 4.57E-03 | 5.44E-03 | 5.29E-03 | 4.96E-03 |

| | | | | | | | | | | | | | | | | | | | | | | | | |
|---|---|---|---|---|---|---|---|---|---|---|---|---|---|---|---|---|---|---|---|---|---|---|---|---|
| Tuberculosis | 1.34E-03 | 1.35E-03 | 1.69E-03 | 1.23E-03 | 2.32E-03 | 1.25E-03 | 1.37E-03 | 1.46E-03 | 1.42E-03 | 1.01E-03 | 1.02E-03 | 1.29E-03 | 8.54E-04 | 8.27E-04 | 8.45E-04 | 8.36E-04 | 8.78E-04 | 8.31E-04 | 1.28E-03 | 1.23E-03 | 1.34E-03 | 1.32E-03 | 1.43E-03 | 1.25E-03 |
| Two-component system | 2.49E-02 | 2.77E-02 | 2.51E-02 | 2.86E-02 | 2.35E-02 | 2.87E-02 | 2.62E-02 | 2.42E-02 | 2.70E-02 | 2.52E-02 | 2.50E-02 | 2.63E-02 | 2.91E-02 | 2.92E-02 | 2.86E-02 | 2.86E-02 | 2.86E-02 | 2.82E-02 | 2.16E-02 | 2.36E-02 | 2.33E-02 | 2.07E-02 | 2.20E-02 | 2.21E-02 |
| Type I diabetes mellitus | 4.01E-04 | 5.03E-04 | 5.43E-04 | 4.85E-04 | 5.58E-04 | 4.91E-04 | 4.82E-04 | 4.37E-04 | 5.12E-04 | 5.03E-04 | 4.84E-04 | 6.22E-04 | 4.20E-04 | 4.15E-04 | 4.34E-04 | 4.45E-04 | 4.32E-04 | 4.34E-04 | 4.41E-04 | 4.25E-04 | 4.53E-04 | 4.49E-04 | 4.45E-04 | 4.26E-04 |
| Type II diabetes mellitus | 4.08E-04 | 4.41E-04 | 5.18E-04 | 4.61E-04 | 6.83E-04 | 4.71E-04 | 4.65E-04 | 4.51E-04 | 4.90E-04 | 4.06E-04 | 4.18E-04 | 4.84E-04 | 4.46E-04 | 4.47E-04 | 4.46E-04 | 4.55E-04 | 4.46E-04 | 4.50E-04 | 3.46E-04 | 3.93E-04 | 3.16E-04 | 4.00E-04 | 3.87E-04 | 3.21E-04 |
| Tyrosine metabolism | 4.53E-03 | 4.31E-03 | 4.05E-03 | 4.13E-03 | 4.10E-03 | 4.07E-03 | 4.17E-03 | 4.29E-03 | 4.07E-03 | 4.06E-03 | 4.04E-03 | 3.93E-03 | 4.45E-03 | 4.56E-03 | 4.20E-03 | 4.15E-03 | 4.24E-03 | 4.12E-03 | 4.33E-03 | 4.22E-03 | 3.83E-03 | 4.01E-03 | 3.78E-03 | 4.23E-03 |
| Ubiquinone and other terpenoid-quinone biosynthesis | 3.61E-03 | 4.41E-03 | 4.60E-03 | 4.40E-03 | 5.18E-03 | 4.50E-03 | 4.37E-03 | 4.18E-03 | 4.42E-03 | 4.24E-03 | 3.95E-03 | 4.54E-03 | 4.30E-03 | 4.24E-03 | 4.39E-03 | 4.45E-03 | 4.36E-03 | 4.48E-03 | 4.05E-03 | 4.01E-03 | 3.75E-03 | 4.02E-03 | 4.24E-03 | 3.81E-03 |
| Ubiquitin system | 1.60E-04 | 2.26E-04 | 2.73E-04 | 2.27E-04 | 2.59E-04 | 2.32E-04 | 2.21E-04 | 2.07E-04 | 2.12E-04 | 2.45E-04 | 2.33E-04 | 2.59E-04 | 2.29E-04 | 2.29E-04 | 2.30E-04 | 2.30E-04 | 2.30E-04 | 2.32E-04 | 2.81E-04 | 2.71E-04 | 3.06E-04 | 1.75E-04 | 2.81E-04 | 2.67E-04 |
| VEGF signaling pathway | 1.16E-06 | 9.33E-08 | 1.38E-07 | 1.02E-06 | 3.96E-06 | 1.97E-07 | 5.24E-07 | 8.07E-06 | 2.93E-07 | 3.35E-08 | 8.54E-08 | 0.00E+00 | 0.00E+00 | 6.00E-08 | 5.25E-08 | 3.60E-08 | 0.00E+00 | 0.00E+00 | 1.36E-07 | 8.80E-07 | 0.00E+00 | 2.06E-07 | 6.14E-07 | 0.00E+00 |
| Valine, leucine and isoleucine biosynthesis | 6.28E-03 | 5.78E-03 | 6.19E-03 | 5.66E-03 | 6.59E-03 | 5.63E-03 | 6.04E-03 | 6.39E-03 | 5.92E-03 | 6.03E-03 | 5.98E-03 | 5.55E-03 | 5.45E-03 | 5.48E-03 | 5.48E-03 | 5.49E-03 | 5.52E-03 | 5.49E-03 | 6.79E-03 | 6.32E-03 | 6.38E-03 | 6.95E-03 | 6.50E-03 | 6.42E-03 |
| Valine, leucine and isoleucine degradation | 9.37E-03 | 7.54E-03 | 6.76E-03 | 6.03E-03 | 5.04E-03 | 5.91E-03 | 7.18E-03 | 7.39E-03 | 6.10E-03 | 5.93E-03 | 5.55E-03 | 6.36E-03 | 5.39E-03 | 5.42E-03 | 4.86E-03 | 4.62E-03 | 5.02E-03 | 4.36E-03 | 8.49E-03 | 7.11E-03 | 6.23E-03 | 7.38E-03 | 7.35E-03 | 6.89E-03 |
| Various types of N-glycan biosynthesis | 4.57E-07 | 6.46E-06 | 2.57E-06 | 8.78E-07 | 3.34E-07 | 9.54E-07 | 5.72E-07 | 8.47E-07 | 3.34E-06 | 6.71E-07 | 4.56E-07 | 1.04E-05 | 1.26E-07 | 1.80E-07 | 5.25E-08 | 7.20E-08 | 1.84E-07 | 0.00E+00 | 4.09E-07 | 1.17E-06 | 2.28E-05 | 1.34E-06 | 2.31E-06 | 2.50E-05 |
| Vascular smooth muscle contraction | 0.00E+00 | 2.33E-08 | 2.76E-08 | 0.00E+00 | 0.00E+00 | 6.58E-08 | 0.00E+00 | 0.00E+00 | 7.34E-08 | 0.00E+00 | 0.00E+00 | 0.00E+00 | 0.00E+00 | 0.00E+00 | 0.00E+00 | 0.00E+00 | 0.00E+00 | 0.00E+00 | 9.09E-08 | 2.93E-07 | 0.00E+00 | 0.00E+00 | 1.45E-07 | 0.00E+00 |
| Vasopressin-regulated water reabsorption | 2.77E-07 | 1.38E-06 | 2.04E-06 | 5.85E-07 | 8.34E-07 | 7.24E-07 | 8.10E-07 | 1.15E-06 | 6.97E-07 | 1.68E-07 | 0.00E+00 | 7.44E-07 | 6.30E-08 | 0.00E+00 | 3.15E-07 | 1.08E-07 | 4.60E-08 | 1.17E-07 | 2.14E-06 | 2.05E-06 | 3.32E-06 | 1.75E-06 | 3.25E-06 | 2.25E-06 |
| Vibrio cholerae infection | 2.23E-05 | 2.84E-04 | 2.11E-04 | 2.24E-04 | 1.12E-04 | 2.53E-04 | 2.08E-04 | 8.86E-05 | 2.29E-04 | 5.21E-05 | 5.05E-05 | 4.20E-04 | 5.05E-05 | 3.28E-05 | 4.15E-05 | 4.26E-05 | 5.36E-05 | 1.83E-05 | 8.48E-05 | 5.46E-05 | 5.43E-05 | 7.49E-05 | 7.80E-05 | 4.76E-05 |
| Vibrio cholerae pathogenic cycle | 1.31E-03 | 4.45E-03 | 3.60E-03 | 4.19E-03 | 2.74E-03 | 4.45E-03 | 3.89E-03 | 2.29E-03 | 3.97E-03 | 1.80E-03 | 1.82E-03 | 5.35E-03 | 2.10E-03 | 1.83E-03 | 2.01E-03 | 2.01E-03 | 2.17E-03 | 1.75E-03 | 2.04E-03 | 1.72E-03 | 1.64E-03 | 1.64E-03 | 2.05E-03 | 1.53E-03 |
| Viral myocarditis | 2.58E-04 | 4.24E-05 | 3.26E-05 | 4.04E-05 | 5.05E-05 | 2.92E-05 | 9.31E-05 | 1.59E-04 | 4.60E-05 | 1.30E-05 | 1.16E-05 | 1.22E-05 | 2.96E-06 | 4.02E-06 | 4.15E-06 | 3.92E-06 | 6.02E-06 | 4.10E-06 | 3.49E-05 | 4.70E-05 | 2.06E-05 | 7.45E-05 | 6.91E-05 | 4.51E-05 |
| Vitamin B6 metabolism | 1.71E-03 | 1.75E-03 | 1.76E-03 | 1.85E-03 | 1.80E-03 | 1.87E-03 | 1.85E-03 | 1.80E-03 | 1.91E-03 | 2.02E-03 | 1.95E-03 | 1.86E-03 | 1.95E-03 | 1.95E-03 | 1.99E-03 | 2.01E-03 | 1.97E-03 | 2.01E-03 | 2.09E-03 | 2.04E-03 | 2.11E-03 | 2.11E-03 | 2.02E-03 | 2.06E-03 |
| Wnt signaling pathway | 1.31E-07 | 3.27E-07 | 7.73E-07 | 2.93E-07 | 1.25E-07 | 1.97E-07 | 7.62E-07 | 3.99E-07 | 2.20E-07 | 3.35E-08 | 5.69E-08 | 6.87E-07 | 2.52E-07 | 1.20E-07 | 4.73E-07 | 2.16E-07 | 2.30E-07 | 0.00E+00 | 7.27E-07 | 8.80E-07 | 0.00E+00 | 6.19E-07 | 3.97E-07 | 3.05E-07 |
| Xylene degradation | 4.64E-04 | 4.41E-04 | 5.10E-04 | 5.21E-04 | 5.64E-04 | 5.41E-04 | 5.01E-04 | 4.94E-04 | 5.19E-04 | 5.73E-04 | 6.31E-04 | 5.26E-04 | 5.89E-04 | 5.82E-04 | 6.64E-04 | 6.59E-04 | 6.23E-04 | 7.71E-04 | 4.02E-04 | 5.37E-04 | 4.92E-04 | 3.57E-04 | 3.15E-04 | 3.89E-04 |
| Zeatin biosynthesis | 2.60E-04 | 2.64E-04 | 2.95E-04 | 2.53E-04 | 3.18E-04 | 2.53E-04 | 2.74E-04 | 2.83E-04 | 2.98E-04 | 2.81E-04 | 2.88E-04 | 2.67E-04 | 2.33E-04 | 2.32E-04 | 2.33E-04 | 2.33E-04 | 2.36E-04 | 2.32E-04 | 3.29E-04 | 3.05E-04 | 3.48E-04 | 3.43E-04 | 3.43E-04 | 3.47E-04 |
| alpha-Linolenic acid metabolism | 4.32E-04 | 6.12E-04 | 4.78E-04 | 5.80E-04 | 4.14E-04 | 5.91E-04 | 5.27E-04 | 4.28E-04 | 5.06E-04 | 5.50E-04 | 5.38E-04 | 8.33E-04 | 5.84E-04 | 5.71E-04 | 6.10E-04 | 6.20E-04 | 6.05E-04 | 6.23E-04 | 6.06E-04 | 5.58E-04 | 4.80E-04 | 6.99E-04 | 4.75E-04 | 4.95E-04 |
| beta-Alanine metabolism | 5.29E-03 | 4.52E-03 | 4.23E-03 | 3.95E-03 | 3.35E-03 | 3.88E-03 | 4.46E-03 | 4.59E-03 | 3.81E-03 | 4.09E-03 | 3.96E-03 | 4.39E-03 | 3.98E-03 | 4.00E-03 | 3.89E-03 | 3.81E-03 | 3.89E-03 | 3.82E-03 | 4.86E-03 | 4.50E-03 | 3.71E-03 | 4.27E-03 | 4.34E-03 | 3.70E-03 |
| beta-Lactam resistance | 3.11E-04 | 2.52E-04 | 2.13E-04 | 2.02E-04 | 2.53E-04 | 2.03E-04 | 1.99E-04 | 1.94E-04 | 1.92E-04 | 2.53E-04 | 2.63E-04 | 2.30E-04 | 2.22E-04 | 2.23E-04 | 2.22E-04 | 2.20E-04 | 2.20E-04 | 2.22E-04 | 3.06E-04 | 2.43E-04 | 1.98E-04 | 2.64E-04 | 2.14E-04 | 2.08E-04 |
| mRNA surveillance pathway | 6.53E-07 | 3.50E-07 | 2.48E-07 | 5.85E-07 | 5.42E-07 | 5.27E-07 | 1.91E-07 | 2.99E-07 | 1.10E-07 | 1.34E-07 | 2.85E-08 | 2.86E-07 | 6.30E-08 | 1.80E-07 | 1.58E-07 | 7.20E-08 | 1.84E-07 | 2.34E-07 | 2.51E-05 | 1.17E-06 | 4.98E-07 | 3.09E-07 | 4.34E-07 | 1.83E-07 |
| mTOR signaling pathway | 0.00E+00 | 0.00E+00 | 0.00E+00 | 0.00E+00 | 0.00E+00 | 0.00E+00 | 0.00E+00 | 0.00E+00 | 0.00E+00 | 0.00E+00 | 0.00E+00 | 0.00E+00 | 0.00E+00 | 0.00E+00 | 0.00E+00 | 0.00E+00 | 0.00E+00 | 0.00E+00 | 0.00E+00 | 0.00E+00 | 0.00E+00 | 0.00E+00 | 0.00E+00 | 0.00E+00 |
| p53 signaling pathway | 2.60E-04 | 6.41E-05 | 4.11E-05 | 5.98E-05 | 5.99E-05 | 5.19E-05 | 1.21E-04 | 1.75E-04 | 6.81E-05 | 1.43E-05 | 1.30E-05 | 1.01E-04 | 5.36E-06 | 5.58E-06 | 6.30E-06 | 5.94E-06 | 8.09E-06 | 5.39E-06 | 4.04E-05 | 5.02E-05 | 2.41E-05 | 7.66E-05 | 7.25E-05 | 5.04E-05 |

**Supplementary Table S8.** NSTI mean score for all the sequenced groups.

| | intestine | | | Hepatopancreas | | | Pond sediment | |
|---|---|---|---|---|---|---|---|---|
| | wt | healthy cultured | diseased cultured | wt | healthy cultured | diseased cultured | Healthy | Diseased |
| NSTI score | 0.081 ± 0.035 | 0.112 ± 0.072 | 0.1 ± 0.007 | 0.046 ± 0.005 | 0.052 ± 0.003 | 0.05 ± 0.003 | 0.142 ± 0.027 | 0.132 ± 0.029 |

| | intestine | Hepatopancreas | Pond sediment |
|---|---|---|---|
| NSTI score | 0.098 ± 0.043 | 0.0492 ± 0.004 | 0.137 ± 0.026 |

**Supplementary Table S9**. Linear Discriminant Analysis of enriched taxonomies in wt and cultured hepatopancreas samples using LEfSe.

## Cultured samples

| Phyla | LDA |
|---|---|
| p_Proteobacteria | 4.742 |
| p_Gemmatimonadetes | 3.680 |
| p_Spirochaetes | 2.621 |

| Class | LDA |
|---|---|
| c_Gammaproteobacteria | 5.068 |
| c_TK17 | 3.484 |
| c_Gemm_5 | 3.458 |
| c_Gemm_2 | 3.383 |
| c_iii1_8 | 2.959 |
| c_Rhodothermi | 2.741 |
| c_Leptospirae | 2.633 |

| Order | LDA |
|---|---|
| o_Enterobacteriales | 5.308 |
| o_Stramenopiles | 3.353 |
| o_Legionellales | 2.927 |
| o_Thiohalorhabdales | 2.777 |
| o_Rhodothermales | 2.717 |
| o_Leptospirales | 2.714 |
| o_Desulfobacterales | 2.590 |
| o_DS_18 | 2.532 |

| Family | LDA |
|---|---|
| f_Enterobacteriaceae | 5.323 |
| f_Pseudomonadaceae | 4.767 |
| f_Coxiellaceae | 2.885 |
| f_Thiohalorhabdaceae | 2.734 |
| f_Rhodothermaceae | 2.707 |
| f_Desulfobulbaceae | 2.704 |
| f_Bacteroidaceae | 2.693 |
| f_Alcanivoracaceae | 2.686 |
| f_Psychromonadaceae | 2.570 |
| f_Christensenellaceae | 2.554 |
| f_Balneolaceae | 2.549 |
| f_Piscirickettsiaceae | 2.426 |

| Genus | LDA |
|---|---|
| g_Pseudomonas | 4.743 |
| g_Enterobacter | 4.080 |
| g_Trabulsiella | 3.891 |
| g_Escherichia | 3.820 |
| g_Stenotrophomonas | 3.425 |
| g_Citrobacter | 3.216 |
| g_Klebsiella | 3.214 |
| g_Bacteroides | 3.015 |
| g_Alcanivorax | 2.770 |
| g_Pantoea | 2.664 |
| g_Rummeliibacillus | 2.591 |
| g_Zobellella | 2.580 |
| g_Umboniibacter | 2.578 |
| g_KSA1 | 2.501 |

## Wild-type samples

| Phyla | LDA |
|---|---|
| p_Actinobacteria | 4.646 |
| p_Firmicutes | 4.597 |
| p_TM7 | 2.951 |

| Class | LDA |
|---|---|
| c_Actinobacteria | 4.596 |
| c_Bacilli | 4.406 |
| c_Clostridia | 4.168 |
| c_Alphaproteobacteria | 3.511 |
| c_TM7_3 | 2.910 |
| c_RB25 | 2.663 |
| c_Thermoleophilia | 2.455 |

| Order | LDA |
|---|---|
| o_Actinomycetales | 4.560 |
| o_Alteromonadales | 4.364 |
| o_Lactobacillales | 4.288 |
| o_Clostridiales | 4.189 |
| o_Bacillales | 3.725 |
| o_Aeromonadales | 3.660 |
| o_Streptophyta | 3.265 |
| o_Rhodospirillales | 2.998 |
| o_Rhizobiales | 2.861 |
| o_Caulobacterales | 2.857 |

| Family | LDA |
|---|---|
| f_Streptococcaceae | 4.205 |
| f_Shewanellaceae | 4.158 |
| f_Corynebacteriaceae | 3.798 |
| f_Micrococcaceae | 3.719 |
| f_Staphylococcaceae | 3.634 |
| f_Aeromonadaceae | 3.628 |
| f_Tissierellaceae | 3.400 |
| f_Carnobacteriaceae | 3.312 |
| f_Intrasporangiaceae | 3.270 |
| f_Endozoicimonaceae | 3.080 |
| f_Caulobacteraceae | 2.872 |
| f_Acidaminobacteraceae | 2.831 |
| f_Nocardioidaceae | 2.686 |
| f_Dermabacteraceae | 2.682 |
| f_Ferrimonadaceae | 2.474 |
| f_Rhizobiaceae | 2.459 |
| f_Exiguobacteraceae | 2.447 |
| f_Halomonadaceae | 2.435 |

| Genus | LDA |
|---|---|
| g_Streptococcus | 4.136 |
| g_Shewanella | 4.129 |
| g_Corynebacterium | 3.847 |
| g_Vibrio | 3.807 |
| g_Staphylococcus | 3.612 |
| g_Haemophilus | 3.609 |
| g_Rothia | 3.534 |
| g_Granulicatella | 3.296 |
| g_Actinobacillus | 3.017 |
| g_Brevundimonas | 2.933 |
| g_Kocuria | 2.905 |
| g_Fusibacter | 2.856 |
| g_Anaerococcus | 2.758 |
| g_Micrococcus | 2.727 |
| g_Agrobacterium | 2.702 |
| g_Paracoccus | 2.661 |
| g_Microbacterium | 2.655 |
| g_Dermabacter | 2.497 |
| g_Epulopiscium | 2.481 |
| g_Ferrimonas | 2.467 |
| g_Nocardioides | 2.439 |

**Supplementary Table S10.** Relative frequency of unique genera and species in sequenced wt and cultured samples.

| **genera_helathy_vs_wt_intestine** | | | Intestine | | | |
|---|---|---|---|---|---|---|
| Treatment | healthy cultured | | | wt | | |
| Description | 139-i | 68B-i | 7A-i | i 1 wt | i 2 wt | i 3 wt |
| g__Bacteroides | 0.00348028 | 0.00251889 | 0.00142395 | 0 | 0 | 0 |
| g__Brenneria | 0.00232019 | 0.00013257 | 0.0007767 | 0 | 0 | 0 |
| g__Bilophila | 0.00174014 | 0.00026515 | 0.0018123 | 0 | 0 | 0 |
| g__Rhodovulum | 0.00174014 | 0.00013257 | 0.00012945 | 0 | 0 | 0 |
| g__Robiginitalea | 0.00116009 | 0.00291661 | 0.00711974 | 0 | 0 | 0 |
| g__Coprococcus | 0.00116009 | 0.00039772 | 0.00142395 | 0 | 0 | 0 |
| g__KSA1 | 0.00116009 | 0.00159088 | 0.00090615 | 0 | 0 | 0 |
| g__Morganella | 0.00116009 | 0.00013257 | 0.00064725 | 0 | 0 | 0 |
| g__Microbulbifer | 0.00116009 | 0.00039772 | 0.0005178 | 0 | 0 | 0 |
| g__Blautia | 0.00116009 | 0.00079544 | 0.00038835 | 0 | 0 | 0 |
| g__Faecalibacterium | 0.00116009 | 0.00066287 | 0.00038835 | 0 | 0 | 0 |
| g__Bifidobacterium | 0.00058005 | 0.00013257 | 0.00064725 | 0 | 0 | 0 |
| g__Butyrivibrio | 0.00058005 | 0.00013257 | 0.00012945 | 0 | 0 | 0 |
| g__Plesiocystis | 0.00058005 | 0.00013257 | 0.00012945 | 0 | 0 | 0 |
| g__Tolumonas | 0.00058005 | 0.00013257 | 0.00012945 | 0 | 0 | 0 |
| g__Rubellimicrobium | 0 | 0 | 0 | 0.02668522 | 0.0011478 | 0.00037303 |
| g__Janthinobacterium | 0 | 0 | 0 | 0.01846465 | 0.00264877 | 0.00018651 |
| g__Blastococcus | 0 | 0 | 0 | 0.00739851 | 0.00044146 | 0.00027977 |
| g__Agrococcus | 0 | 0 | 0 | 0.00733527 | 0.00061805 | 0.00027977 |
| g__Lysobacter | 0 | 0 | 0 | 0.00505881 | 8.83E-05 | 9.33E-05 |
| g__Pleomorphomonas | 0 | 0 | 0 | 0.0048691 | 8.83E-05 | 9.33E-05 |
| g__Pseudomonas | 0 | 0 | 0 | 0.0046794 | 0.00185414 | 9.33E-05 |
| g__Brevundimonas | 0 | 0 | 0 | 0.00411028 | 0.00035317 | 9.33E-05 |
| g__Crenothrix | 0 | 0 | 0 | 0.00284558 | 8.83E-05 | 0.00018651 |
| g__Agrobacterium | 0 | 0 | 0 | 0.00259264 | 0.00026488 | 0.00018651 |
| g__Dietzia | 0 | 0 | 0 | 0.0023397 | 8.83E-05 | 0.00027977 |
| g__Microbispora | 0 | 0 | 0 | 0.00139117 | 0.00044146 | 9.33E-05 |
| g__Serinicoccus | 0 | 0 | 0 | 0.00094853 | 8.83E-05 | 0.00027977 |
| g__Ralstonia | 0 | 0 | 0 | 0.00088529 | 0.0081229 | 0.00018651 |
| g__Leptothrix | 0 | 0 | 0 | 0.00044265 | 0.00035317 | 9.33E-05 |
| g__Streptomyces | 0 | 0 | 0 | 0.00031618 | 0.00017658 | 0.00027977 |
| g__Novosphingobium | 0 | 0 | 0 | 0.00031618 | 8.83E-05 | 9.33E-05 |
| g__Jannaschia | 0 | 0 | 0 | 0.00012647 | 8.83E-05 | 0.00037303 |
| g__Shinella | 0 | 0 | 0 | 0.00012647 | 0.00035317 | 0.00018651 |
| g__Bacillus | 0 | 0 | 0 | 6.32E-05 | 8.83E-05 | 0.00018651 |
| g__Nitrobacteria | 0 | 0 | 0 | 6.32E-05 | 0.00070634 | 9.33E-05 |
| g__Acinetobacter | 0 | 0 | 0 | 0.00018971 | 8.83E-05 | 9.33E-05 |
| g__Variovorax | 0 | 0 | 0 | 6.32E-05 | 0.00264877 | 0.00018651 |

**Supplementary Table S10.** Relative frequency of unique genera and species in sequenced wt and cultured samples.

| **spp_helathy_vs_wt_intestine** | | intestine | | | | | |
|---|---|---|---|---|---|---|---|
| Treatment | Treatment | healthy cultured | | | wt | | |
| Description | Description | 139-i | 68B-i | 7A-i | i 1 wt | i 2 wt | i 3 wt |
| g__Agrococcus | s__jenensis | 0 | 0 | 0 | 0.00733527 | 0.00061805 | 0.00027977 |
| g__Blastococcus | s__aggregatus | 0 | 0 | 0 | 0.00739851 | 0.00044146 | 0.00027977 |
| g__Variovorax | s__paradoxus | 0 | 0 | 0 | 6.32E-05 | 0.00264877 | 0.00018651 |
| g__Crenothrix | s__polyspora | 0 | 0 | 0 | 0.00284558 | 8.83E-05 | 0.00018651 |
| g__Bacillus | s__flexus | 0 | 0 | 0 | 6.32E-05 | 8.83E-05 | 0.00018651 |
| g__Pseudomonas | s__stutzeri | 0 | 0 | 0 | 0.0046794 | 0.00185414 | 9.33E-05 |
| g__Nitrobacteria | s__hamadaniensis | 0 | 0 | 0 | 6.32E-05 | 0.00070634 | 9.33E-05 |
| g__Microbispora | s__rosea | 0 | 0 | 0 | 0.00139117 | 0.00044146 | 9.33E-05 |
| g__Brevundimonas | s__diminuta | 0 | 0 | 0 | 0.00411028 | 0.00035317 | 9.33E-05 |
| g__Acinetobacter | s__schindleri | 0 | 0 | 0 | 0.00018971 | 8.83E-05 | 9.33E-05 |
| g__Coprococcus | s__eutactus | 0.00116009 | 0.00039772 | 0.00142395 | 0 | 0 | 0 |
| g__Brenneria | s__alni | 0.00232019 | 0.00013257 | 0.0007767 | 0 | 0 | 0 |
| g__Morganella | s__morganii | 0.00116009 | 0.00013257 | 0.00064725 | 0 | 0 | 0 |
| g__Faecalibacterium | s__prausnitzii | 0.00116009 | 0.00066287 | 0.00038835 | 0 | 0 | 0 |
| g__Rhodovulum | s__imhoffii | 0.00174014 | 0.00013257 | 0.00012945 | 0 | 0 | 0 |

**Supplementary Table S10.** Relative frequency of unique genera and species in sequenced wt and cultured samples.

| **genera_healthy_vs_wt_hepatopancreas** | | | hepatopancreas | | | |
|---|---|---|---|---|---|---|
| Treatment | | healthy cultured | | | wt | |
| Description | 139-H | 68B-H | 7A-H | h 1 wt | h 2 wt | h 3 wt |
| g__Trabulsiella | 0.01621622 | 0.02481903 | 0.0098832 | 0 | 0 | 0 |
| g__Zobellella | 0.00108108 | 0.00025853 | 0.00044924 | 0 | 0 | 0 |
| g__Enterobacter | 0.00081081 | 0.00025853 | 0.00112309 | 0 | 0 | 0 |
| g__Bacteroides | 0.00081081 | 0.00025853 | 0.00022462 | 0 | 0 | 0 |
| g__Alcanivorax | 0.00054054 | 0.00051706 | 0.00044924 | 0 | 0 | 0 |
| g__KSA1 | 0.00054054 | 0.00077559 | 0.00022462 | 0 | 0 | 0 |
| g__Haemophilus | 0 | 0 | 0 | 0.01244615 | 0.01186441 | 0.00042526 |
| g__Granulicatella | 0 | 0 | 0 | 0.00430828 | 0.00937188 | 0.00042526 |
| g__Rothia | 0 | 0 | 0 | 0.00347056 | 0.01625125 | 0.00063789 |
| g__Kocuria | 0 | 0 | 0 | 0.00191479 | 0.00139581 | 0.00106315 |
| g__Staphylococcus | 0 | 0 | 0 | 0.00155577 | 0.00179462 | 0.00021263 |
| g__Vibrio | 0 | 0 | 0 | 0.00143609 | 0.00089731 | 0.0323198 |
| g__Anaerococcus | 0 | 0 | 0 | 0.00083772 | 0.00289133 | 0.00021263 |
| g__Micrococcus | 0 | 0 | 0 | 0.00071805 | 0.00159521 | 0.00042526 |
| g__Microbacterium | 0 | 0 | 0 | 0.00071805 | 0.00109671 | 0.00021263 |
| g__Rothia | 0 | 0 | 0 | 0.00059837 | 0.00119641 | 0.00063789 |
| g__Dermabacter | 0 | 0 | 0 | 0.00059837 | 0.0004985 | 0.00021263 |
| g__Paracoccus | 0 | 0 | 0 | 0.00059837 | 9.97E-05 | 0.00021263 |
| g__Rothia | 0 | 0 | 0 | 0.00035902 | 0.0002991 | 0.00063789 |
| g__Brevundimonas | 0 | 0 | 0 | 0.00035902 | 0.0001994 | 0.00021263 |
| g__Nocardioides | 0 | 0 | 0 | 0.00035902 | 9.97E-05 | 0.00021263 |
| g__Fusibacter | 0 | 0 | 0 | 0.00023935 | 0.0003988 | 0.00297682 |
| g__Marinomonas | 0 | 0 | 0 | 0.00011967 | 0.0003988 | 0.0021263 |
| g__Epulopiscium | 0 | 0 | 0 | 0.00011967 | 0.0003988 | 0.00106315 |
| g__Agrobacterium | 0 | 0 | 0 | 0.00011967 | 0.0004985 | 0.00021263 |
| g__Staphylococcus | 0 | 0 | 0 | 0.00813787 | 0.00418744 | 0.00085052 |

**Supplementary Table S10.** Relative frequency of unique genera and species in sequenced wt and cultured samples.

| **spp_helathy_vs_wt_hepatopancreas** | | | hepatopancreas | | | |
|---|---|---|---|---|---|---|
| Treatment | | healthy cultured | | | diseased cultured | |
| Description | 139-H | 68B-H | 7A-H | h 1 wt | h 2 wt | h 3 wt |
| Zobellella taiwanensis | 0.00108108 | 0.00025853 | 0.00044924 | 0 | 0 | 0 |
| Enterobacter cowanii | 0.00081081 | 0.00025853 | 0.00112309 | 0 | 0 | 0 |
| Haemophilus parainfluenzae | 0 | 0 | 0 | 0.01244615 | 0.01186441 | 0.00042526 |
| Rothia mucilaginosa | 0 | 0 | 0 | 0.00347056 | 0.01625125 | 0.00063789 |
| Staphylococcus aureus | 0 | 0 | 0 | 0.00155577 | 0.00179462 | 0.00021263 |
| Vibrio harveyi | 0 | 0 | 0 | 0.00143609 | 0.00089731 | 0.0323198 |
| Micrococcus luteus | 0 | 0 | 0 | 0.00071805 | 0.00159521 | 0.00042526 |
| Rothia dentocariosa | 0 | 0 | 0 | 0.00059837 | 0.00119641 | 0.00063789 |
| Rothia aeria | 0 | 0 | 0 | 0.00035902 | 0.0002991 | 0.00063789 |
| Brevundimonas poindexterae | 0 | 0 | 0 | 0.00035902 | 0.0001994 | 0.00021263 |
| Marinomonas primoryensis | 0 | 0 | 0 | 0.00011967 | 0.0003988 | 0.0021263 |

**Supplementary Table S11.** Linear Discriminant Analysis of enriched taxonomies in cultured healthy and diseased intestine using LefSe.

**Diseased**

| **Phyla** | **LDA** |
|---|---|
| NS | |
| **Class** | **LDA** |
| NS | |
| **Order** | **LDA** |
| NS | |
| **Family** | **LDA** |
| f_Halomonadaceae | 2.421 |
| **Genus** | **LDA** |
| g_Clostridium | 2.147 |

**Healthy**

| **Phyla** | **LDA** |
|---|---|
| NS | |
| **Class** | **LDA** |
| c_Flavobacteriia | 2.382 |
| **Order** | **LDA** |
| o_Xanthomonadales | 2.464 |
| o_Flavobacteriales | 2.349 |
| **Family** | **LDA** |
| f_Coxiellaceae | 2.539 |
| f_Flavobacteriaceae | 2.395 |
| f_Xanthomonadaceae | 2.267 |
| f_Piscirickettsiaceae | 2.234 |
| f_Rhodothermaceae | 2.222 |
| **Genus** | **LDA** |
| g_Pantoea | 2.526 |
| g_Umboniibacter | 2.233 |

**Supplementary Table S12**. Linear Discriminant Analysis of enriched taxonomies in cultured healthy and diseased hepatopancreas using LefSe.

**Diseased**

| **Phyla** | **LDA** |
|---|---|
| p_Spirochaetes | |
| p_Fusobacteria | |
| p_Verrucomicrobia | |

| **Class** | **LDA** |
|---|---|
| c_Leptospirae | 4.096 |
| c_Fusobacteriia | 3.650 |
| c_Deltaproteobacteria | 3.640 |
| c_Planctomycetia | 2.841 |

| **Order** | **LDA** |
|---|---|
| o_Leptospirales | 4.111 |
| o_Alteromonadales | 4.080 |
| o_Chromatiales | 3.828 |
| o_Fusobacteriales | 3.598 |
| o_Desulfobacterales | 3.563 |
| o_Oceanospirillales | 3.319 |
| o_Aeromonadales | 3.082 |
| o_R76_B128 | 2.913 |
| o_Planctomycetales | 2.865 |
| o_NB1_j | 2.558 |
| o_Rickettsiales | 2.472 |

| **Family** | **LDA** |
|---|---|
| f_OM60 | 3.767 |
| f_Fusobacteriaceae | 3.550 |
| f_Desulfobulbaceae | 3.359 |
| f_Aeromonadaceae | 3.077 |
| f_Rhizobiaceae | 2.882 |
| f_Planctomycetaceae | 2.863 |
| f_JTB38 | 2.706 |
| f_Porphyromonadaceae | 2.650 |
| f_Pelagibacteraceae | 2.612 |
| f_Phyllobacteriaceae | 2.468 |
| f_Geobacteraceae | 2.396 |

**Healthy**

| **Phyla** | **LDA** |
|---|---|
| N/S | |

| **Class** | **LDA** |
|---|---|
| c_TK17 | 2.826 |

| **Order** | **LDA** |
|---|---|
| N/S | |

| **Family** | **LDA** |
|---|---|
| f_Microbacteriaceae | 3.236 |
| f_Peptococcaceae | 2.435 |

| **Genus** | **LDA** |
|---|---|
| g_Propionigenium | 3.243 |
| g_Aeromonas | 3.097 |
| g_Teredinibacter | 2.978 |
| g_Planctomyces | 2.858 |
| g_Microbispora | 2.839 |
| g_Desulfotalea | 2.818 |
| g_Shinella | 2.777 |
| g_Simiduia | 2.714 |
| g_Parabacteroides | 2.648 |
| g_Thiothrix | 2.590 |

| **Genus** | **LDA** |
|---|---|
| g_Idiomarina | 2.591 |
| g_Faecalibacterium | 2.537 |
| g_Clostridiisalibacter | 2.317 |

**Supplementary Table S13.** Linear Discriminant Analysis of enriched taxonomies in cultured healthy and diseased sediment usir

| Diseased | | Healthy | |
|---|---|---|---|
| **Phyla** | **LDA** | **Phyla** | **LDA** |
| p_Spirochaetes | 2.967 | p_Chlamydiae | 2.630 |
| p_Verrucomicrobia | 2.681 | | |
| | | | |
| **Class** | **LDA** | **Class** | **LDA** |
| c_Spirochaetes | 2.858 | c_Acidimicrobiia | 4.107 |
| c_Leptospirae | 2.803 | c_BPC102 | 3.477 |
| c_OPB41 | 2.664 | c_Gemm_4 | 2.910 |
| | | c_Thermoleophilia | 2.823 |
| | | c_Chlamydiia | 2.709 |
| | | c_RB25 | 2.689 |
| | | | |
| **Order** | **LDA** | **Order** | **LDA** |
| o_Desulfobacterales | 3.859 | o_Pseudomonadales | 4.524 |
| o_Myxococcales | 3.092 | o_Acidimicrobiales | 4.135 |
| o_Rickettsiales | 2.786 | o_B110 | 3.465 |
| o_Leptospirales | 2.779 | o_Marinicellales | 3.271 |
| | | o_Burkholderiales | 3.056 |
| | | o_Chlamydiales | 2.681 |
| | | | |
| **Family** | **LDA** | **Family** | **LDA** |
| f_Desulfobulbaceae | 3.663 | f_Moraxellaceae | 4.438 |
| f_Desulfobacteraceae | 3.466 | f_koll13 | 3.990 |
| f_Nannocystaceae | 3.060 | f_Corynebacteriaceae | 3.557 |
| f_Sinobacteraceae | 2.763 | f_Marinicellaceae | 3.310 |
| f_Desulfuromonadaceae | 2.605 | f_Nocardioidaceae | 2.844 |
| f_Francisellaceae | 2.556 | f_Criblamydiaceae | 2.542 |
| | | | |
| **Genus** | **LDA** | **Genus** | **LDA** |
| g_Sediminimonas | 3.087 | g_Acinetobacter | 4.424 |
| g_Plesiocystis | 3.035 | g_Microbulbifer | 3.682 |
| g_Desulfococcus | 2.990 | g_Corynebacterium | 3.547 |
| g_Flavobacterium | 2.943 | g_Enhydrobacter | 2.736 |
| g_Desulfotignum | 2.905 | g_Yonghaparkia | 2.595 |
| g_Inquilinus | 2.804 | | |
| g_Halorhodospira | 2.667 | | |
| g_Desulfobacter | 2.609 | | |
| g_Acidaminobacter | 2.571 | | |
| g_Alishewanella | 2.554 | | |
| g_Salinivibrio | 2.524 | | |
| g_Salinibacter | 2.501 | | |

**Supplementary Table S14.** Relative frequency of unique genera and species in sequenced healthy and diseased samples.

| **genera_healthy_vs_dis_intestine** | | | intestine | | | |
|---|---|---|---|---|---|---|
| Treatment | | healthy cultured | | | diseased cultured | |
| Description | 139-i | 68B-i | 7A-i | 27B-i | 2B-i | 49B-i |
| g__Crenothrix | 0.000580046 | 0.00013257 | 0.00012945 | 0 | 0 | 0 |
| g__Microbispora | 0 | 0 | 0 | 0.00017479 | 0.00013238 | 0.00017153 |
| g__Shinella | 0 | 0 | 0 | 0.00017479 | 0.00013238 | 0.00017153 |
| g__Teredinibacter | 0 | 0 | 0 | 0.00017479 | 0.00013238 | 0.00017153 |

**Supplementary Table S14.** Relative frequency of unique genera and species in sequenced healthy and diseased samples.

| **spp__healthy_vs_dis_intestine** | | | | intestine | | | |
|---|---|---|---|---|---|---|---|
| Treatment | Treatment | | healthy cultured | | | diseased cultured | |
| Description | Description | 139-i | 68B-i | 7A-i | 27B-i | 2B-i | 49B-i |
| g__Microbispora | s__rosea | 0 | 0 | 0 | 0.00017479 | 0.00013238 | 0.00017153 |
| g__Teredinibacter | s__turnerae | 0 | 0 | 0 | 0.00017479 | 0.00013238 | 0.00017153 |

**Supplementary Table S14.** Relative frequency of unique genera and species in sequenced healthy and diseased samples.

| **genera_healthy_vs_dis_hepatopancreas** | | | hepatopancreas | | | |
|---|---|---|---|---|---|---|
| Treatment | | healthy cultured | | | diseased cultured | |
| Description | 139-H | 68B-H | 7A-H | 27B-H | 2B-H | 49B-H |
| g__Escherichia | 0 | 0 | 0 | 0.00019429 | 0.00025272 | 0.00015411 |

**Supplementary Table S14.** Relative frequency of unique genera and species in sequenced healthy and diseased sample

| **spp_healthy_vs_dis_hepatopancreas** | | | hepatopancreas | | | |
|---|---|---|---|---|---|---|
| Treatment | | healthy cultured | | | diseased cultured | |
| Description | 139-H | 68B-H | 7A-H | 27B-H | 2B-H | 49B-H |
| g__Escherichia blattae | 0 | 0 | 0 | 0.00019429 | 0.00025272 | 0.00015411 |

**Supplementary Table S14**. Relative frequency of unique genera and species in sequenced healthy and diseased samples.

| **genera_healthy_vs_dis_sediment** | | | pond sediment | | | |
|---|---|---|---|---|---|---|
| Treatment | | healthy cultured | | | diseased cultured | |
| Description | 139-S | 68B-S | 7A-S | 27B-S | 2B-S | 49B-S |
| g__Pseudomonas | 0.00013868 | 0.00096899 | 0.00048123 | 0 | 0 | 0 |
| g__Inquilinus | 0 | 0 | 0 | 0.00127199 | 0.00070771 | 0.0009113 |
| g__Desulfobacter | 0 | 0 | 0 | 0.000212 | 0.00017693 | 0.00060753 |
| g__Salinibacter | 0 | 0 | 0 | 0.000106 | 0.00053079 | 0.00060753 |
| g__Flavobacterium | 0 | 0 | 0 | 0.000106 | 0.004954 | 0.00030377 |
| g__Halothiobacillus | 0 | 0 | 0 | 0.000106 | 0.00053079 | 0.00030377 |
| g__Acidaminobacter | 0 | 0 | 0 | 0.000106 | 0.00017693 | 0.00030377 |
| g__Alishewanella | 0 | 0 | 0 | 0.000106 | 0.00017693 | 0.00030377 |
| g__Salinivibrio | 0 | 0 | 0 | 0.000106 | 0.00017693 | 0.00030377 |

**Supplementary Table S14.** Relative frequency of unique genera and species in sequenced healthy and diseased samples

| **spp_healthy_vs_dis_sediment** | | | | sediment | | | |
|---|---|---|---|---|---|---|---|
| Treatment | | | healthy cultured | | | diseased cultured | |
| Description | | 139-S | 68B-S | 7A-S | 27B-S | 2B-S | 49B-S |
| g__Pseudomonas | s__veronii | 0.00013868 | 0.00096899 | 0.00048123 | 0 | 0 | 0 |
| g__Salinivibrio | s__costicola | 0 | 0 | 0 | 0.000106 | 0.00017693 | 0.00030377 |

**Supplementary Table S15.** AP3 PCR diagnostic results.

| **Sample** | | **AP3 test** |
|---|---|---|
| wt intestine | IS | EMS- |
| | 1IS | EMS- |
| | 2IS | EMS- |
| healthy cultured intestine | 7A-I | EMS+ |
| | 68B-I | EMS- |
| | 139-I | EMS- |
| diseased cultured intestine | 49B-I | EMS+ |
| | 27B-I | EMS+ |
| | 2B-I | EMS- |
| wt hepatopancreas | HS | EMS- |
| | 3HS | EMS- |
| | 1HS | ND |
| healthy cultured hepatopancreas | 7A-H | EMS+ |
| | 68B-H | EMS+ |
| | 139-H | EMS+ |
| diseased cultured hepatopancreas | 49B-H | EMS+ |
| | 27B-H | EMS+ |
| | 2B-H | EMS+ |
| healthy pond sediment | 7A-S | ND |
| | 68B-S | ND |
| | 139-S | ND |
| diseased pond sediment | 27B-S | ND |
| | 49B-S | ND |
| | 2B-S | ND |

ND= Not determined because the DNA amount was not suficient
EMS+ = positive to AP3 PCR test
EMS- = negative to AP3 PCR test

**Supplementary Table S16**. Relative abundance of 7 age discriminating OTUs present in the sequenced samples.

| # Constructed from biom file | Intestine wt | | | healthy cultured intestine | | | diseased cultured intestine | | | Hepatopancreas wt | | | healthy cultured hepatopancreas | | | diseased cultured hepatopancreas | | |
|---|---|---|---|---|---|---|---|---|---|---|---|---|---|---|---|---|---|---|
| #OTU ID | i 1 wt | i 2 wt | i 3 wt | 139-i | 68B-i | 7A-i | 49B-i | 27B-i | 2B-i | h 1 wt | h 2 wt | h 3 wt | 139-H | 68B-H | 7A-H | 49B-H | 27B-H | 2B-H |
| k__Bacteria; p__Proteobacteria; c__Alphaproteobacteria; o__Rhodobacterales; f__Rhodobacteraceae; g__Nautella; s__italica | 0 | 0 | 0 | 0 | 0.00013257 | 0 | 0 | 0.00017479 | 0 | 0 | 0 | 0 | 0 | 0 | 0 | 0 | 0 | 0 |
| k__Bacteria; p__Proteobacteria; c__Gammaproteobacteria; o__Enterobacteriales; f__Enterobacteriaceae; g__; s__ | 0.00094853 | 0 | 0.00018651 | 0 | 0.00013257 | 0 | 0 | 0.00017479 | 0 | 0.00011967 | 0 | 0 | 0 | 0 | 0 | 0 | 0 | 0.00353803 |
| k__Bacteria; p__Proteobacteria; c__Alphaproteobacteria; o__Rhodobacterales; f__Rhodobacteraceae; g__; s__ | 0 | 0 | 0 | 0 | 0.00026515 | 0.0002589 | 0.00051458 | 0 | 0.00026476 | 0 | 0 | 0 | 0 | 0 | 0 | 0 | 0 | 0 |
| k__Bacteria; p__Proteobacteria; c__Gammaproteobacteria; o__Vibrionales; f__Pseudoalteromonadaceae; g__; s__ | 0 | 8.82924E-05 | 0.00027977 | 0 | 0.00053029 | 0.00012945 | 0 | 0 | 0.00039714 | 0 | 9.97009E-05 | 0 | 0 | 0 | 0 | 0 | 0 | 0 |
| k__Bacteria; p__Proteobacteria; c__Alphaproteobacteria; o__Rhodobacterales; f__Rhodobacteraceae; g__; s__ | 0.00012647 | 0 | 0 | 0 | 0 | 0 | 0 | 0 | 0 | 0 | 0 | 0 | 0 | 0 | 0 | 0 | 0 | 0 |
| k__Bacteria; p__Proteobacteria; c__Alphaproteobacteria; o__Rhodobacterales; f__Rhodobacteraceae; g__Dinoroseobacter; s__ | 6.3235E-05 | 0 | 0 | 0 | 0.00026515 | 0 | 0.00102916 | 0.0033211 | 0.00026476 | 0 | 0 | 0 | 0.00027027 | 0 | 0.00022462 | 0 | 0.00019429 | 0 |
| k__Bacteria; p__Proteobacteria; c__Gammaproteobacteria; o__Vibrionales; f__Vibrionaceae; g__Vibrio; s__shilonii | 0 | 0.000264877 | 0.00018651 | 0.00058005 | 0.00092801 | 0.0005178 | 0 | 0 | 0 | 0 | 0 | 0 | 0.00027027 | 0 | 0 | 0 | 0 | 0 |

**Supplementary Table S17.** Weight of the wild-type and cultured shrimps used for the extraction of sequenced intestines and hepatopancreas.

| Wild-type shrimps | | | | One-way ANOVA |
|---|---|---|---|---|
| | Weight (g) | Average weight (g) | Std.Dev | Significant? $p<0.05$ |
| 1 wt | 41.4 | | | |
| 2 wt | 43.6 | 43.8 | 2.1 | NO |
| 3 wt | 46.5 | | | |

| Healthy cultured shrimps | | | | One-way ANOVA |
|---|---|---|---|---|
| | Weight (g) | Average weight (g) | Std.Dev | Significant? $p<0.05$ |
| 139 | 15.9 | | | |
| 68B | 16.6 | 17.1 | 1.2 | NO |
| 7A | 18.7 | | | |

| Diseased cultured shrimps | | | | One-way ANOVA |
|---|---|---|---|---|
| | Weight (g) | Average weight (g) | Std.Dev | Significant? $p<0.05$ |
| 49B | 15.3 | | | |
| 27B | 15.8 | 15.3 | 0.4 | NO |
| 2B | 14.8 | | | |
